# Adipocytes influence choroidal neovascularization via PRDM16

**DOI:** 10.1038/s44321-026-00441-5

**Published:** 2026-05-19

**Authors:** Roberto Diaz-Marin, Masayuki Hata, Vera Guber, Vincent De Guire, Ariel M Wilson, Sergio Crespo-Garcia, Przemyslaw Sapieha

**Affiliations:** 1 Department of Biochemistry, Maisonneuve-Rosemont Hospital Research Centre, Université de Montréal, Montréal, QC Canada; 2 Department of Ophthalmology, Maisonneuve-Rosemont Research Centre, Université de Montréal, Montréal, QC Canada; 3 Present Address: School of Optometry, Université de Montréal, Montréal, QC Canada

**Keywords:** Metabolism, Vascular Biology & Angiogenesis

## Abstract

Neovascular age-related macular degeneration (nAMD) is a prominent cause of blindness in the elderly, characterized by pathological subretinal choroidal neovascularization (CNV). While age and genetics predispose to AMD, modifiable factors such as body adiposity are also thought to contribute. In a mouse model of nAMD, we identified a role for adipose tissue (AT) in exacerbating CNV, specifically pathways in adipocytes expressing *Prdm16*. Laser-induced CNV in the retina led to heightened expression of genes associated with browning and inflammation in distal inguinal white AT (iWAT). Selective deletion of the browning-associated transcription factor *Prdm16* in adipocytes inhibited AT browning and reduced CNV. Reintroduction of *Prdm16*-expressing adipose tissue was sufficient to aggravate CNV in *Prdm16*-deficient mice. Ex vivo experimentation suggested that *Prdm16*-expressing adipocytes secrete angiogenic factors such as IGFBP5 and contribute to pathological angiogenesis. Thus, *Prdm16*-expressing adipocytes contribute to CNV, highlighting communication between the retina and distal tissues in the pathogenesis of AMD.

## Synopsis

Wet age-related macular degeneration is characterized by choroidal neovascularization (CNV). Triggering CNV leads to remodeling of distal depots of inguinal adipose tissue, a process that can impact the severity of disease.

- Local injury in the retina triggers systemic changes, including remodeling of distal adipose tissue.
- PRDM16 (associated with function of brown and beige adipocytes) in adipocytes contributes to CNV.
- Depleting PRDM16 from adipocytes reduces CNV.

## Abstract (web summary)

Wet age-related macular degeneration is characterized by choroidal neovascularization (CNV). Triggering CNV leads to remodeling of distal depots of inguinal adipose tissue, a process that can impact the severity of disease.

## Graphical abstract

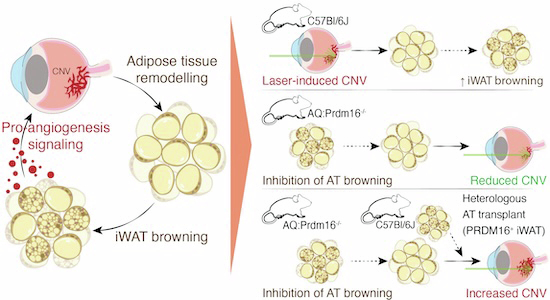

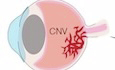

The paper explained **Problem**

Age-related macular degeneration (AMD) is a slow progressing degenerative retinal disease and the leading cause of vision loss in the elderly. The neovascular form of AMD (nAMD) accounts for up to 90% of legal blindness from AMD cases and is characterized by abnormal proliferation of destructive blood vessels from the choriocapillaris (choroidal neovascularization; CNV). Modifiable risk factors such as obesity can predispose to nAMD. To date, links between disease severity and changes in the state of adipose tissue cellular composition and function, such as may occur during later life, remain relatively unknown.

**Results**

We investigated how triggering CNV in a laser-induced mouse model influenced adipose tissue (AT) remodeling and, conversely, how AT remodeling contributes to CNV. We observed that CNV triggered molecular pathways associated with adipose tissue browning and specifically the transcription factor PR/SET Domain 16 (PRDM16) in inguinal white adipose tissue (iWAT). Genetic ablation of *Prdm16* in adipocytes inhibited AT browning and reduced CNV.

**Impact**

Our study describes an axis between the retina and adipose tissue where, during retinal neovascularization, iWAT undergoes tissue remodeling that ultimately influences CNV. *Prdm16*-expressing adipocytes contribute to CNV, highlighting communication between the retina and distal tissues in the pathogenesis of AMD.

## Introduction

Age-related macular degeneration (AMD) is a degenerative retinal disease and the leading cause of vision loss in the elderly (Gehrs et al, 2006), with a prevalence estimated at 196 million in 2020 and expected to reach 288 million by 2040 (Wong et al, 2014). The neovascular form of AMD (nAMD) accounts for 90% of legal blindness from AMD cases (Ferris et al, 1984). Neovascular AMD is characterized by abnormal proliferation of destructive blood vessels from the choriocapillaris (choroidal neovascularization; CNV), with subsequent disruption of the photoreceptor organization and the deterioration of central vision (Guillonneau et al, 2017). While anti-VEGF therapies have transformed the treatment of nAMD (Apte et al, 2019), they are palliative, and hence gaining a deeper understanding of how risk factors converge to trigger disease may provide a path towards prevention and reduction of incidence. Multiple non-modifiable and modifiable risk factors predispose to nAMD, including age (Ehrlich et al, 2008), genetics (Fritsche et al, 2016; Swaroop et al, 2007), smoking (Chakravarthy et al, 2010), gut microbiota (Andriessen et al, 2016; Zinkernagel et al, 2017), and abdonimal obesity (Adams et al, 2011). The latter suggests a potential role for adipose tissue (AT), a link substantiated by higher body-mass index (BMI > 30) being associated with progression to late AMD (Seddon et al, 2020). Furthermore, patients living with AMD have more visceral adipose tissue (AT), and an increase in abdominal fat is significantly associated with AMD (Haas et al, 2015).

Importantly, adipose tissue remodels and influences systemic metabolic health during obesity and dyslipidemia (Bluher, 2019; Boden, 2008). AT depots are not homogenous throughout the body and differ in cellular composition and function (Chait and den Hartigh, 2020; Fruhbeck, 2008). AT can be primarily categorized by two major depots: white adipose tissue (WAT) that regulates energy homeostasis via storage and release of lipids (Fruhbeck, 2008) and brown adipose tissue (BAT) that participates in non-shivering thermogenesis (Smith, 1964). While the size and number of WAT depots augment during obesity, other types of depots, such as BAT, undergo a significant reduction, similarly to what occurs with aging (Zoico et al, 2019). BAT regulates non-shivering thermogenesis via uncoupling protein 1 (UCP1) in the mitochondria through a process known as uncoupled respiration (Smith, 1964). UCP1 uncouples energy generated from fatty acid oxidation (FAO) and redirects production of adenosine triphosphate (ATP) towards heat production (Smith, 1964). A third intermediate subtype of AT known as the beige adipose tissue (BgAT) participates in non-shivering thermogenesis via AT browning (Choe et al, 2016; Wu et al, 2012). AT browning is defined as the reversible conversion of white adipocytes into beige adipocytes in response to different stimuli such as cold exposure (Cousin et al, 1992; Young et al, 1984), exercise (Bostrom et al, 2012), fibroblast growth factor 21 (FGF21) (Coskun et al, 2008; Fisher et al, 2012), and more. Since BAT activity and AT browning are decreased naturally during obesity, it has been hypothesized that AT browning could counter obesity by increasing the amount of thermogenically active AT (Kurylowicz and Puzianowska-Kuznicka, 2020; Sidossis and Kajimura, 2015; Zoico et al, 2019), yet the contribution of BAT to ocular pathologies is less understood. The most proximal AT to the retina is the retro-orbital AT (rOAT) (Bahn, 2010; Zhang et al, 2020) that acts as structural support to maintain the ocular globe in place. Recent studies have shown that the rOAT can undergo AT browning to regulate eye temperature (Sugiyama et al, 2019). Here, we investigated how triggering CNV with a laser-induced mouse model influenced AT remodeling. Conversely, we studied how AT browning contributes to CNV.

## Results

### Laser-induced choroidal neovascularization triggers a response in distal adipose tissues

**Figure 1:**
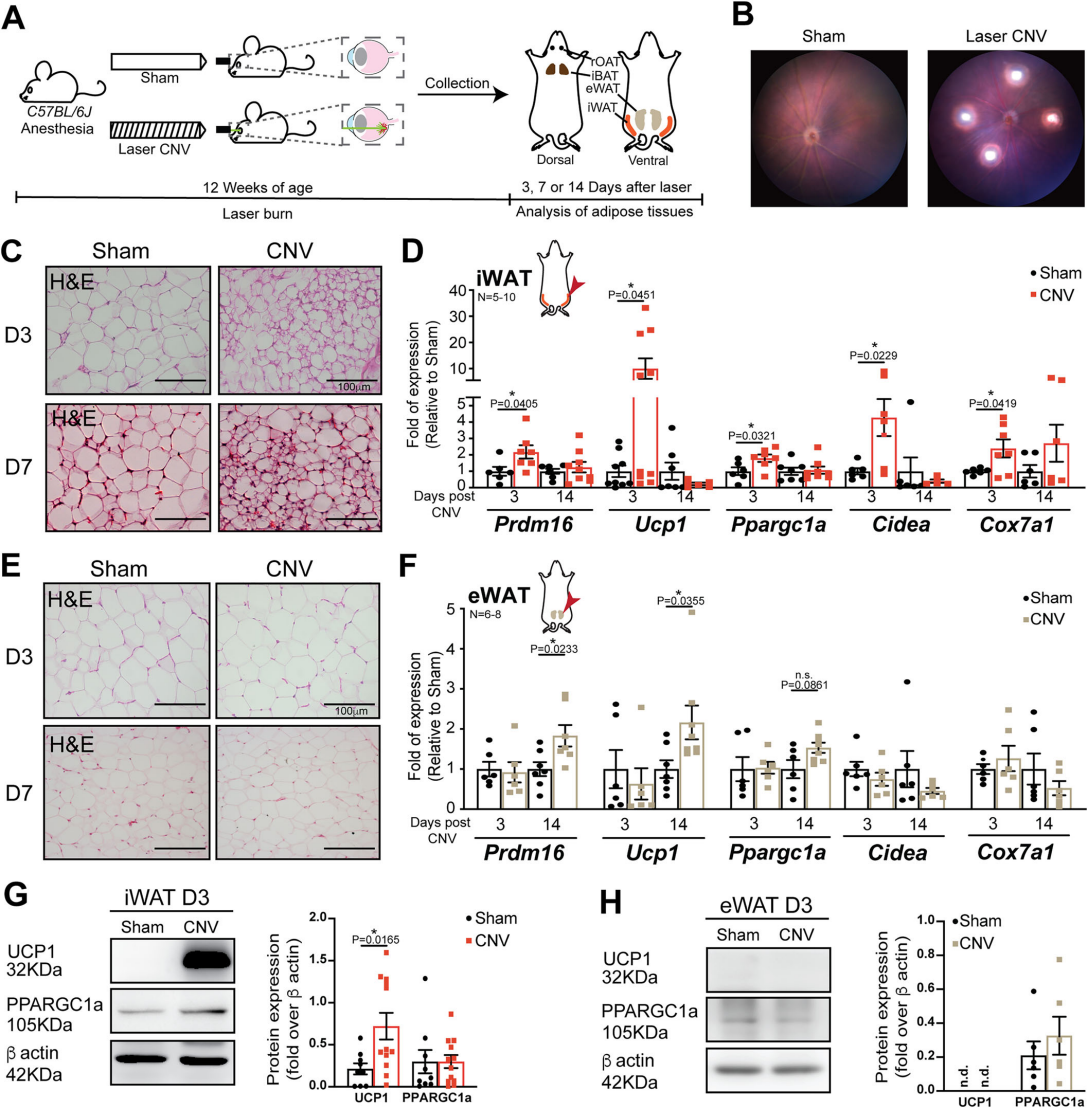
Laser-induced choroidal neovascularization activates inguinal WAT. (**A**) Schematic representation of experimental time course. C57BL/6J mice were anesthetized, and mice were either subjected to 4 laser burns per eye (400 mW, 50 µm, 0.05 s) with an argon laser (laser CNV group) or did not receive laser burns (sham group) but were handled. In all, 3, 7, or 14 days after laser burn, mice were euthanized, and ATs were collected for analysis. (**B**) Representative retinal fundus of sham (left) and laser CNV (right) mice. (**C**) Representative H&E staining of iWAT from sham and laser CNV C57Bl/6J mice, 3- and 7-days after laser burn, *n*  =  3–5 mice per group. (**D**) iWAT relative mRNA expression of *Prdm16*, *Ucp1, Ppargc1a*, *Cidea* and *Cox7a1* from laser CNV and sham C57BL/6J mice. Gene expression was normalized to sham mice for every timepoint 3 and 14 days after laser burn, *n* = 7–9 mice per group. (**E**) Representative H&E staining of eWAT from sham and laser CNV C57BL/6 J mice, 3- and 7-days after laser burn, *n*  =  3–5 mice per group. (**F**) eWAT relative mRNA expression of *Prdm16*, *Ucp1, Ppargc1a*, *Cidea* and *Cox7a1* from sham and laser CNV C57BL/6J mice. Gene expression was normalized to sham mice for every timepoint 3 and 14 days after laser-burn, *n* = 7–9 mice per group. (**G**, **H**) Representative immunoblots and their corresponding quantification for iWAT (**G**) and eWAT (**H**) levels of UCP1 and PPARGC1a at 3 days post laser in laser CNV and sham group (*n* = 6–12 per group). β-actin was used as a loading control. Data is presented as mean ± SEM. Statistical significance was assessed using unpaired two-tailed Student’s *t* test. Exact *P* values are indicated in the figure, with *P < 0.05. Source data are available online for this figure.

**Figure EV1:**
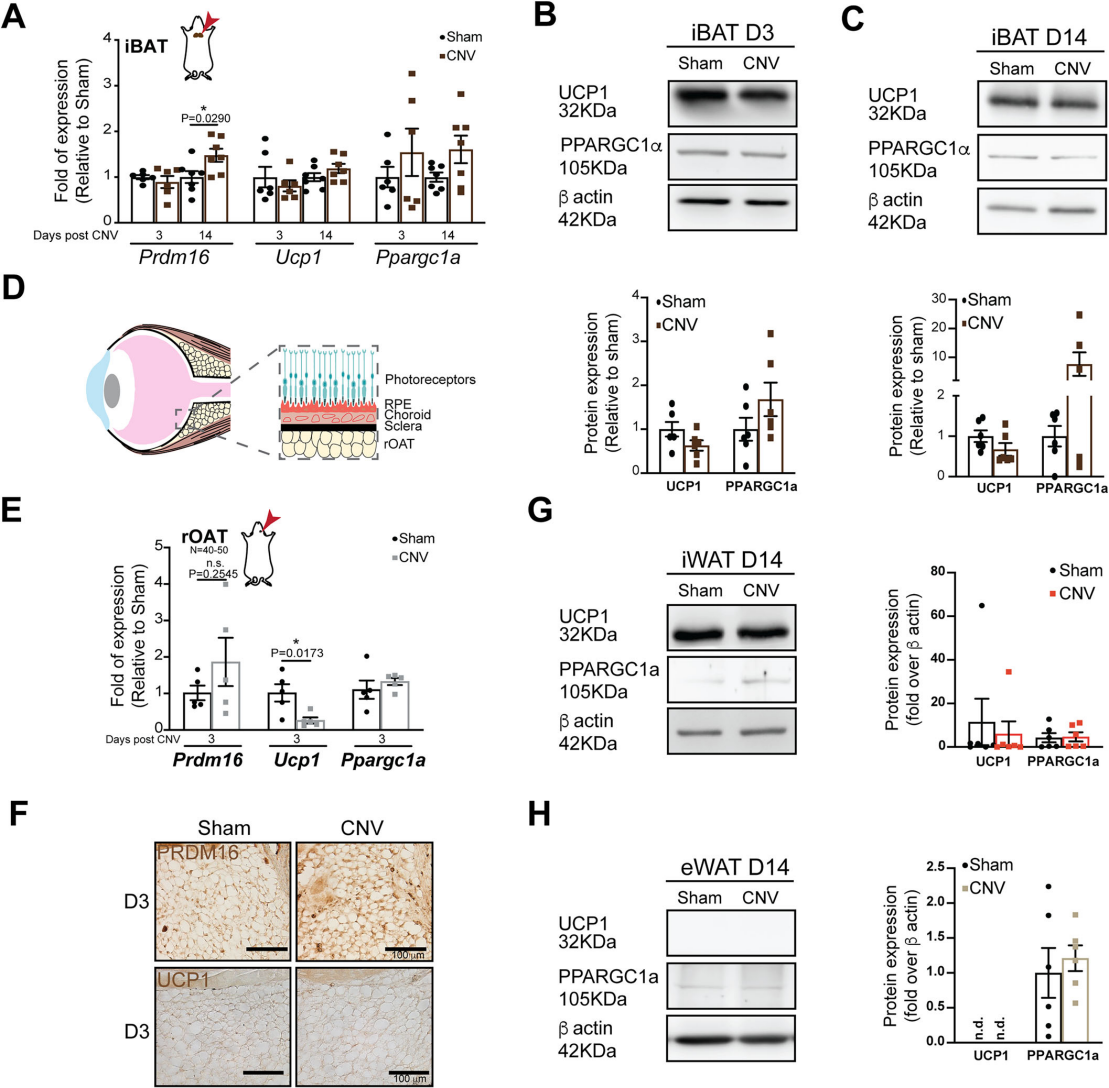
Thermogenic factor expression in iBAT is not affected by CNV. (**A**) iBAT relative mRNA expression of *Prdm16*, *Ucp1* and *Ppargc1a* from laser CNV and sham C57BL/6J mice 3- and 14-days post CNV, *n* = 7 mice per group. Gene expression was normalized to sham mice 3- and 14-days post CNV. (**B**) Representative immunoblots and their corresponding quantification for UCP1 and PPARGC1a protein levels of iBAT at 3-days in laser CNV and sham group (*n* = 6 per group). (**C**) Representative immunoblots and their corresponding quantification for UCP1 and PPARGC1a protein levels in iBAT 14-days in laser CNV and sham group (*n* = 6 per group). β-actin was used as a loading control. (**D**) Schematic representation of the anatomical location of rOAT. The rOAT is located around the optic nerve behind the posterior pole of the eyeball in contact with the sclera. (**E**) rOAT relative mRNA expression of *Prdm16*, *Ucp1* and *Ppargc1a* from laser CNV and sham C57BL/6J mice at 3 days post CNV. Gene expression was normalized to sham mice, *n* = 40–50 mice per group. (**F**) Representative immunohistochemistry staining images for PRDM16 and UCP1 in rOAT from laser CNV and sham C57BL/6J mice at 3 days post CNV, *n* = 5 mice per group. (**G**, **H**) Representative immunoblots and their corresponding quantification for UCP1 and PPARGC1a protein levels in (**G**) iWAT and (**H**) eWAT 14-days in laser CNV and sham group (*n* = 6 per group). β-actin was used as a loading control. Data is presented as mean ± SEM. Statistical significance was assessed using unpaired two-tailed Student’s *t* test. Exact *P* values are indicated in the figure, with *P < 0.05. Source data are available online for this figure.

To investigate a potential link between pathological choroidal neovascularization (CNV) and AT remodeling, we performed argon laser-induced photocoagulation on C57BL/6 J male mice to rupture Bruch’s membrane and provoke pathological choroidal angiogenesis (Lambert et al, 2013; Liu et al, 2017). Mice received 4 distinct burns in each eye and were compared to identically handled sham-treated mice (Fig. 1A). Disruption of Bruch’s membrane was verified by the formation of a bubble via fundus photography (Fig. 1B). While the laser CNV model does not recapitulate the protracted age-dependent nature of human neovascular AMD, it models aspects of human disease with regard to the inflammation-driven neoangiogenesis of the choroid. It was selected for its superior reproducibility and lesion incidence compared to transgenic or aging mouse strains that exhibit spontaneous CNVs (Grossniklaus et al, 2010). We investigated inguinal WAT (iWAT) from bilateral superficial subcutaneous deposits between skin and muscle fascia anterior to the lower segment of hind limbs; epididymal WAT (eWAT) from the bilateral intra-abdominal visceral depots attached to the epididymis as well as interscapular BAT (iBAT) from bilobed tissue between the scapulae (Fig. 1A). Histological assessment of iWAT showed a reduction in adipocyte size at both 3- and 7-days post CNV (Fig. 1C). These phenotypical changes were accompanied by a significant increase in expression of the browning markers *Prdm16*, *Ucp1, Ppargc1a, Cidea and Cox7a1* in iWAT 3 days after laser-induced CNV compared to sham mice. Expression of transcripts returned to basal levels 14 days post burn (Fig. 1D). Conversely to iWAT, histological assessment of eWAT did not show any differences between groups at earlier timepoints of 3- and 7-days post CNV (Fig. 1E) and transcripts for browning-associated genes remained unaltered 3 days post CNV (Fig. 1F). Significant changes appeared later, with transcripts for browning-associated genes *Prdm16* and *Ucp1* significantly upregulated in eWAT at 14 days post CNV (Fig. 1F). We also investigated brown adipose tissue (iBAT) and observed mild increases in transcripts for *Prdm16* at 14-days post CNV (Fig. EV1A–C). Complementarily, we assessed changes in retro-orbital AT (rOAT) after CNV (Fig. 1A). rOAT is located around the optic nerve and behind the posterior pole of the eyeball, and it has been reported to undergo browning (Sugiyama et al, 2019) (Fig. EV1D). Three days post-laser, we detected a significant reduction in expression of *Ucp1* transcripts in rOAT when compared to sham (Fig. EV1E). However, immunohistochemistry on rOAT did not reveal any phenotypic alteration or AT remodeling related to browning, nor changes in protein expression of UCP1, nor PRDM16 in adipocytes (Fig. EV1F). In line with gene expression, protein levels of UCP1 were upregulated in iWAT 3 days post laser but remained unaltered in eWAT (Fig. 1G,H). At 14 days post CNV, UCP1 protein expression returned to basal levels in iWAT and continued to remain unchanged in eWAT (Fig. EV1G,H). Together, these data suggest that following laser-induced CNV, distal AT depots undergo tissue remodeling and increased AT browning marker expression at different rates, with iWAT responding more rapidly than eWAT or iBAT.

### Laser-induced CNV provokes iWAT inflammation

**Figure 2:**
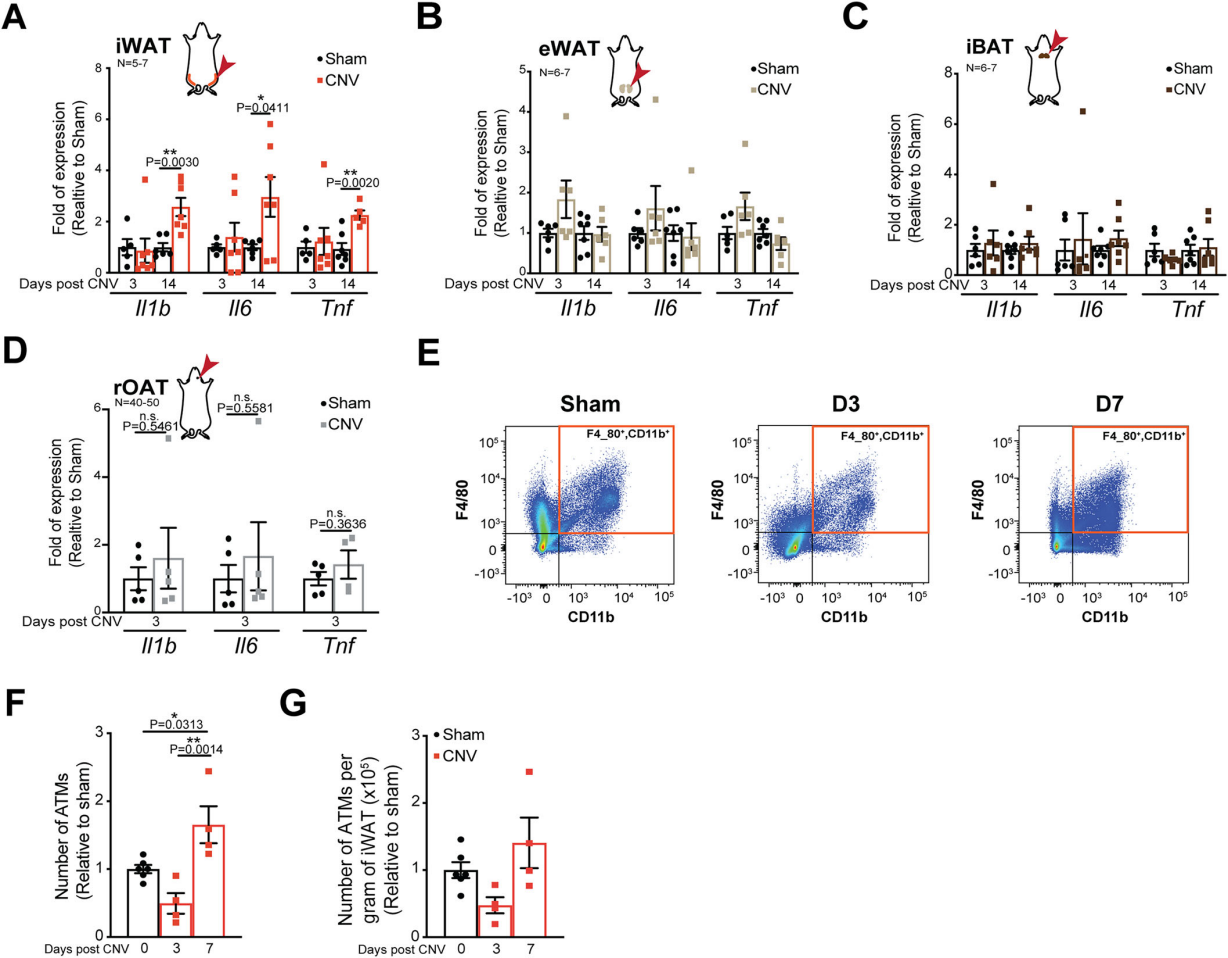
Laser-induced CNV induces iWAT inflammation. (**A**–**D**) Relative mRNA expression of *Il1b*, *Il6*, and *Tnf* from laser CNV and sham C57Bl/6J mice in (**A**) iWAT, (**B**) eWAT, (**C**) iBAT, and (**D**) rOAT. Gene expression was quantified from C57BL/6J mice 3- and 14-days after laser burn, *n* = 5–50 mice per group. mRNA levels were normalized to those of sham mice. (**E**) Representative flow cytometry analysis timecourse of iWAT ATMs, (**F**) quantification of the number of ATMs, and (**G**) total number of ATMs per gram of iWAT normalized to sham mice from laser CNV mice at 0, 3, and 7 days after laser burn (*n* = 4–6 per group). Data are presented as mean ± SEM. Statistical significance was assessed using unpaired two-tailed Student’s *t* test for (**A**–**D**) and ordinary one-way ANOVA followed by Tukey’s multiple comparisons post hoc test for (**F**, **G**). Exact *P* values are indicated in the figure, with *P < 0.05 and **P < 0.01. Source data are available online for this figure.

**Figure EV2:**
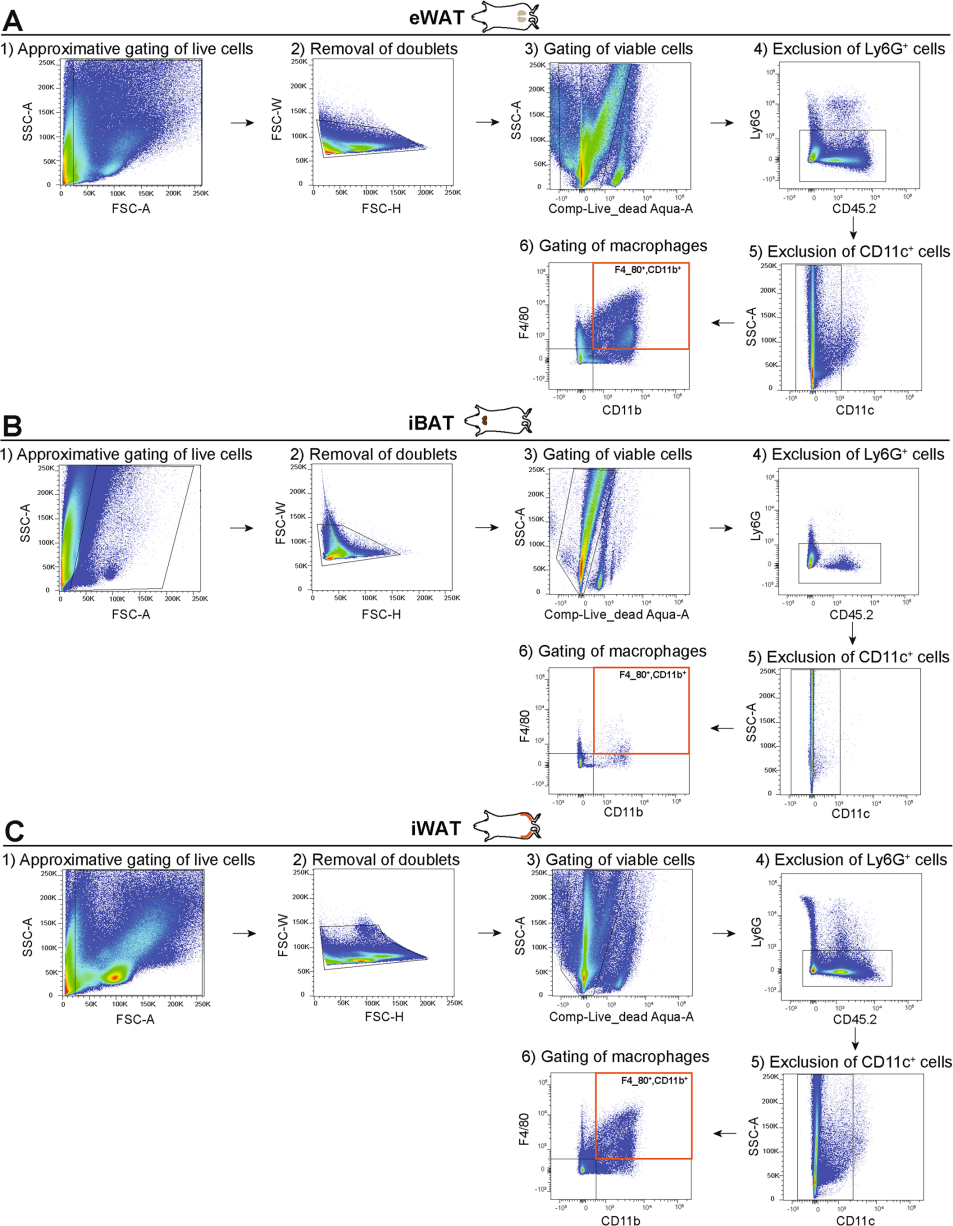
Flow cytometry gating strategies of ATMs from ATs. (**A**–**C**) Gating strategy for ATMs in the SVF of (**A**) eWAT, (**B**) iBAT, and (**C**) iWAT: (1) approximative gating of live cells, (2) gating to remove doublets, (3) selection of viable cells, (4) exclusion of Ly6G-positive cells, (5) exclusion of CD11c-positive cells, and (6) gating of ATMs.

**Figure EV3:**
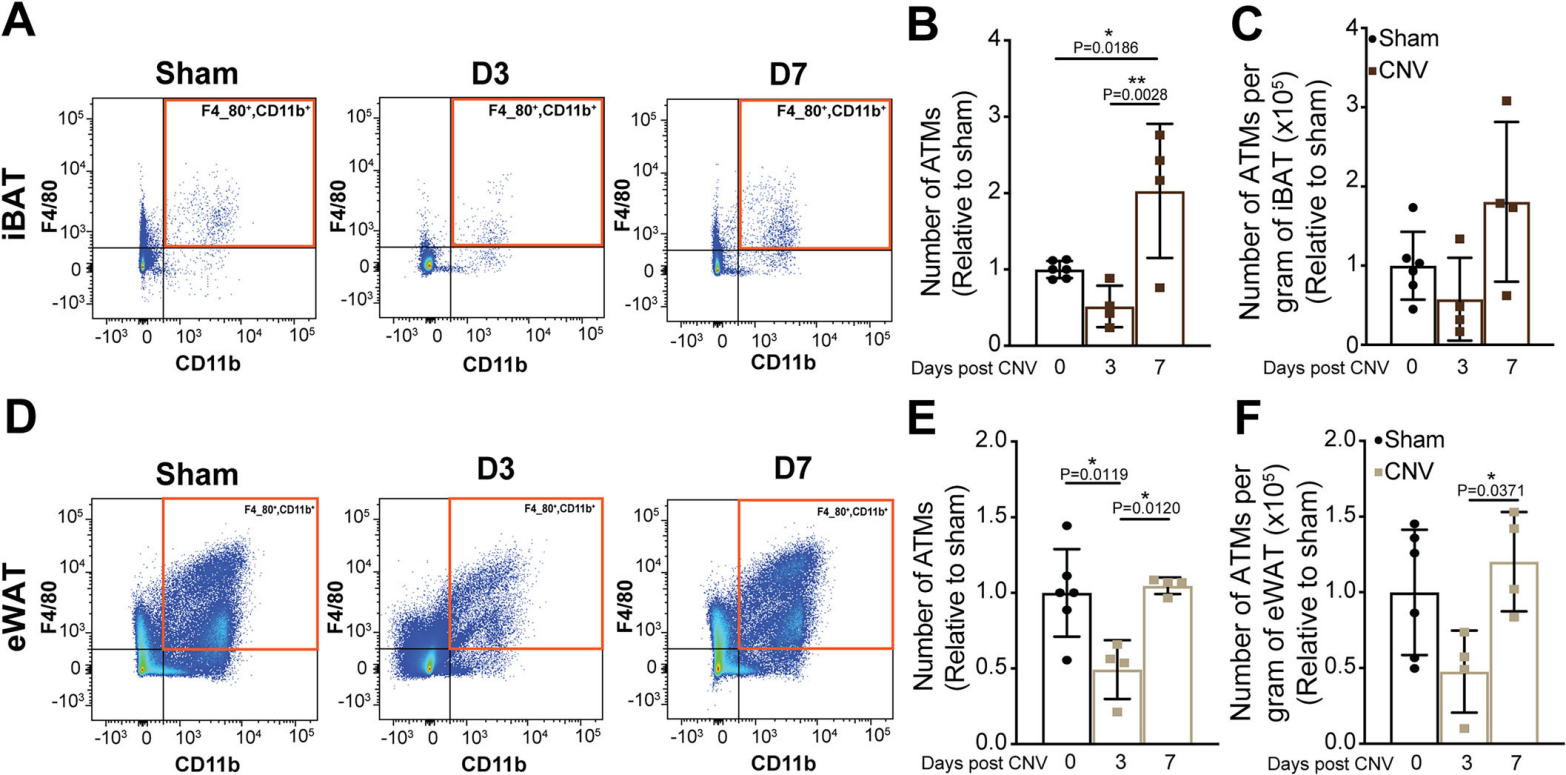
ATMs increase in iBAT during CNV formation. (**A**) Representative flow cytometry analysis time course of iBAT ATMs, (**B**) quantification of the number of ATMs, and (**C**) total number of ATMs per gram of iBAT normalized to sham mice from laser CNV mice at 0, 3, and 7 days after laser burn (*n* = 4–6 per group). (**D**) Representative flow cytometry analysis time course of eWAT ATMs, (**E**) quantification of the number of ATMs, and (**F**) total number of ATMs per gram of eWAT normalized to sham mice from laser CNV mice at 0, 3, and 7 days after laser burn (*n* = 4–6 per group). Data are presented as mean ± SEM. Statistical analysis was performed using ordinary one-way ANOVA followed by Tukey’s multiple comparisons post hoc test. Exact *P* values are indicated in the figure, with *P < 0.05 and **P < 0.01.

Monocyte mobilization to ocular tissue is a salient feature of AMD pathogenesis (Guillonneau et al, 2017; Nowak, 2006), and abdominal AT is a source of low-chronic inflammation in obesity (Castoldi et al, 2015) with potential impact on the retina (Andriessen et al, 2016; Hata et al, 2023; Sene et al, 2013). We therefore sought to determine if, during CNV progression, remodeling of AT and the increased expression of AT browning-related markers could influence CNV formation. Evaluation of pro-inflammatory genes in AT depots by qPCR revealed significant upregulation of interleukin-1b (*Il1b)*, interleukin-6 *(Il6)*, and tumor necrosis factor alpha (*Tnf)* restricted to iWAT at later stages of CNV remodeling (14 days following laser) (Fig. 2A). Conversely, a non-significant trend toward an increase in inflammatory markers was observed in eWAT and rOAT at 3 days after CNV, but inflammatory markers tended to subside 14 days post CNV (Fig. 2B–D). We next sought to investigate the contribution of adipose tissue macrophages (ATMs) (CD11b^+^F4/80^+^) in the stromal vascular fraction (SVF) of AT by flow cytometry (Fig. EV2A–C). The number of ATMs in both iWAT and iBAT increased significantly by day 7 (Figs. 2E–G and EV3A–C). In eWAT, we observed a drop in ATMs 3 days post-CNV and return to basal sham levels by day 7 post-CNV (Fig. EV3D–F). These results, therefore, suggest that induction of CNV stimulates the recruitment of ATMs to iWAT and iBAT.

### Laser-induced CNV provokes the rapid release of systemic catecholamines

**Figure EV4:**
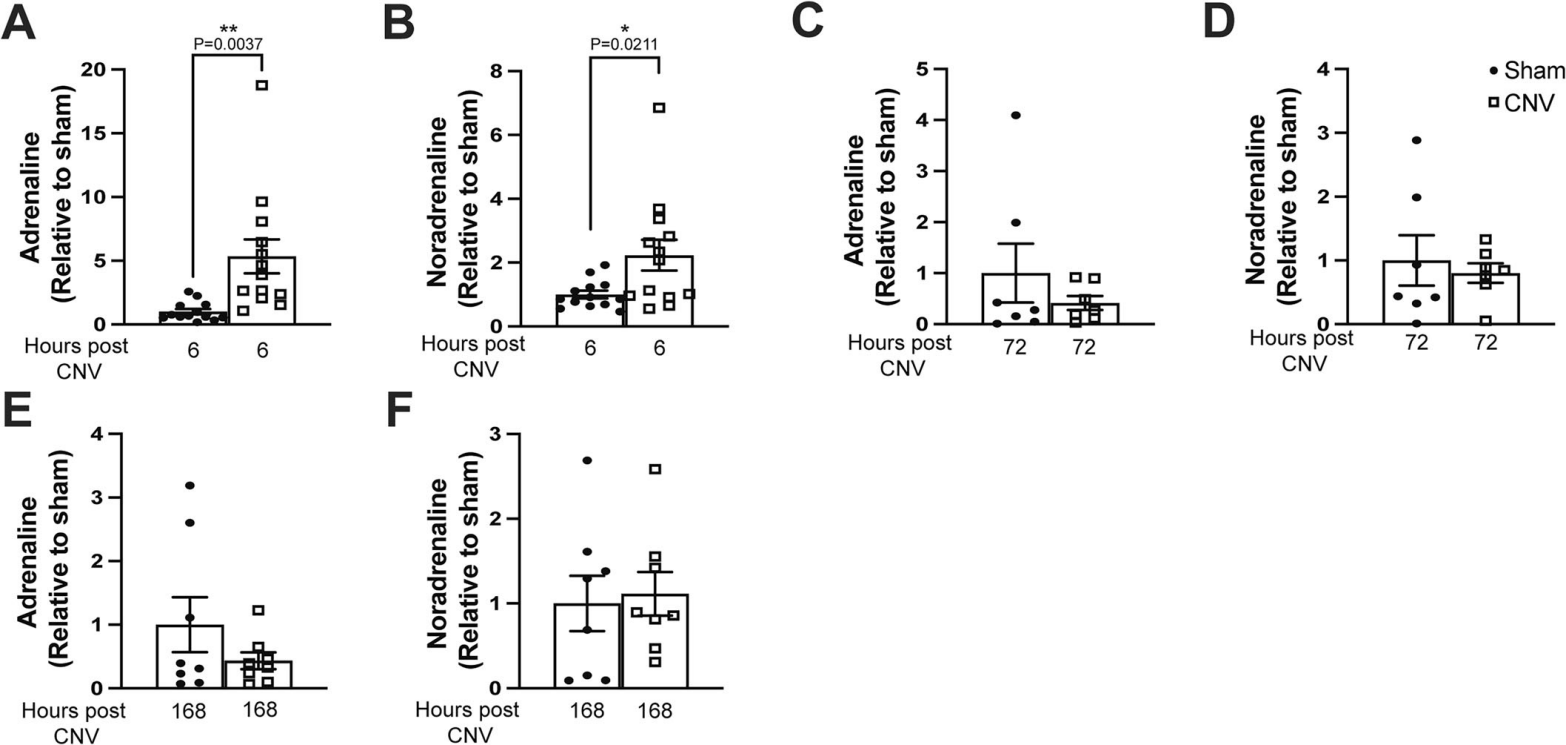
Plasma catecholamines are upregulated 6 h after laser treatment. (**A**) Adrenaline and (**B**) noradrenaline plasma levels from laser CNV and sham C57BL/6J mice 6 h post CNV, *n* = 13. (**C**) Adrenaline and (**D**) noradrenaline plasma levels from laser CNV and sham C57BL/6J mice 72 h post CNV, *n* = 7. (**E**) Adrenaline and (**F**) noradrenaline plasma levels from laser-burned and sham C57BL/6J mice 168 h post CNV, *n* = 8. Data are presented as mean ± SEM. Statistical significance was assessed using unpaired two-tailed Student’s *t* test. Exact *P* values are indicated in the figure, with *P < 0.05 and **P < 0.01. Source data are available online for this figure.

Systemic release of catecholamines by adrenal glands has been linked to AT browning in severely burned patients (Abdullahi et al, 2019; Patsouris et al, 2015). Given the induction of browning markers in iWAT during CNV, we measured concentrations of adrenaline and noradrenaline in plasma post-laser burn. We found that laser-induced CNV provokes a rapid release of systemic catecholamines, significantly elevated plasma levels of adrenaline and noradrenaline 6 h after injury (Fig. EV4A,B). However, levels of adrenaline and noradrenaline returned to baseline 3- or 7-days post CNV (Fig. EV4C–F). These data suggest that a rise in systemic catecholamines post-laser burn is transient and precedes increases in browning markers in iWAT.

### Inhibition of pathways involved in adipose tissue browning mitigates laser-induced CNV

**Figure EV5:**
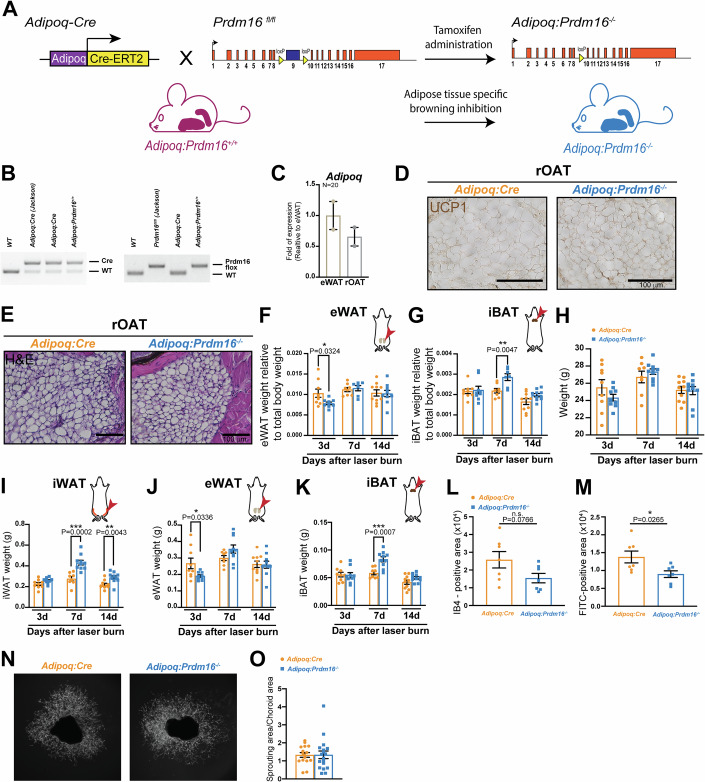
Knockout of *Prdm16* is specific to AT in *Adipoq:Prdm16*^*+/+*^ mice. (**A**) Schematic representation of *Adipoq:Prdm16*^*+/+*^ mice generation. *Adipoq:Cre* mice expressing Cre-ERT2 specifically in the ATs were crossed with *Prdm16*^*lox/lox*^ mice that have *loxP-*flanked exon 9 of *Prdm16*, thus generating *Adipoq:Prdm16*^*+/+*^ mice. Following tamoxifen administration, the exon 9 of *Prdm16* is removed generating *Adipoq:Prdm16*^*−/−*^ mice that have a specific browning inhibition in the ATs from the *Adipoq:Prdm16*^*−/−*^ mice. (**B**) Genotyping of *Adipoq:Cre* and *Adipoq:Prdm16*^*+/+*^ mice for *Cre* and *Prdm16 loxP*-flanked regions. (**C**) eWAT and rOAT relative mRNA expression of *Adipoq* from C57BL/6J mice. Gene expression was normalized to eWAT expression, *n* = 2–20 mice per group (as per graph). (**D**, **E**) Representative immunohistochemistry staining images for (**D**) UCP1 and (**E**) H&E in rOAT from gavaged *Adipoq:Cre* and *Adipoq:Prdm16*^*+/+*^ mice, *n* = 4 mice per group. (**F**) eWAT and (**G**) iBAT masses were calculated as a ratio from tissue-specific weight over total body weight from *Adipoq:Cre* and *Adipoq:Prdm16*^*+/+*^ mice at 3, 7, and 14 days after laser burn (*n* = 8–10 mice per group). (**H**) Comparison of body weight from *Adipoq:Cre* and *Adipoq:Prdm16*^*+/+*^ mice, at 3, 7, and 14 days after laser burn (*n* = 8–10 mice per group). (**I**) iWAT, (**J**) eWAT, and (**K**) iBAT masses of *Adipoq:Cre* and *Adipoq:Prdm16*^*+/+*^ mice at 3, 7, and 14 days after laser burn (*n* = 8–10 mice per group). (**L**) Quantification of isolectin B_4_-positive area and (**M**) quantification of FITC–dextran-labeled CNV area per laser burned *Adipoq:Cre* and *Adipoq:Prdm16*^*+/+*^ mouse 14 days after laser burn; *n* = 7 mice per group. (**N**) Representative images of choroid explants and (**O**) quantification of sprouting area over choroid explant area from *Adipoq:Cre* (*n* = 16 explants) and *Adipoq:Prdm16*^*+/+*^ (*n* = 18 explants) mice gavaged with tamoxifen. Data were normalized to *Adipoq:Cre* mice choroid explants. Data are presented as mean ± SEM. Statistical significance was assessed using unpaired two-tailed Student’s *t* test. Exact *P* values are indicated in the figure, with *P < 0.05, **P < 0.01, and ***P < 0.001. Source data are available online for this figure.

**Figure 3:**
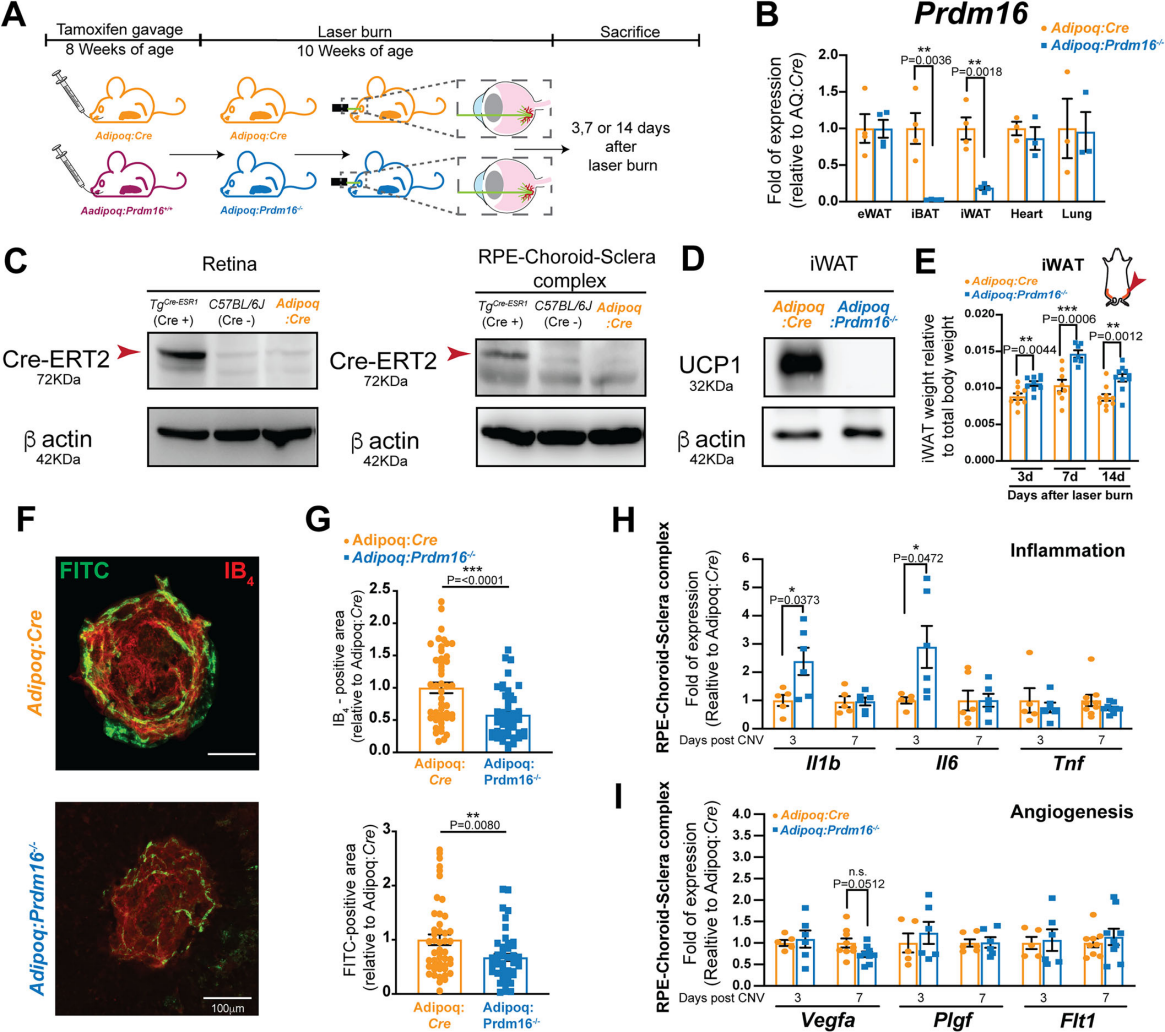
Inhibition of pathways involved in adipose tissue browning mitigates laser-induced CNV. (**A**) Schematic representation of the experimental design of knockout model. At 8 weeks of age, *Adipoq:Cre* and *Adipoq:Prdm16*^*+/+*^ mice were gavaged with tamoxifen for four consecutive days. At 10 weeks of age, the *Adipoq:Cre* and *Adipoq:Prdm16*^*+/+*^ mice were subjected to 4 laser burns per eye (400 mW, 50 µm, 0.05 s) with an argon laser to induce CNV. 3, 7, or 14 days after laser burn, the mice were euthanized, and organs were collected for analysis. (**B**) Relative mRNA expression of *Prdm16* from gavaged *Adipoq:Cre* and *Adipoq:Prdm16*^*+/+*^ mice in eWAT, iBAT, iWAT, heart and lung. mRNA levels were normalized to *Tbp* expression, and gene expression was normalized to *Adipoq:Cre* mice, *n* = 4 mice per group. (**C**) Representative immunoblots for retina and RPE-Choroid-Sclera complex levels of Cre-ERT2 from C57BL/6 J (negative control), *Tg*^*Cre-ESR1*^ (positive control), gavaged *Adipoq:Cre* and gavaged *Adipoq:Prdm16*^*+/+*^ mice, *n* = 2–4 mice per group. β-actin was used as a loading control. (**D**) Representative immunoblots for iWAT levels of UCP1 from gavaged *Adipoq:Cre* and *Adipoq:Prdm16*^*+/+*^ mice, *n* = 3 mice per group. β-actin was used as a loading control. (**E**) iWAT masses were calculated as a ratio from tissue-specific weight over total body weight from *Adipoq:Cre* and *Adipoq:Prdm16*^*+/+*^ mice at 3, 7, and 14 days after laser burn (*n* = 8 mice per group). (**F**) Representative images of choroidal flat mounts from *Adipoq:Cre* and *Adipoq:Prdm16*^*+/+*^ mice stained with FITC–dextran-labeled CNV (FITC) and isolectin B_4_ (IB_4_), imaged on a confocal microscope at ×30 magnification; scale bar 100 µm. (**G**) Quantification of isolectin B_4_-positive area and quantification of FITC–dextran-labeled CNV area relative to *Adipoq:Cre* mice 14 days after laser burn; *n* = 47 burns *Adipoq:Cre* (*n* = 47 burns) and *Adipoq:Prdm16*^*+/+*^(*n* = 46 burns). (**H**, **I**) Relative mRNA expression of *Il1b*, *Il6* and *Tnf* related to inflammation (**H**) and *Vegfa, Plgf and Flt1* related to angiogenesis (**I**) from laser burned *Adipoq:Cre* and *Adipoq:Prdm16*^*+/+*^ mice in RPE-Choroid-Sclera complex at 3- and 7-days post laser burn, *n* = 6–9 mice per group. mRNA levels were normalized to *Actb* expression and gene expression was normalized to *Adipoq:Cre* mice. Data are presented as mean ± SEM. Statistical significance was assessed using unpaired two-tailed Student’s *t* test. Exact *P* values are indicated in the figure, with *P < 0.05, **P < 0.01, and ***P < 0.001. Source data are available online for this figure.

Given evidence for activation of pathways linked to AT browning in iWAT during CNV (Fig. 1), we sought to determine its role in pathological CNV. To interfere with AT browning, we generated an inducible knockout (KO) mouse deficient in pathways of browning by crossing Adiponectin-CreERT2 (*Adipoq:Cre*) with PR/SET Domain 16 (PRDM16)-floxed mice (*Adipoq:Prdm16)*. In this model, the Cre recombinase is coupled to the estrogen receptor 2 (CreERT2) and its expression is localized specifically to adipocytes. PRDM16 is a powerful transcriptional coregulator and driver of beige adipocyte identity (Cohen et al, 2014; Harms and Seale, 2013; Seale et al, 2008; Seale et al, 2011; Seale et al, 2007; Wang et al, 2019) that can induce expression of thermogenic genes such as *Ucp1* and its deletion causes loss of beige adipocytes (Seale et al, 2011). Following tamoxifen administration, CreERT2 is translocated from the cytoplasm to the nucleus, leading to deletion of the *loxP*-flanked exon 9 of *Prdm16*, the exon responsible for PRDM16 induction of AT browning (Fig. EV5A) (Cohen et al, 2014). The presence of adiponectin promoter and *Prdm16 loxP*-flanked regions in these mice was verified by PCR genotyping (Fig. EV5B). At 8 weeks of age, *Adipoq:Cre* or *Adipoq:Prdm16*^*+/+*^ mice were gavaged with tamoxifen for 4 consecutive days (Fig. 3A). Adipocyte-specific PRDM16-deficient mice (*Adipoq:Prdm16*^*−/−*^) mice showed a selective decrease of *Prdm16* mRNA expression in iBAT and iWAT, whereas the expression in eWAT, heart and lung remained unaffected (Fig. 3B). Cre-ERT2 expression was not detected in retina nor RPE-choroid-sclera complexes of *Adipoq:Cre* mice (Fig. 3C). Functional validation of tissue-specific KO was confirmed through loss of UCP1 protein expression in iWAT of *Adipoq:Prdm16*^*−/−*^ mice (Fig. 3D). Adiponectin (*Adipoq*) was also found in rOAT by qPCR in C57Bl/6J (Fig. EV5C). Furthermore, histological assessment of *Adipoq:Prdm16*^*−/−*^ rOAT did not show differences in the expression of UCP1, nor morphological changes compared to *Adipoq:Cre* mice, as seen by H&E staining (Fig. EV5D,E). Altogether, these data suggest that rOAT in our KO mice is not affected by the KO of the exon 9 of *Prdm16*.

Two weeks post tamoxifen treatment, *Adipoq:Prdm16*^*−/−*^ and control *Adipoq:Cre* mice were subjected to laser-induced CNV. Gross impact on adiposity was assessed by weighing AT depots (normalized to total body weight) at 3, 7, and 14 days after laser. The most notable changes were observed in iWAT, where *Adipoq:Prdm16*^*−/−*^ mice presented a higher adiposity than *Adipoq:Cre* at all assessed time-points (Figs. 3E and  EV5F,G). Of note, *Adipoq:Cre* and *Adipoq:Prdm16*^*−/−*^ mice did not show any differences in their total body weight post CNV (Fig. EV5H). Differences observed in iWAT, eWAT, and iBAT weights were also detected independently of total body weight normalization (Fig. EV5I–K). Two weeks after laser CNV induction, animals were perfused with fluorescein isothiocyanate (FITC)-Dextran to evaluate perfusable neovessels. RPE-choroid-sclera complex were dissected and labeled with isolectin B_4_ (IB_4_) to assess neovascular lesion size (Fig. 3F). Quantification of IB_4_ and FITC–dextran vessels showed a significant decrease in CNV at 14 days in *Adipoq:Prdm16*^*−/−*^ mice compared to *Adipoq:Cre* mice (Fig. 3G). Averages of IB_4_-positive and FITC-positive CNV areas per mouse were reduced in *Adipoq:Prdm16*^*−/−*^ mice (Fig. EV5L,M). Since tamoxifen has been shown to affect angiogenesis in the retina (Brash et al, 2020), we investigated whether tamoxifen itself had an inhibitory effect in our model. Two weeks after tamoxifen treatment, choroidal explants from *Adipoq:Cre* and *Adipoq:Prdm16*^*−/−*^ revealed similar vascular growth in both genotypes (Fig. EV5N,O), suggesting that tamoxifen did not influence angiogenesis in our experimental paradigm and highlighting a non-vascular role for *Prdm16* in Adiponectin-expressing cells. We next investigated the expression of inflammatory mediators associated with para-inflammation and neovascularization in AMD (Agarwal et al, 2018). Three days post laser-CNV, we observed a significant upregulation of *Il1b* and *Il6* transcripts in the RPE-choroid-sclera complex from *Adipoq:Prdm16*^*−/−*^ mice (Fig. 3H). Interestingly, at 7 days post CNV, we observed a trend toward decreased *Vegfa* expression in RPE-choroid-sclera complex from *Adipoq:Prdm16*^*−/−*^ mice compared to control Adipoq:Cre mice (Fig. 3I). While inflammatory cytokines recruit mononuclear phagocytes (MNPs), data from other groups and our own have shown that inflammatory MNPs (simplistically M1) can reduce, limit, or prune CNV (Andriessen et al, 2021; Zandi et al, 2015). Altogether, these data suggest that PRDM16-mediated signaling pathways involved in adipose tissue browning can influence the development of CNV.

### PRDM16 expression in adipocytes promotes CNV and choroidal vascular sprouting

**Figure 4:**
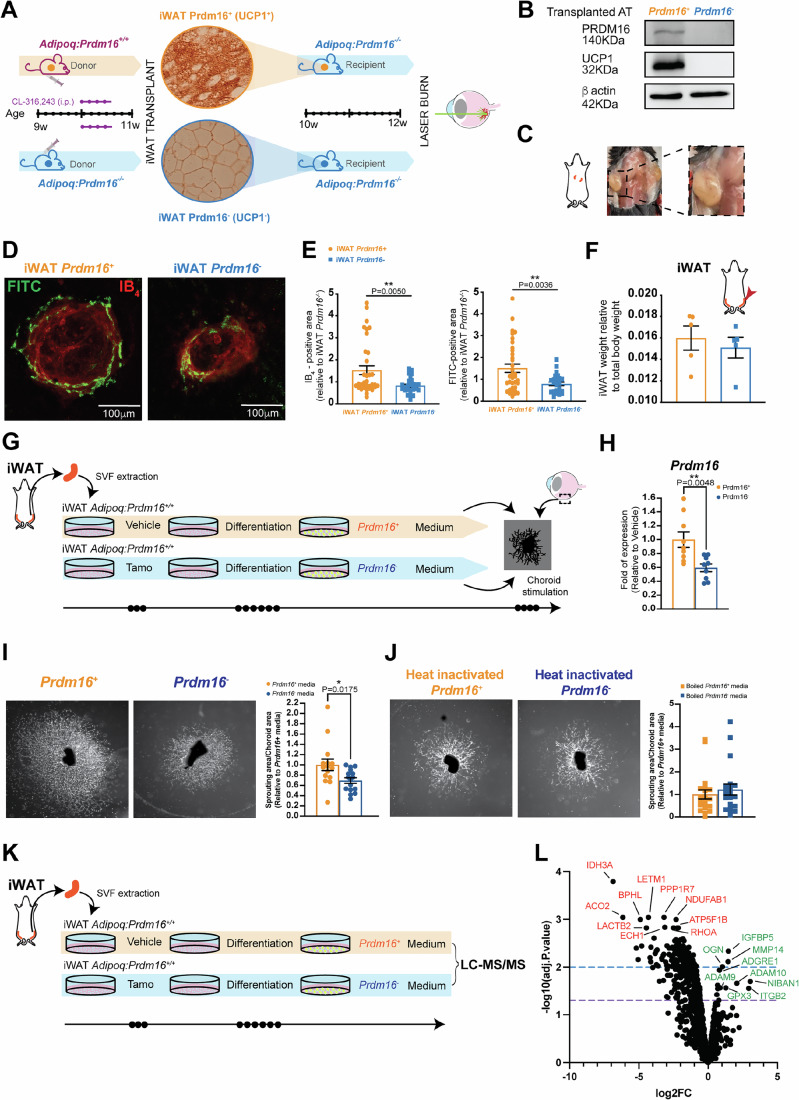
PRDM16 expression in adipocytes promotes CNV and choroidal vascular sprouting. (**A**) Schematic representation of ATT experimental design. At 8 weeks of age, half of donor *Adipoq:Prdm16*^*+/+*^ mice were gavaged with tamoxifen and the other half were gavaged with corn oil (vehicle) for four consecutive days. At 10 weeks of age, donor *Adipoq:Prdm16*^*+/+*^ and *Adipoq:Prdm16*^*−/−*^ mice were injected I.P. with CL316,243 daily for 5 consecutive days and their iWAT was collected for ATT. In parallel, 8-week-old recipient *Adipoq:Prdm16*^*+/+*^ mice were gavaged with tamoxifen for 4 consecutive days. At 10 weeks of age, recipient *Adipoq:Prdm16*^*−/−*^ mice were transplanted with 200 mg of *Prdm16*^+^ iWAT or *Prdm16*^*-*^ iWAT from donor mice. Three weeks after ATT, mice were subjected to 4 laser burns per eye (400 mW, 50 µm, 0.05 s) with an argon laser to induce CNV. Fourteen days after laser burn, mice were euthanized and organs were collected for analysis. (**B**) Representative immunoblots of PRDM16 and UCP1 from transplanted AT of *Adipoq:Prdm16*^*+/+*^ and *Adipoq:Prdm16*^*−/−*^ donor mice treated with CL316,243. β-actin was used as loading control. (**C**) Representative photographs of transplanted AT of *Adipoq:Prdm16*^*+/+*^ mice 14 days after laser. (**D**) Representative images of choroidal flat mounts from *Adipoq:Prdm16*^*+/+*^ mice transplanted with *Prdm16*-positive or *Prdm16*-negative iWAT stained with FITC–dextran-labeled CNV and isolectin B_4_, imaged on a confocal microscope at 30X magnification; scale bar 100 µm. (**E**) Quantification of isolectin B_4_-stained positive area and quantification of FITC–dextran-labeled CNV from *Adipoq:Prdm16*^*+/+*^ mice transplanted with *Prdm16*-positive or *Prdm16*-negative iWAT. Data were normalized to *Adipoq:Prdm16*^*+/+*^ mice transplanted with *Prdm16*-positive iWAT 14 days after laser burn; *Prdm16*-positive iWAT, *n* = 26 burns, and *Prdm16*-negative iWAT, *n* = 35 burns. (**F**) iWAT masses were calculated as a ratio from tissue-specific weight over total body weight from *Adipoq:Prdm16*^*+/+*^ mice transplanted with *Prdm16*-positive or *Prdm16*-negative iWAT at 14 days after laser burn (*n* = 5 mice per group). (**G**) Schematic representation of the primary adipocyte culture experimental design. SVF was extracted from iWAT of *Adipoq:Prdm16*^*+/+*^ mice, and primary preadipocytes were isolated and placed in cell culture. Primary preadipocytes were treated with 4-hydroxy-tamoxifen (Tamo) or Ethanol (Vehicle) for 3 consecutive days once daily and differentiated into mature adipocytes. Six days after differentiation, the mature adipocytes were changed to basal DMEM, and the conditioned medium was collected after 48 h. Choroid explants were extracted from C57BL/6J mice and treated during 4 days with the conditioned medium of *Prdm16*-positive or *Prdm16*-negative adipocytes. (**H**) Relative mRNA expression of *Prdm16* from *Prdm16*-positive or *Prdm16*-negative adipocytes. *Tbp* was used as an internal control, and gene expression was normalized to Prdm16-positive adipocytes, *n* = 9 per group. (**I**) Representative images of choroid explants and quantification of sprouting area over choroid area 4 days after stimulation with the conditioned medium of *Prdm16*-positive and *Prdm16*-negative adipocytes, *n* = 12 explants per group. Data were normalized to choroid explants treated with *Prdm16*-positive adipocytes. (**J**) Representative images of choroid explants and quantification of sprouting area over choroid area 4 days after stimulation with the conditioned medium of *Prdm16*-positive or *Prdm16*-negative adipocytes boiled at 95 °C for 5 min, *n* = 12 explants per group. Data were normalized to choroid explants treated with conditioned medium from *Prdm16*-positive adipocytes boiled at 95 °C for 5 min. (**K**) Schematic representation of primary adipocyte culture experimental design for subsequent LC-MS/MS analysis. (**L**) Volcano plot of log2 fold change and −log10 adjusted *P* value of *Prdm16*-positive adipocyte secretome relative to *Prmd16*-negative. Green depicts the most enriched proteins in the *Prdm16*-positive adipocyte secretome, and red depicts the most downregulated proteins. Data are presented as mean ± SEM. Statistical significance was assessed using unpaired two-tailed Student’s *t* test. Exact *P* values are indicated in the figure, with *P < 0.05 and **P < 0.01. Source data are available online for this figure.

**Figure EV6:**
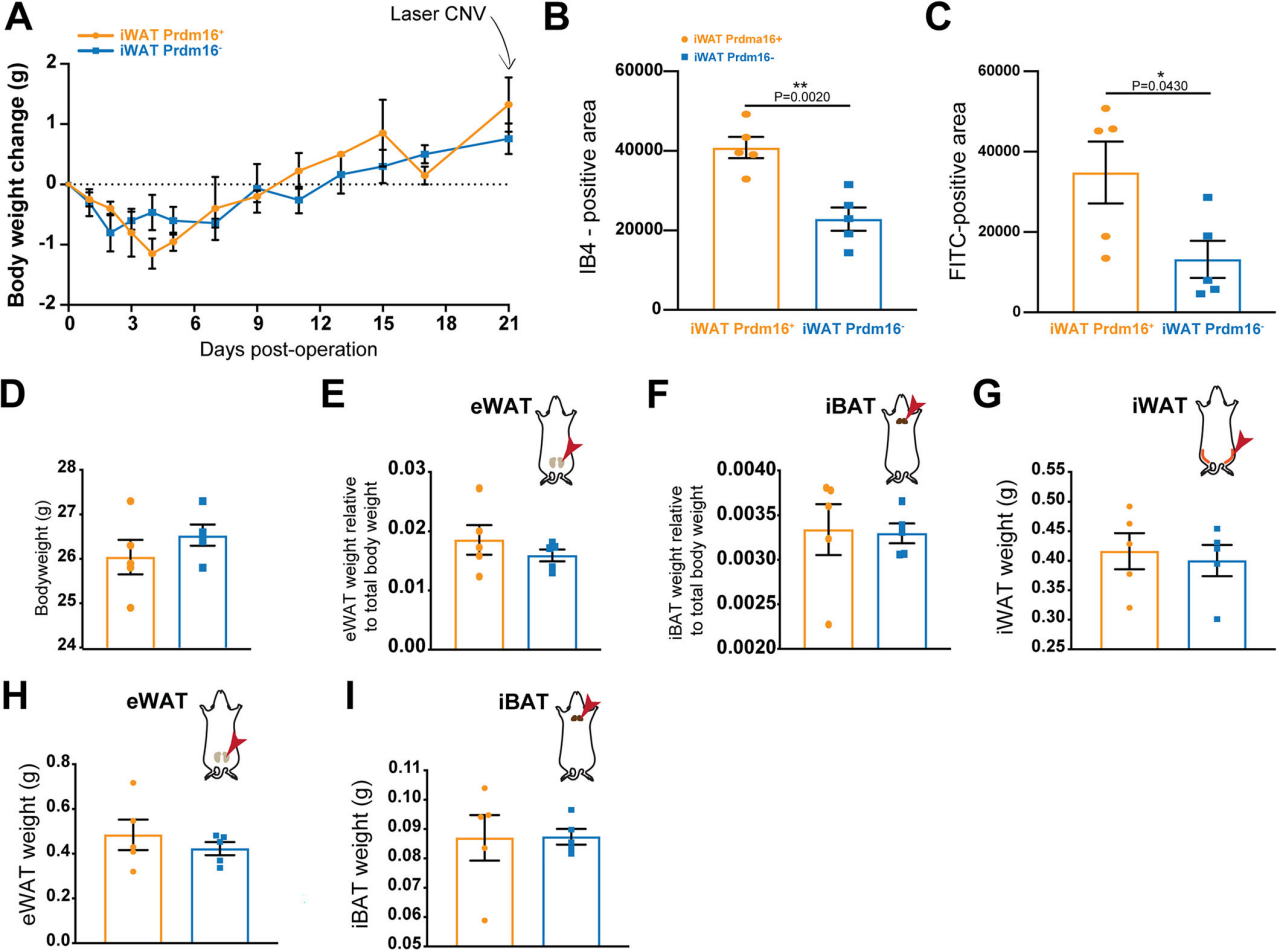
Changes in AT weight in *Adipoq:Prdm16*^*−/−*^ mice following ATT. (**A**) Body weight change from *Adipoq:Prdm16*^*−/−*^ recipient mice transplanted with either *Prdm16*-positive or *Prdm16*-negative iWAT for 21 days following the ATT (*n*  =  5). (**B**) Quantification of isolectin B4-positive area and (**C**) quantification of FITC–dextran-labeled CNV area per *Adipoq:Prdm16*^*+/+*^ mice transplanted with *Prdm16*-positive or *Prdm16*-negative iWAT 14 days after laser burn; *n* = 5 mice per group. (**D**) Comparison of the body weight of *Adipoq:Prdm16*^*+/+*^ transplanted mice with either *Prdm16*-positive or *Prdm16*-negative iWAT. (**E**) eWAT and (**F**) iBAT masses were calculated as a ratio from tissue-specific weight over total body weight from *Adipoq:Prdm16*^*+/+*^ mice transplanted with *Prdm16*-positive or *Prdm16*-negative iWAT at 14 days after laser burn (*n* = 5 mice per group). (**G**) iWAT, (**H**) eWAT, and (**I**) iBAT masses of *Adipoq:Prdm16*^*+/+*^ transplanted mice with either *Prdm16*-positive or *Prdm16*-negative iWAT at 14 days after laser burn (*n* = 5 mice per group). Data are presented as mean ± SEM. Statistical significance was assessed using unpaired two-tailed Student’s *t* test. Exact *P* values are indicated in the figure, with *P < 0.05 and **P < 0.01. Source data are available online for this figure.

To confirm that PRDM16 in adipocytes contributed to CNV, we next reintroduced active BgAT through heterologous adipose tissue transplantation (ATT). We engrafted 200 mg of iWAT from either *Adipoq:Prdm16*^*−/−*^ or *Adipoq:Prdm16*^*+/+*^ donor mice into *Adipoq:Prdm16*^*−/−*^ recipient mice (Fig. 4A). To trigger browning in iWAT, donor mice were pre-treated with intraperitoneal injections of CL316,243 for 5 consecutive days. Induction of browning in donor iWAT was confirmed histologically by robust expression of UCP1-positive multilocular adipocytes as well as an increase in PRDM16 and UCP1 protein expression in *Adipoq:Prdm16*^*+/+*^. As expected, AT from *Adipoq:Prdm16*^*−/−*^ mice did not show signs of browning (Fig. 4A,B). Following ATT, mice were allowed to recover for 3 weeks. No significant differences in weight were observed between groups (Fig. EV6A). Three weeks after ATT, *Adipoq:Prdm16*^*−/−*^ recipient mice were subjected to laser-induced CNV. ATT were monitored for tissue revascularization to confirm the success of the procedure and ensure grafts were non-necrotic (Fig. 4C). Recipient mice transplanted with PRDM16-positive iWAT showed an increase in CNV compared to mice transplanted with PRDM16-deficient iWAT, with overall increase in IB_4_-positive as well as in FITC-positive areas (Fig. 4D,E). Relative IB_4_-positive and CNV areas per mouse confirmed increases in both parameters (Fig. EV6B,C). Importantly, mouse weight was not altered by ATT (Fig. EV6D), and adiposity (normalized AT depot weight to whole-body weight) did not vary between mice that received transfers from either *Adipoq:Prdm16*^*−/−*^ or *Adipoq:Prdm16*^*+/+*^ mice (Figs. 4F and EV6E–I). These data confirm that AT with the capacity to initiate browning exacerbates neovascularization in the eye and underscore the potential role of AT browning in neovascular retinal disease.

Based on the above, we investigated whether secreted factors from AT undergoing browning could influence angiogenesis. We established a cell culture model of preadipocytes containing the stromal vascular fraction (SVF) of iWAT from *Adipoq:Prdm16*^*+/+*^ mice treated with either 4-hydroxy-tamoxifen or vehicle (control). Mice were not subjected to laser CNV in order to decouple angiogenesis that may result from distal burns, which have been shown to induce AT browning (Patsouris et al, 2015; Sidossis et al, 2015). Eight days after differentiation, adipocytes were placed in serum-free media, and the media collected 48 h later (Fig. 4G). Efficacy of the knockout was verified with decreased expression of *Prdm16* in adipocytes after treatment with 4-hydroxy-tamoxifen (Fig. 4H). Choroid explants from C57Bl/6 J mice were cultured with serum-free adipocyte-conditioned media collected from *Prdm16*-positive or *Prdm16*-negative adipocytes. Evaluation of sprouting area per choroid in explants after 4 days showed that choroids exposed to conditioned media from *Prdm16*-positive adipocytes had consistently greater sprouting than those treated with medium from *Prdm16*-negative adipocytes (Fig. 4I). The reduction in sprouting area was proportional to *Prdm16* knockdown, suggesting that *Prdm16*-expressing adipocytes secrete factors that contribute to choroidal angiogenesis. To corroborate if *Prdm16*-positive adipocytes stimulate choroidal explant sprouting through secreted factors (cytokines, adipokines, lipids, complement factors, nucleic acids, or other proteins), we denatured the conditioned media through boiling at 95 °C. Heat-inactivation blunted the increased sprouting observed with *Adipoq:Prdm16*^*+/+*^ adipocytes (Fig. 4J). Altogether, these findings suggest that factors secreted by PRDM16-expressing adipocytes have the propensity to amplify choroidal angiogenesis.

To identify adipose-derived PRMD16-dependent effectors modulating choroidal neovascularization, cell culture medium from differentiated *Prdm16*-positive and *Prdm16*-negative adipocytes underwent LC-MS/MS (Fig. 4K). Reactome proteomics analysis (Griss et al, 2020) revealed a total of 340 proteins were differentially regulated in the *Prdm16*-positive adipocyte culture media compared to that of *Prdm16*-negative adipocytes, the majority of which were significantly reduced (Fig. 4L). All top 10 upregulated (Table 1, and depicted in green in Fig. 4L) and top 10 downregulated proteins (Table 2, and depicted in red in Fig. 4L) were validated in ExoCarta, a compendium of exosomal cargo (Keerthikumar et al, 2016), and/or Vesiclepedia, an extracellular vesicle and extracellular particle repository (Chitti et al, 2024). Of the secreted proteins most enriched in *Prdm16*-positive adipocytes, 70% (Insulin-Like Growth Factor-Binding Protein 5 (IGFBP5), Matrix Metalloproteinase 14 (MMP14), Osteoglycin (OGN), Disintegrin and Metalloproteinase 10 (ADAM10), ADAM9, Glutathione Peroxidase-3 (GPX3), and Integrin Subunit Beta 2 (ITGB2)) have been implicated in retinal neovascularization, including neovascular AMD (Table 1). The reduction of these proteins in the *Prdm16*-negative adipocyte secretome could provide a mechanistic link to the significantly higher levels of CNV and vascular sprouting observed in *Adipoq:Prdm16*^+/+^ mice compared to *Adipoq:Prdm16*^−/−^ mice.

**Table 1:** Top 10 enriched proteins in *Prdm16*-positive adipocyte secretomes.

Identifier	Log2FC	Avg Expr	t-statistic	*P* value	Adj.*P* value	B-statistic
IGFBP5	1.4639683	0.7192522	7.579991	0.0002266	0.0046101	1.0620316
MMP14	1.4411157	0.6947084	6.177267	0.0007115	0.0076312	−0.1808943
OGN	1.0274961	0.5062909	5.417723	0.0014403	0.0098354	−0.9519005
ADGRE1	0.8196358	0.4084402	5.124444	0.0019269	0.0115038	−1.2705135
NIBAN1	3.0412803	1.1467879	4.326014	0.0045150	0.0198303	−2.2014991
ADAM10	2.0714547	0.7864075	4.227295	0.0050483	0.0216946	−2.3232589
OLFML2B	0.6853603	0.3425902	4.116171	0.0057343	0.0238213	−2.4620849
ADAM9	0.8687415	0.4117955	3.989612	0.0066449	0.0269309	−2.6224609
GPX3	1.2809003	0.6192760	3.978303	0.0067338	0.0270711	−2.6369078
ITGB2	2.9268392	0.9735907	3.951650	0.0069486	0.0277110	−2.6710326

Log2 fold change, average expression, t-statistic, *P* value, adjusted *P* value, and B-statistic of the log-odds of differential expression of the top 10 enriched proteins in *Prdm16*-positive adipocyte secretome.

**Table 2:** Top 10 decreased proteins in *Prmd16*-positive adipocyte secretomes.

Identifier	Log2FC	Avg Expr	t-statistic	*P* value	Adj.*P* value	B-statistic
IDH3A	−6.864915	−3.483098	−25.19462	1.62e-07	0.0001618	7.897952
PPP1R7	−3.186751	−1.611273	−15.44389	3.30e-06	0.0009068	5.385823
ACO2	−6.154128	−3.122420	−15.21272	3.61e-06	0.0009068	5.299474
LETM1	−4.308805	−2.170667	−15.19598	3.64e-06	0.0009068	5.293153
BPHL	−4.891873	−2.466830	−14.11763	5.69e-06	0.0010041	4.866000
NDUFAB1	−2.312339	−1.161921	−13.98936	6.04e-06	0.0010041	4.808645
ECH1	−3.126846	−1.575664	−12.81147	1.03e-05	0.0014606	4.290218
ATP5F1B	−2.497989	−1.253429	−12.39498	1.25e-05	0.0015062	4.091497
LACTB2	−4.461894	−2.235323	−12.20665	1.37e-05	0.0015062	3.999029
RHOA	−2.161395	−1.085067	−11.85575	1.64e-05	0.0015062	3.822222

Log2 fold change, average expression, t-statistic, *P* value, adjusted *P* value, and B-statistic of the log-odds of differential expression of the top 10 decreased proteins in *Prdm16*-positive adipocyte secretome.

Seven of the top ten proteins with lower expression in *Prdm16*-positive adipocyte secretomes were mitochondrial (IDH3A, ACO2, LETM1, NDUFAB1, ECH1, ATP5F1B, LACTB2) (Table 2). These proteins likely originate from small extracellular vesicles released in the culture media, as all were validated exosomal cargo proteins. Collectively, these data suggest that PRDM16 expression in adipocytes is associated with expression of proteins with roles in neovascular AMD and further solidify the link between adipose PRDM16 and disease progression.

## Discussion

Aging and obesity are both linked to the development of age-related diseases such as AMD and induce a redistribution of AT that increases WAT mass while decreasing BAT mass (Adams et al, 2011; Cheng et al, 2021; Haas et al, 2015; Zoico et al, 2019). Despite the potential beneficial role of increased BAT and BgAT activity in obesity, the role of thermogenic active AT in age-related diseases remains poorly understood (Darcy & Tseng, 2019; Rui, 2017; Tayanloo-Beik et al, 2023). In our study, we show that laser-induced CNV causes a selective remodeling of distal iWAT by potentially increasing the content of beige-like adipocytes. We provide evidence that deletion of PRDM16 specifically in AT decreases CNV, whereas reintroduction of PRDM16-positive adipose tissue increases CNV. Moreover, PRDM16 expression in adipocytes is sufficient to potentiate neovascularization in choroidal explants in vitro via soluble factors that could include IGFBP5, MMP14, OGN, Disintegrin, ADAM9 & 10, GPX3, and ITGB2. These findings suggest that PRDM16 expression in adipocytes may influence the progression of neovascular AMD and potentially other neovascular ocular diseases such as proliferative diabetic retinopathy.

Inter-organ communication via endocrine, immune, or other systems is essential for homeostasis (Yuan et al, 2021). Distal organs influence the retina through several axes. For example, the liver can influence eye health via the secretion of hepatokines (e.g., FGF21, HGF, CFH, PEDF) into the bloodstream and hence dysregulation of liver metabolism may impact retinal function (Fu et al, 2017; Grierson et al, 2000; Klein et al, 2005; Wang et al, 2021; Ye et al, 2022). Moreover, the gut-eye axis has been associated with ocular diseases such as diabetic retinopathy and AMD (Floyd & Grant, 2020). High-fat diets alter gut microbiota by shifting populations towards Firmicutes at the detriment of Bacteriodetes, along with increasing gut–vascular permeability and systemic inflammation that exacerbates CNV (Andriessen et al, 2016). These findings are in line with the observation that patients with higher levels of Firmicutes and lower levels of Bacteriodetes show increased incidence of CNV (Zinkernagel et al, 2017), with gut Bacteriodetes associated with a protective effect (Rowan et al, 2017). Recent studies described an axis that implicates regulation of retinal homeostasis by the AT (Hata et al, 2023; Meng et al, 2023; Sterling et al, 2022). We demonstrated that obesity (even transient obesity) leads to epigenetic reprogramming of the innate immune system and AT transfer of eWAT from formerly obese mice into normal weight healthy mice trigger increased development of CNV compared to mice transplanted with non-obese eWAT (Hata et al, 2023). Furthermore, IL-1β secretion by inflammatory WAT can activate the cellular iron sequestration response (CISR) and lead to RPE dysfunction and death in a dry AMD (Sterling et al, 2022). BAT has also been shown to regulate glucose tolerance in response to light by inhibiting non-shivering thermogenesis via a retina-brain-BAT axis (Meng et al, 2023). The findings of the current study add to the concept of distal adipose tissue contributing to retinal health and suggest that the state of AT (WAT or BgAT) influences CNV, and that retinal neovascular lesions themselves influence AT state. We therefore describe an AT-retina axis that implicates pathways of AT browning and AT-resident PRDM16 activation in CNV formation. Potential adipose-derived factors secreted during AT browning known to influence angiogenesis (Leung et al, 1989; Ouchi et al, 2004) include VEGF and adiponectin (Wang et al, 2015).

Given that obesity and increased visceral AT predispose men to AMD (Adams et al, 2011; Haas et al, 2015), and high-fat diets have deleterious effects on CNV in mice (Andriessen et al, 2016; Sene et al, 2013), we surmised that a reduction of WAT via browning would ameliorate CNV. However, our findings paradoxically suggest the exact opposite, with increased AT browning aggravating CNV. Yet, consistent with our data, AT browning has been shown to have detrimental effects in acute and chronic hypermetabolic conditions that can lead to cachexia, atherosclerosis, hepatic steatosis, and muscle wasting (Das et al, 2011; Dong et al, 2013; Jeschke et al, 2011; Petruzzelli et al, 2014). Moreover, PRDM16 is a key regulator of BAT differentiation, and we observed that loss of PRDM16 in adipocytes led to a reduction in proteins associated with pathological retinal neovascularization in patients with wet AMD, PDR or in animal models such as IGFBP5, MMP14, OGN, Disintegrin and ADAM9 & 10 GPX3, and ITGB2 (Abu El-Asrar et al, 2018; Bisen et al, 2025; Coronado et al, 2021; Guaiquil et al, 2009; Lofqvist et al, 2009; Na et al, 2023; Sakurai et al, 2003; Strzalka-Mrozik et al, 2017).

Most of the top ten proteins with lower expression in *Prdm16*-positive adipocyte secretomes were mitochondrial and likely traveled in extracellular vesicles. Given that secretomes can provide insight into the metabolic state of their parental cell (Rohm et al, 2025), these data may suggest that mitochondrial metabolism is differentially regulated in *Prdm16*-positive adipocytes compared to *Prdm16*-negative adipocytes. Of the top 10 proteins with reduced expression in *Prdm16*-positive adipocyte secretomes, Protein Phosphatase 1 Regulatory Subunit 7 (PPP1R7) negatively regulates the AKT kinase signaling pathway and abrogates cell migration (Paul et al, 2019), and Ras Homolog family member A (RHOA) inhibits endothelial cell proliferation, migration, tube formation, and angiogenic sprouting in vitro (Hauke et al, 2022). The reduced expression of these two proteins in the *Prdm16*-positive adipocyte secretome may contribute to explaining the significantly higher levels of CNV and choroidal sprouting observed in *Adipoq:Prdm16*^+/+^ mice when compared to *Adipoq:Prdm16*^−/−^ mice.

Furthermore, several studies have shown that patients with increased AT mass are protected against chronic diseases, a phenomenon known as the obesity paradox (Doehner et al, 2012; Gruberg et al, 2002; Hainer & Aldhoon-Hainerova, 2013; Lainscak et al, 2012). Further investigation of the state of AT (WAT or BgAT) in patients living with chronic age-related diseases such as AMD may provide deeper insight on the role of obesity and AT metabolism in health and disease.

In summary, we provide evidence for an axis between the retina and adipose tissue where during CNV, iWAT undergoes tissue remodeling that ultimately influences the original lesion in the eye. Through PRDM16 and downstream effectors, visceral AT contributes to pathological neovascularization in an experimental model of neovascular AMD. Ultimately, our study underscores the importance of the state of AT (WAT versus BgAT) in influencing the progression of CNS diseases such as AMD. In a more comprehensive manner, this study highlights the existence of reciprocal communication between the retina and distal tissues, such as AT, and their potential role in ocular health and disease.

## Methods

**Table:** Reagents and tools table

Reagent/resource	Reference or source	Identifier or catalog number
**Experimental models**		
C57BL/6 J (*M. Musculus*)	Jackson Lab	000664
*PRDM16*^*lox/lox*^ (*M. Musculus*)	Jackson Lab	024992
*Adipo:Cre* (*M. Musculus*)	Jackson Lab	025124
*Adipoq:Prdm16*	In-house breeding	
**Antibodies**		
Rabbit anti-UCP1	ThermoFisher	PAI-24894
Rabbit anti-PPARGC1a	Abcam	Ab54481
Mouse anti-β-actin	Cell Signaling	3700
Rabbit anti-CRE recombinase	Abcam	Ab216262
Rabbit anti-PRDM16	Abcam	Ab106410
Rat anti-mouse CD16/32	Biolegend	101330
Rat anti-mouse/human CD11b, BV711	Biolegend	101242
Rat anti anti-mouse F4/80, PE	Biolegend	123110
Mouse anti-mouse CD64, APC	Biolegend	139305
Rat anti-mouse CD38, FITC	Biolegend	102705
Rat anti-mouse Ly-6G, APC/Cy7	Biolegend	127624
Armenian Hamster anti-mouse CD11c, BV785	Biolegend	117335
**Oligonucleotides and other sequence-based reagents**		
*Prdm16* primers	This study	Forward CAG CAC GGT GAA GCC ATT C Reverse GCG TGC ATC CGC TTG TG
*Ucp1* primers	This study	Forward GGC CTC TAC GAC TCA GTC CA Reverse TAA GCC GGC TGA GAT CTT GT
*Ppargc1a* primers	This study	Forward ACG CGT GAC CAC TGA CAA CGA G Reverse GCT GCA TGG TTC TGA GTG CTA AG
*Cidea* primers	This study	Forward AAT AGC CAG AGT CAC CTT CG Reverse AGC AGA TTC CTT AAC ACG GC
*Cox7a1* primers	This study	Forward GCT GAG GAC GCA AAA TGA G Reverse CTG CCA CAC GGT TTT CTA AG
*Il1b* primers	This study	Forward CTG GTA CAT CAG CAC CTC ACA Reverse GAG CTC CTT AAC ATG CCC TG
*Il6* primers	This study	Forward CTT CCA TCC AGT TGC CTT C Reverse ATT TCC ACG ATT TCC CAG AG
*Tnf* primers	This study	Forward CGC GAC GTG GAA CTG GCA GAA Reverse CTT GGT GGT TTG CTA CGA CGT GGG
*Adipoq* primers	This study	Forward AGC CGC TTA TGT GTA TCG CT Reverse GAG TCC CGG AAT GTT GCA GT
*Vegfa* primers	This study	Forward CCC GAC GAG ATA GAG TAC AT Reverse CAG GGC TTC ATC GTT ACA G
*Plgf* primers	This study	Forward CAT ATT CAG TCC GTC CTG TG Reverse TAG TGA TGT TGG CTG TCT TTA T
*Flt1* primers	This study	Forward CTG AAG CGG TCT TCT TCC GA Reverse TCA GTC TCT CCC GTG CAA AC
*Tbp* primers	This study	Forward ACC CTT CAC CAA TGA CTC CTA TG Reverse TGA CTG CAG CAA ATC GCT TGG
*Actb* primers	This study	Forward GAC GGC CAG GTC ATC ACT ATT G Reverse CCA CAG GAT TCC ATA CCC AAG A
**Chemicals and other reagents**		
Tamoxifen	Sigma	C8267
Zombie Aqua	Biolegend	423101
FITC-dextran	Sigma	FD2000S
Rhodamine-labeled *Griffonia (bandeiraea) simplicifolia* Isolectin I	Vector Laboratories	RL-1102
RIPA 1X	Cell Signaling Technologies	9806
Trizol	Life technologies	15596018
5X iScript RT Supermix	BIO-RAD	1708841
iTaq Universal SYBR® Green Supermix	BIO-RAD	1725124
Permount	Fisher Chemical	UN1294
Collagenase Type II	Sigma	C6885
EDTA	MJS Biolynx	UB15694S2
RBC lysis buffer	eBioscience	00-4333-57
CL316,243	Sigma	C5976
DMEM F12	Corning	10-090-CV
DMEM	Corning	10-017-CV
Fetal bovine serum	Multicell	090450
Penicillin/Streptomycin	Corning	30-00-CI
3-isobutyl-1-methylxanthine	Sigma	I5879
dexamethasone	Sigma	D2915
insulin	Sigma	I9278
4-hydroxy-tamoxifen	Sigma	H7904
Matrigel	Corning	356230
EBM-2	Lonza	CC-3156
EGM-2 SingleQuots	Lonza	CC-4176
Hydroxyl PAC beads	ReSyn Biosciences	MR-HYX002
Lys‑C/Trypsin	Promega	V5071
**Software**		
MassHunter Quantitative analysis software	Agilent Technologies	
ImageJ	U. S. National Institutes of Health	
Axio Vision software	Zeiss	
FACSDiva^TM^ Software	BD Biosciences	
FlowJo^TM^ v10.8 Software	BD Life Sciences	
Scaffold Proteome 4.8	Scaffold	
GraphPad Prism 9.0	GraphPad	
**Other**		
HRP/DAB (ABC) Detection IHC kit	Abcam	ab64264

### Animal model

All experimental procedures were approved by the Animal Care Committee of the Centre de Recherche Hôpital Maisonneuve-Rosemont (protocol numbers 2019-1542 and 2023-3180) and were performed in compliance with ethical regulations in accordance with the guidelines of the Canadian Council on Animal Care. C57BL/6J mice (RRID:IMSR_JAX:000664) were originally purchased at Jackson Laboratories and later bred in-house. *PRDM16*^*lox/lox*^ mice (*B6.129-Prdm16 tm1.1Brsp/J*, RRID:IMSR_JAX:024992) homozygous for a *Prdm16* allele with two loxP sites flanking exon 9 and *Adipo:Cre* mice (C*57BL/6-Tg(Adipoq-cre/ERT2)1Soff/J*, RRID:IMSR_JAX:025124) were purchased from The Jackson Laboratory and crossed to generate adipocyte-specific PRDM16 knockout mice (*Adipoq:Prdm16*). Mice were bred and housed at 22 °C with ad libitum access to standard laboratory chow and water under a 12 h light/dark cycle. Only male mice were used in this study. Treatments and groups were assigned in a randomized manner. Animals were excluded from the study if weight loss exceeded 20% of the original body weight. No blinding was implemented in this study. Although experimenters were aware of group assignment during animal procedures and data analysis, all animals were handled identically, and all measurements and analyses were conducted with non-biased standardized, predefined protocols. These measures were incorporated to minimize procedural variability and reduce the risk of observer bias.

### Laser-induced choroidal neovascularization (CNV) model

12-week-old *C57BL/6 J* or 10-week-old *Adipoq:Cre* and *Adipoq:Prdm16* or recipient *Adipoq:Prdm16* male mice were anesthetized intraperitoneally with a 10% ketamine and 4% xylazine mix (10 μl/g of body weight), and pupils were dilated with Mydriacyl 1% (Alcon, #DIN 00001007). Using an argon laser, we ruptured their Bruch’s membrane to induce choroidal neovascularization (CNV). Each eye received 4 distinct laser burns with the following settings (400 mW, 50 μm, 0.05 s). Non-lasered, but handled animals served as controls. Fourteen days after laser, mice were sedated with isoflurane gas and euthanized by cervical dislocation before proceeding to adipose tissue collection for western blot and RNA extraction as well as histology assessment. Adipose depots were collected as follows: epididymal white adipose tissue (eWAT): bilateral intra-abdominal visceral depot attached to the epididymis; interscapular brown adipose tissue (iBAT), bilobed tissue between the scapulae; inguinal white adipose tissue (iWAT), bilateral superficial subcutaneous between the skin and muscle fascia just anterior to the lower segment of the hind limbs; retro orbital adipose tissue (rOAT): located around the optic nerve behind the posterior pole of the eyeball. 3 and 7 days after laser *Adipoq:Cre* and *Adipoq:Prdm16* male mice were sedated with isoflurane gas and euthanized by cervical dislocation before proceeding to the collection of eyes. RPE-choroid-sclera complexes were isolated to perform RNA extraction. Age-matched littermates were randomly distributed in the different experimental groups. All experiments were reproduced in independent cohorts at least three times.

### Plasma catecholamine measurement

Plasma adrenaline and noradrenaline concentrations were measured from 12-week-old lasered CNV and sham C57BL/6J mice at 3- and 7-days post CNV on a 1290 Infinity-6460 Triple Quad LC/MS instrument from Agilent. Quantification was achieved using calibrators from Ceriliant (C-109, D-081) and the Agilent MassHunter Quantitative analysis software.

### Tamoxifen treatment in vivo

Tamoxifen (Sigma, #T5648) was dissolved in corn oil (Sigma, #C8267) to a 25 mg/mL final concentration at 37 °C with shaking. To induce adipose-specific Cre-recombination, 8-week-old *Adipoq:Cre* and *Adipoq:Prdm16* mice were gavaged once daily with 5 mg of tamoxifen for 4 consecutive days.

### Choroidal neovascularization evaluation

14 days after laser choroidal neovascularization (CNV) was evaluated. Briefly, mice were sedated with isoflurane gas and intracardially perfused with 15 mg/mL of fluorescein isothiocyanate (FITC)-dextran (Sigma, #FD2000S) and euthanized. Eye globes were enucleated and fixed in PFA 4% at RT for 30 min. RPE-choroid-sclera complex was dissected and incubated in PBS + 0.1% Triton X-100 for 30 min at RT, followed by an overnight incubation with rhodamine-labeled Griffonia (bandeiraea) Simplicifolia Isolectin I (Vector Laboratories, #RL-1102) (diluted at 1:100). The next day, RPE-choroid-sclera complexes were washed with PBS 1× and tissues were mounted onto a glass slide. For each sample, Z-stacks were taken at 30X on Olympus FluoView FV1000 laser scanning confocal inverted microscope (Olympus Canada, Richmond Hill, ON). Analysis was performed on Z-stacks compressed on single images, the area of neovascularization (corresponding to FITC–dextran-positive signal) and the burn area (Isolectin-positive signal) were measured using ImageJ software (Version 1.0; U. S. National Institutes of Health, Bethesda, Maryland, USA).

### Western blot

eWAT, iBAT and iWAT protein extraction was performed as described before (Diaz Marin et al, 2019). For the RPE-choroid-sclera complexes, the eyes were enucleated, and RPE-choroid-sclera complexes were dissected and homogenized in RIPA 1X (Cell Signaling Technologies, #9806). Whole eWAT, iBAT, iWAT, and RPE-choroid-sclera extracts protein levels were quantified by BCA assay (Sigma #QPBCA). Samples were analyzed by standard SDS-PAGE and western blotting with antibodies in the Reagents and Tools Table. For all samples, 50 μg protein were analyzed for each condition.

### RT-qPCR

Adipose tissue was collected at 14 days after laser and kept at -80 °C until RNA extraction. 100 mg of eWAT and iWAT as well as one full-fat pad of iBAT were used for RNA extraction per sample. For rOAT RNA extraction, a pool of 10 fat pads was used per sample. 1 RPE-choroid-sclera complex was used for RNA extraction per sample. Total RNA was isolated using Trizol reagent (Life Technologies, #15596018) following the manufacturer's instructions. 1 µg of RNA was used for reverse transcription using 5× iScript RT Supermix (BIO-RAD, #1708841). Quantitative real-time PCR (qRT-PCR) was performed with iTaq Universal SYBR® Green Supermix (BIO-RAD, #1725124) following the manufacturer's instructions. Sequences of the primers used are listed in the Reagents and Tools Table. RT-qPCR results were normalized to the housekeeping genes *Tbp* mRNA levels when analyzing adipose tissue and the housekeeping gene *Actb* when analyzing RPE-choroid-sclera. The results are expressed in folds of change of mRNA expression, as the relative mRNA level of a specific gene expression using the formula ΔΔCt=2^-ΔCt^. Real-time PCR assays were run on a 7500 Real-Time PCR System (Applied Biosystems).

### Histology analysis

Collected adipose tissues were fixed in Formalin 10% O/N followed by standardized paraffin-embedding. Paraffin-embedded tissues were cut into 12-µm thick sections. Immunohistochemistry (IHC) was performed with Mouse and Rabbit specific HRP/DAB (ABC) Detection IHC kit (Abcam, #ab64264) according to manufacturer instructions. Samples were incubated overnight with anti-rabbit UCP1 (ThermoFisher Scientific, #PA1-24894, RRID: AB_2241459] primary antibody (diluted at 1:500) or anti-rabbit PRDM16 (Abcam, # ab106410, RRID: AB_10866455] primary antibody (diluted at 1:500) at 4 °C. Samples were mounted with Permount (Fisher Chemical, #UN1294). For each sample, representative DIC images were taken with a Zeiss Axio-Imager Z2 (Zeiss) with a coupled AxioCam ICc 1 (Zeiss). Zeiss Axio Vision software (Zeiss) was used for image processing and editing.

### Adipose tissue fluorescence-activated cell sorting (FACS)

eWAT, iBAT, and iWAT fat pads were freshly dissected, homogenized using scissors, and incubated in 10 mL of DMEM F12 supplemented with 1 mg/ml of Collagenase Type II (Sigma, # C6885) for a maximum of 45 min at 37 °C with intense shaking every 10 min. The digestion was stopped with 10 mM EDTA (MJS Biolynx, #UB15694S2) and samples were incubated for an extra 5 min at RT. Samples were filtered through a 100 µm strainer (Falcon, #352360) and centrifuged at 1000 × *g* for 10 min at 4 °C to separate the mature adipocytes and the stromal vascular fraction (SVF). Mature adipocytes and supernatant were discarded, and red blood cells were removed by resuspending the SVF-containing pellet with RBC lysis buffer (eBioscience, #00-4333-57) at RT for 5 min. RBC lysis buffer was neutralized with PBS 1× and samples were centrifuged at 500 × *g* for 10 min at 4 °C. After centrifugation, SVF cells were counted, resuspended in PBS + 3% FBS, and filtered through a 100-µm strainer. Viability of the cells was checked by Zombie Aqua (Biolegend, #423101) staining for 15 min at room temperature. Samples were incubated with LEAF purified anti-mouse CD16/32 (Biolegend, #101330) for 10 min at 4 °C to block FC receptors. Cells were then incubated for 25 min at 4 °C with the following antibodies: BV711 anti-mouse/human CD11b (BioLegend, #101242), PE anti-mouse F4/80 (BioLegend, #123110), APC anti-mouse CD64 (BioLegend, #139305), FITC anti-mouse CD38 (BioLegend, #102705), APC/Cy7 anti-mouse Ly-6G (BioLegend, #127624), and BV785 anti-mouse CD11c (BioLegend, #117335). Data was acquired using a Fortessa X-20 (BD Biosciences) with DB FACSDiva^TM^ Software (BD Biosciences) and analyzed using FlowJo^TM^ v10.8 Software (BD Life Sciences).

### β3-adrenergic activation

Mice were injected intraperitoneally once daily for 5 consecutive days with CL316,243 (Sigma, #C5976) at 1 mg/kg of weight.

### Adipose tissue transplantation (ATT) model

8-week-old recipient *Adipoq:Prdm16* mice were gavaged once daily with 5 mg of tamoxifen for 4 consecutive days and randomly assigned to the different groups independently of the origin of the donor iWAT. Donor *Adipoq:Prdm16* mice were injected intraperitoneally once daily for 5 consecutive days with CL316,243 (Sigma, #C5976) at 1 mg/kg of weight. Following β3-adrenergic activation, donor *Adipoq:Prdm16* mice were anesthetized, sacrificed, and their iWAT fat pads were collected and washed in sterile PBS 1X. At the same time, 10-week-old recipient *Adipoq:Prdm16* mice were anesthetized with isoflurane, the implant area was shaved and cleaned with 70% EtOH, and subjected to 2–3 dorsal incisions of 1 mm to allow subcutaneous engraftment of 200 mg of donor iWAT (stimulated with CL316,243). Dorsal incisions were closed using 7.5 × 1.75 mm suture clips (Fine Science Tools, #12040-01) disinfected in 70% EtOH. Mice were closely monitored for three weeks before performing laser-induced choroidal neovascularization. 14 days after laser, CNV was evaluated, and eWAT, iBAT, and iWAT fat pads were collected, and successful engraftment of the transplanted tissues was verified. We prespecified that if animals became infected or transplanted tissues became necrotic, the animals would be excluded from the study. However, we did not observe any adverse events.

### Isolation of iWAT primary inguinal preadipocytes

iWAT fat pads from male *Adipoq:Prdm16*^*+/+*^ mice were freshly dissected, homogenized using scissors, and incubated in 10 mL of DMEM F12 (Corning, #10-090-CV) supplemented with 1 mg/ml of Collagenase Type II (Sigma, # C6885) for a maximum of 45 min at 37 °C with intense shaking every 10 min. The digestion was stopped with EDTA (MJS Biolynx, #UB15694S2) 10 mM and samples were incubated 5 extra minutes at RT. Samples were filtered through a 70 µm strainer (Falcon, #352350) and centrifuged at 1000 × g for 10 min at 4 °C to separate the mature adipocytes and the stromal vascular fraction (SVF). Mature adipocytes and supernatant were discarded, and red blood cells were removed by resuspending the SVF-containing pellet with RBC lysis buffer (eBioscience, #00-4333-57) at RT for 5 min. RBC lysis buffer was neutralized with PBS 1×, and samples were centrifuged at 500 × *g* for 10 min at 4 °C. After centrifugation, SVF cells were counted, resuspended in DMEM F12 + 10% FBS + 1%P/S, and filtered through a 70-µm strainer. SVF cells were seeded in a 100 mm dish (Falcon, #353003) at a ratio of 6 fat pads/100 mm dish and left in an incubator at 37 °C with 5% CO_2_ for 1 h. Afterward, the medium was removed, cells were washed with DMEM F12 basal three times to remove floating fat. DMEM F12 + 10% FBS + 1%P/S was added to the cells, and medium was changed every 48 h. When cells reached 80% confluency, cells were split into an experimental format in 6-well plates at 50,000–80,000 cells per well.

### Differentiation of primary inguinal preadipocytes

When cells reached 80% confluence, cells were induced to undergo differentiation into adipocytes (day 0 of differentiation) with Dulbecco’s modified Eagle’s medium (DMEM, Corning, #10-017-CV) supplemented with 10% Fetal bovine serum (FBS, Multicell, #090450)) and 1% Penicillin/Streptomycin (P/S, Corning, #30-00-CI); with the classic adipogenic cocktail composed of 0.5 mM 3-isobutyl-1-methylxanthine (IBMX, Sigma, #I5879), 1 µM dexamethasone (Sigma, #D2915) and 170 nM insulin (Sigma, #I9278). After 48 h (day 2 of differentiation), media was changed for maintenance medium (DMEM + 10%FBS + 170 nM insulin+1%P/S). Medium was changed every 2 days until day 8 of differentiation.

### Tamoxifen treatment in vitro

The knockout of *Prdm16* was induced in primary inguinal preadipocytes isolated from *Adipoq:Prdm16* mice with 5 µM 4-hydroxy-tamoxifen (Sigma, #H7904) dissolved in 100% ETOH for 3 consecutive days during the growth phase (before cell differentiation).

### Choroid explant assay

Eyes from C57BL/6J mice were enucleated, and choroids dissected. Segmented choroids were plated into 24-well culture plates and covered with growth factor-reduced Matrigel (Corning, #356230) and cultured in EBM-2 media (Lonza, #CC-3156) supplemented with EGM-2 SingleQuots (Lonza, #CC-4176) for 48 h at 37 °C with 5% CO2. 48 h after, EGM-2 was replaced by serum-free conditioned media from *Adipoq:Prdm16* primary inguinal adipocytes treated with EtOH or 4-hydroxy-tamoxifen. For denaturation experiments media was boiled at 100 °C for 5 min. Phase contrast photos of individual explants were taken daily using a ZEISS Axio Oberver.Z1 microscope (Zeiss). The areas of sprouting were quantified with ImageJ software (Version 1.0).

### On‑bead digestion using protein aggregation capture (PAC)

Samples were denatured in 2 M urea and reduced with 9 mM DTT in 50 mM ammonium bicarbonate at 37 °C for 30 min (700 RPM). After cooling, samples were alkylated with 17 mM IAA for 30 min in the dark. Residual nucleic acids were removed by treatment with 26 U benzonase and 2 mM MgCl₂ at 37 °C for 60 min. Protein Aggregation Capture was performed using 45 µL Hydroxyl PAC beads (ReSyn Biosciences) and 50% acetonitrile. Samples were incubated 25 min at 1000 RPM, placed on a magnetic rack, and the beads were washed 3× with 70% ethanol. Beads were resuspended in 0.03 µg/µL Lys‑C/Trypsin (Promega) in 50 mM ammonium bicarbonate, briefly sonicated, and digested overnight at 37 °C. Digests were collected, beads rinsed with 35 µL ammonium bicarbonate, and pooled. Samples were acidified to 0.5% formic acid, dried in a SpeedVac, and stored at −20 °C.

### LC‑MS/MS analysis

Peptides were reconstituted in 2% ACN/1% FA and loaded onto a 75 µm × 170 mm C18 column (Dr. Maisch) on a Vanquish Neo LC system (Thermo Scientific). Separation was performed at 500 nL/min using a linear gradient of 3–36% buffer B (108 min), then 36–55% (15 min). The LC was coupled to an Orbitrap Fusion with a Nanospray Flex source (1.3–1.7 kV, 50 V S‑lens, 250 °C). Full MS spectra were acquired in the Orbitrap (Thermo Scientific, *m/z* 360–1500, 120,000 resolution, AGC 8 × 10⁵). The top 25 precursors were fragmented by HCD (NCE 29) and analyzed in the ion trap (AGC 1.4 × 10⁴), with 4 s dynamic exclusion.

### Protein identification

Peak lists were generated using Proteome Discoverer 2.4. Searches were performed with Mascot 2.6 (Matrix Science) against the Mus musculus UniProt proteome (version 2025‑04‑14), using 10 ppm precursor and 0.02 Da fragment tolerances. Trypsin, 1 missed cleavage, carbamidomethyl‑Cys (fixed), and oxidized Met (variable) were specified. Data validation was performed in Scaffold 4.8.

### Reactome analysis

LC-MS/MS was analyzed in DDA mode, and protein expression was determined using the top 3 algorithm. Reactome proteome analysis was performed with the Correlation Adjusted MEan RAnk gene set test (Camera) (Griss et al, 2020). Linear models (limma) statistics were represented in table format, with Log2 fold change, average expression, t-statistic, *P* value, adjusted *P* value (*P* value corrected for multiple testing using the Benjamini-Hochberg method), and the B-statistic of the log-odds of differential expression.

### Statistical analyses

All results are presented as mean ± SEM unless indicated otherwise. Analyses and statistical significance were analyzed using GraphPad Prism 9.0 (GraphPad Software, San Diego, CA; www.graphpad.com) by one or two-way ANOVA, when comparing multiple groups, and two-tailed unpaired Student’s *t* test, when comparing only two groups. Statistical significance was considered when *P* < 0.05, and it is indicated as: *P < 0.05, **P < 0.01, and ***P < 0.001, the exact *P* values are indicated. Biological experiment numbers are listed in figure legends. All experiments were repeated at least three times, and N is indicated in the figure or in the figure legends.

## Supplementary information

Peer Review File

Source data Fig. 1

Source data Fig. 2

Source data Fig. 3

Source data Fig. 4

Figure EV1 Source Data

Figure EV4 Source Data

Figure EV5 Source Data

Figure EV6 Source Data

Expanded View Figures

## Data Availability

Data has been deposited to the following repository: https://www.ebi.ac.uk/biostudies/bioimages/studies/S-BIAD2218?key=abeb6dc2-5d3a-4ba3-a446-cf0bed9f35e4. Proteomics data has been deposited in the MassIVE database: ftp://massive-ftp.ucsd.edu/v12/MSV000101480/. The source data of this paper are collected in the following database record: biostudies:S-SCDT-10_1038-S44321-026-00441-5.
