# Supplementary figures and images for: Adipocytes influence choroidal neovascularization via PRDM16
Source: EMBO Mol Med. 2026 May 19;18(6):2379–403. doi: 10.1038/s44321-026-00441-5 (PMC13269495; doi:10.1038/s44321-026-00441-5)

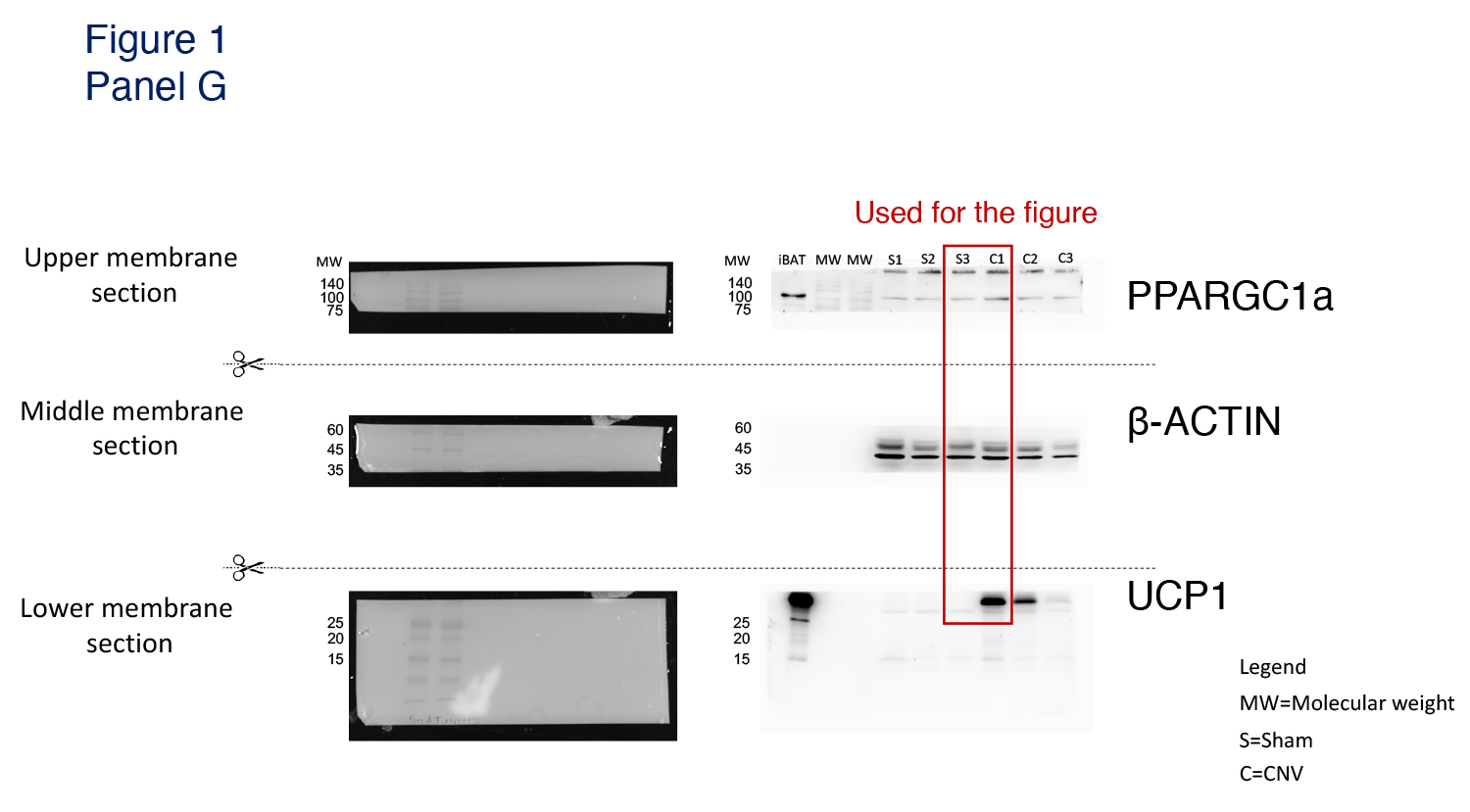

Supplement: Supplementary file 2 — Source data Fig. 1 [file 44321_2026_441_MOESM2_ESM.zip › Figure 1/Fig1G.tif]

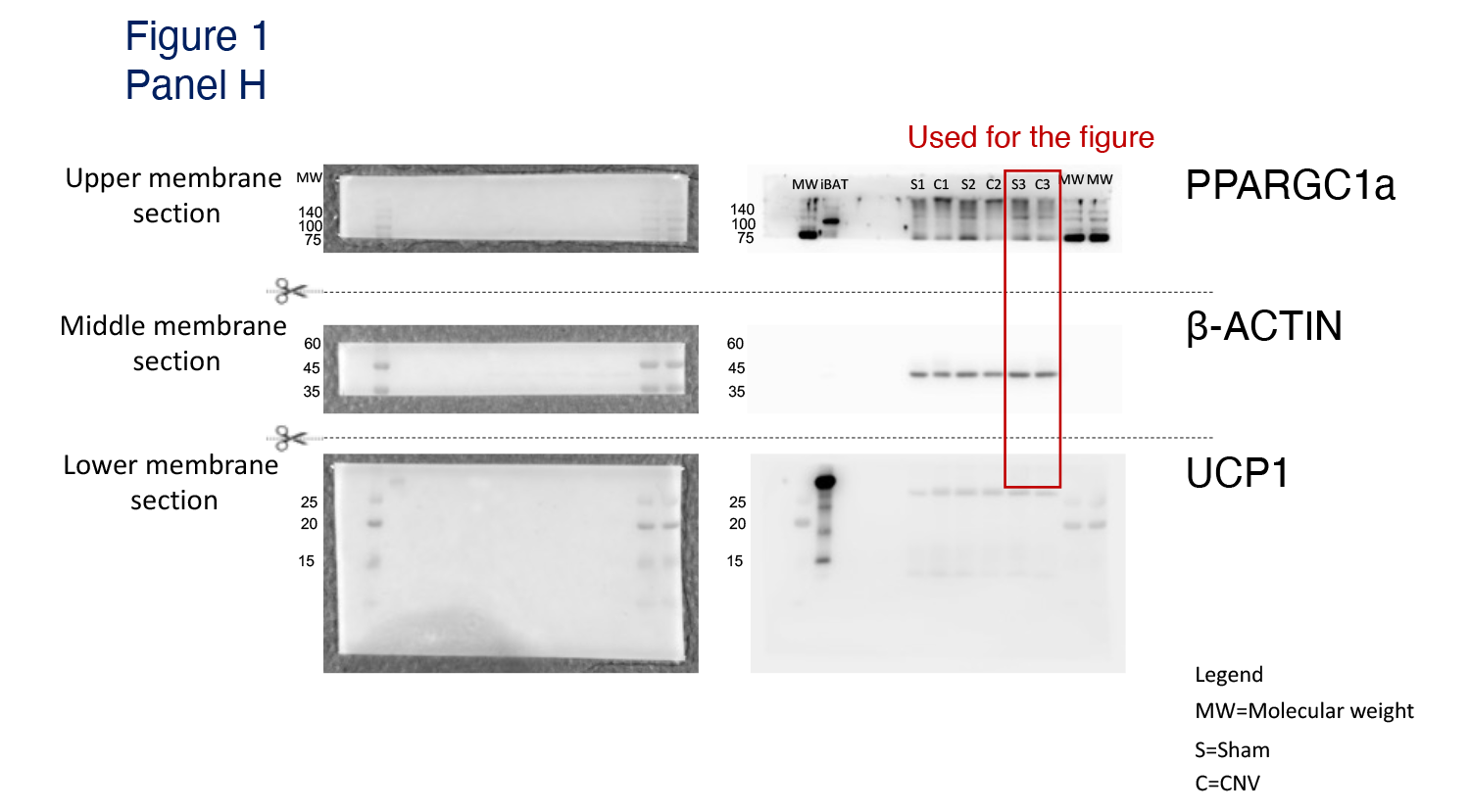

Supplement: Supplementary file 2 — Source data Fig. 1 [file 44321_2026_441_MOESM2_ESM.zip › Figure 1/Fig1H.tif]

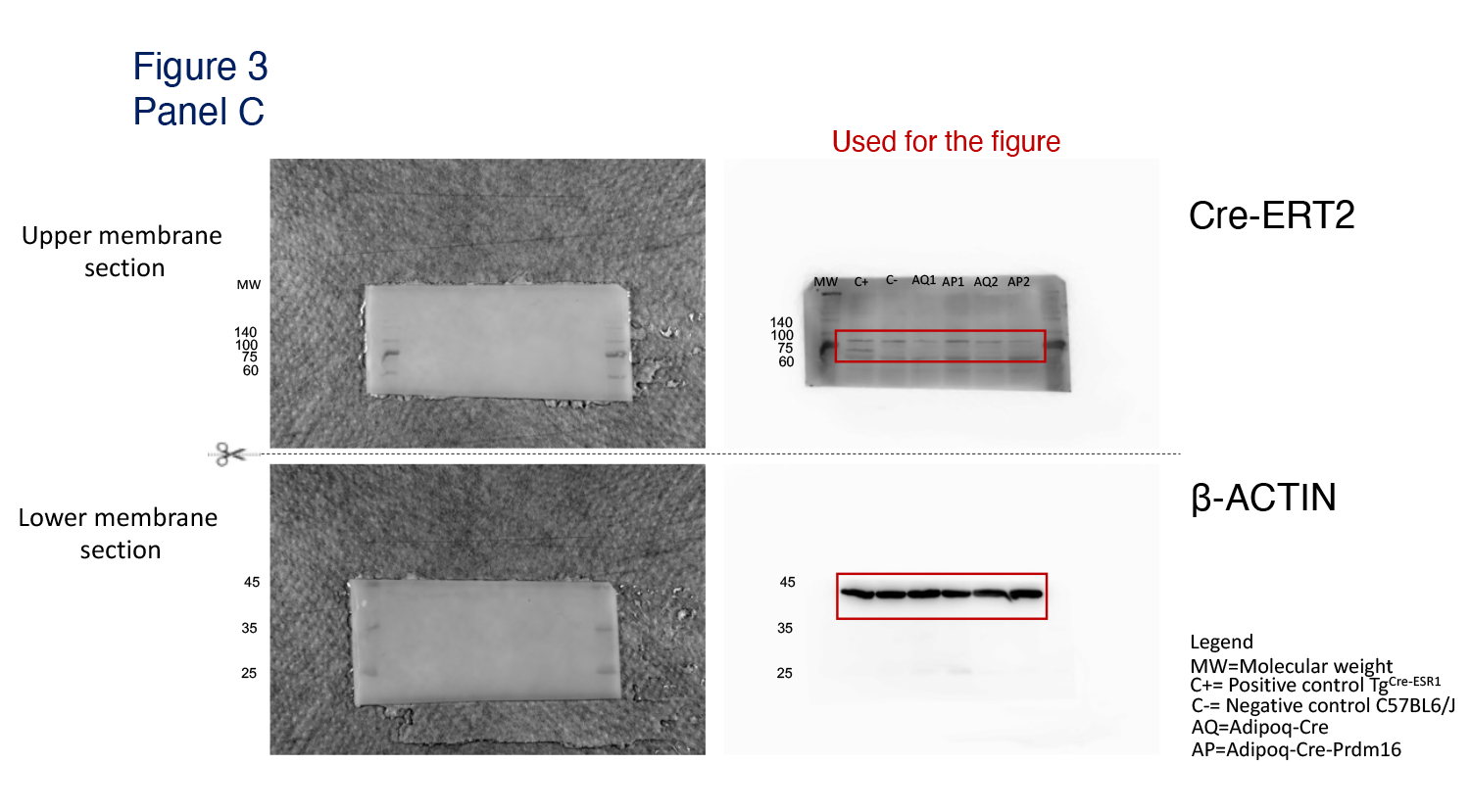

Supplement: Supplementary file 4 — Source data Fig. 3 [file 44321_2026_441_MOESM4_ESM.zip › Figure 3/Fig3C continued.tif]

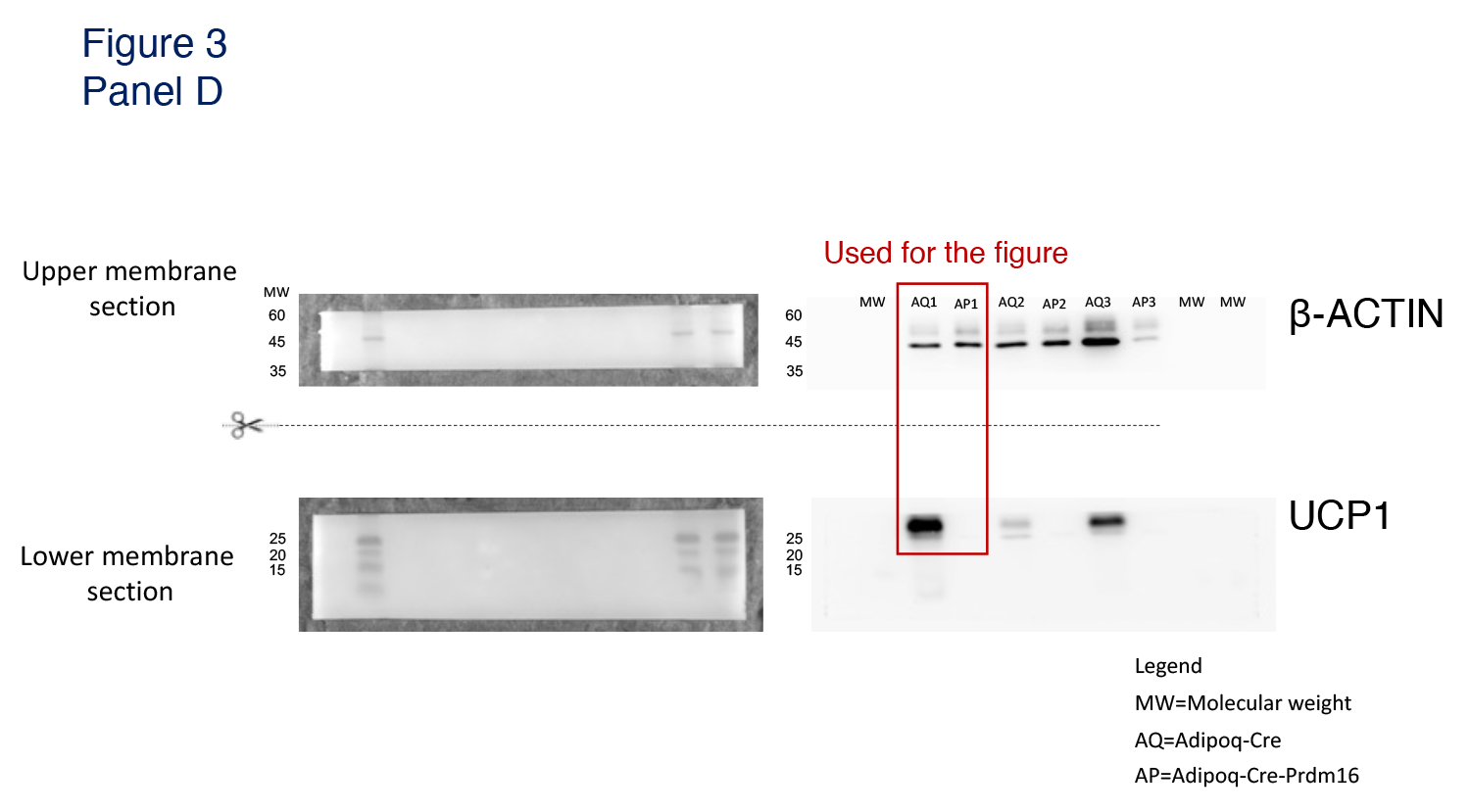

Supplement: Supplementary file 4 — Source data Fig. 3 [file 44321_2026_441_MOESM4_ESM.zip › Figure 3/Fig3D.tif]

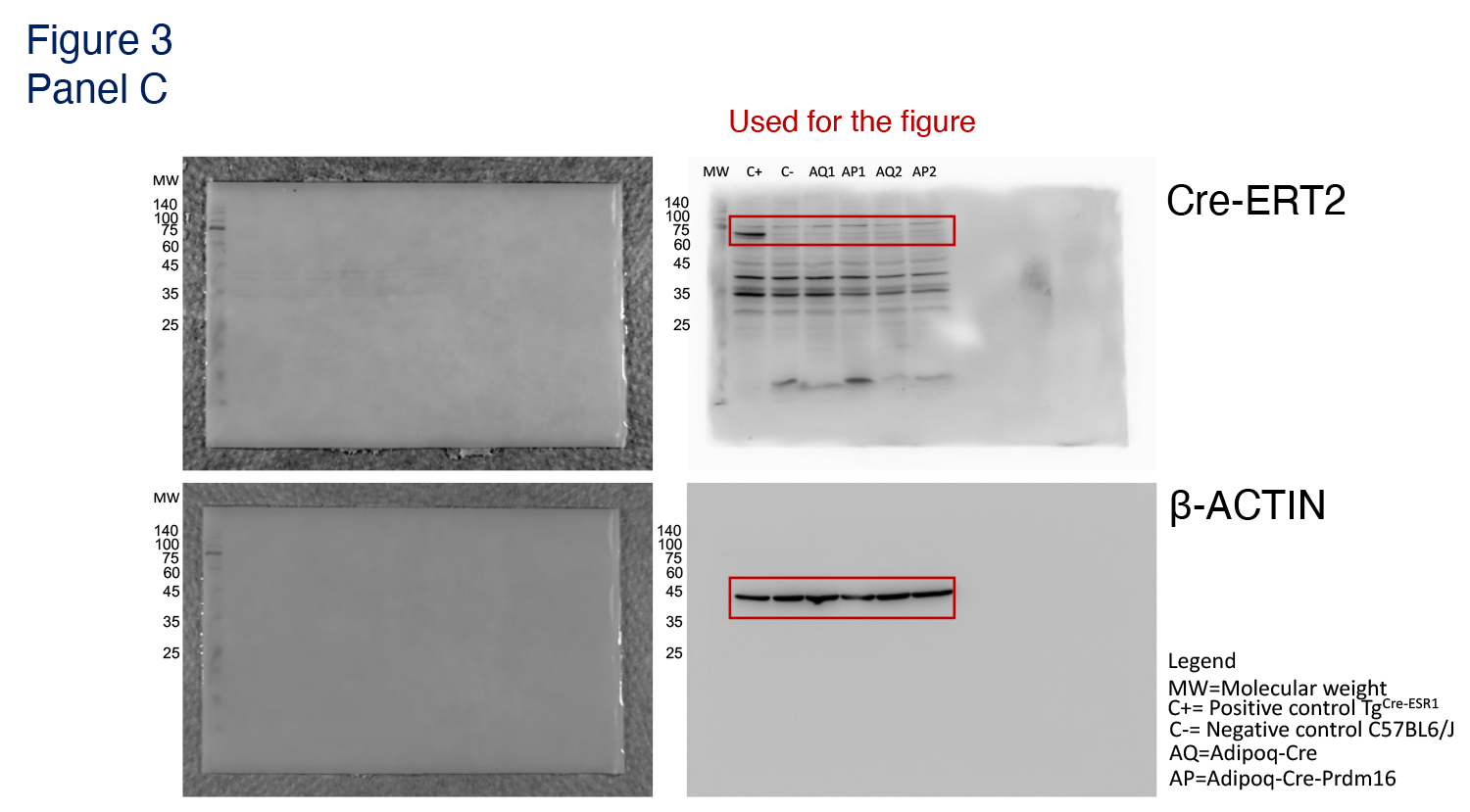

Supplement: Supplementary file 4 — Source data Fig. 3 [file 44321_2026_441_MOESM4_ESM.zip › Figure 3/Fig3C.tif]

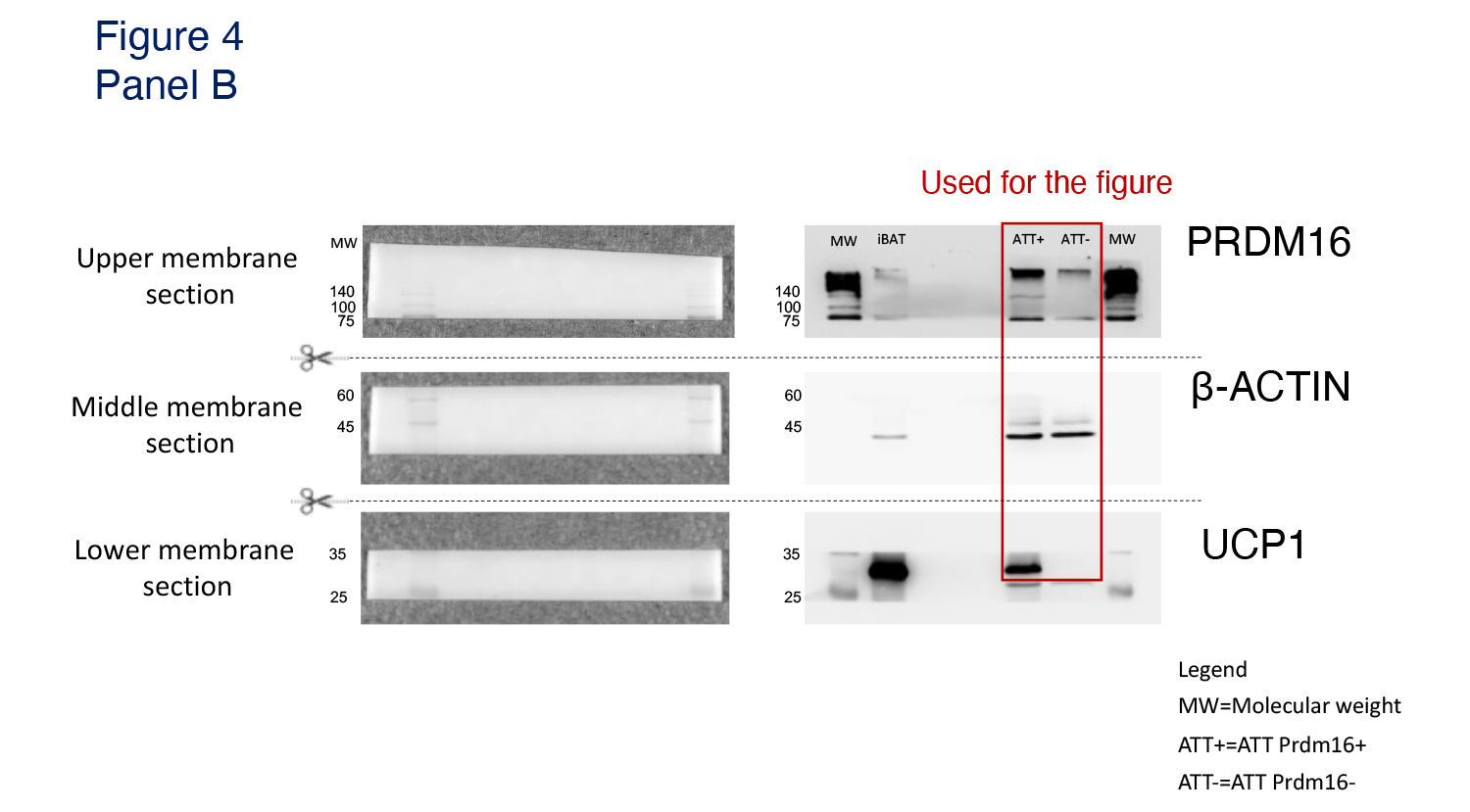

Supplement: Supplementary file 5 — Source data Fig. 4 [file 44321_2026_441_MOESM5_ESM.zip › Figure 4/Fig4B.tif]

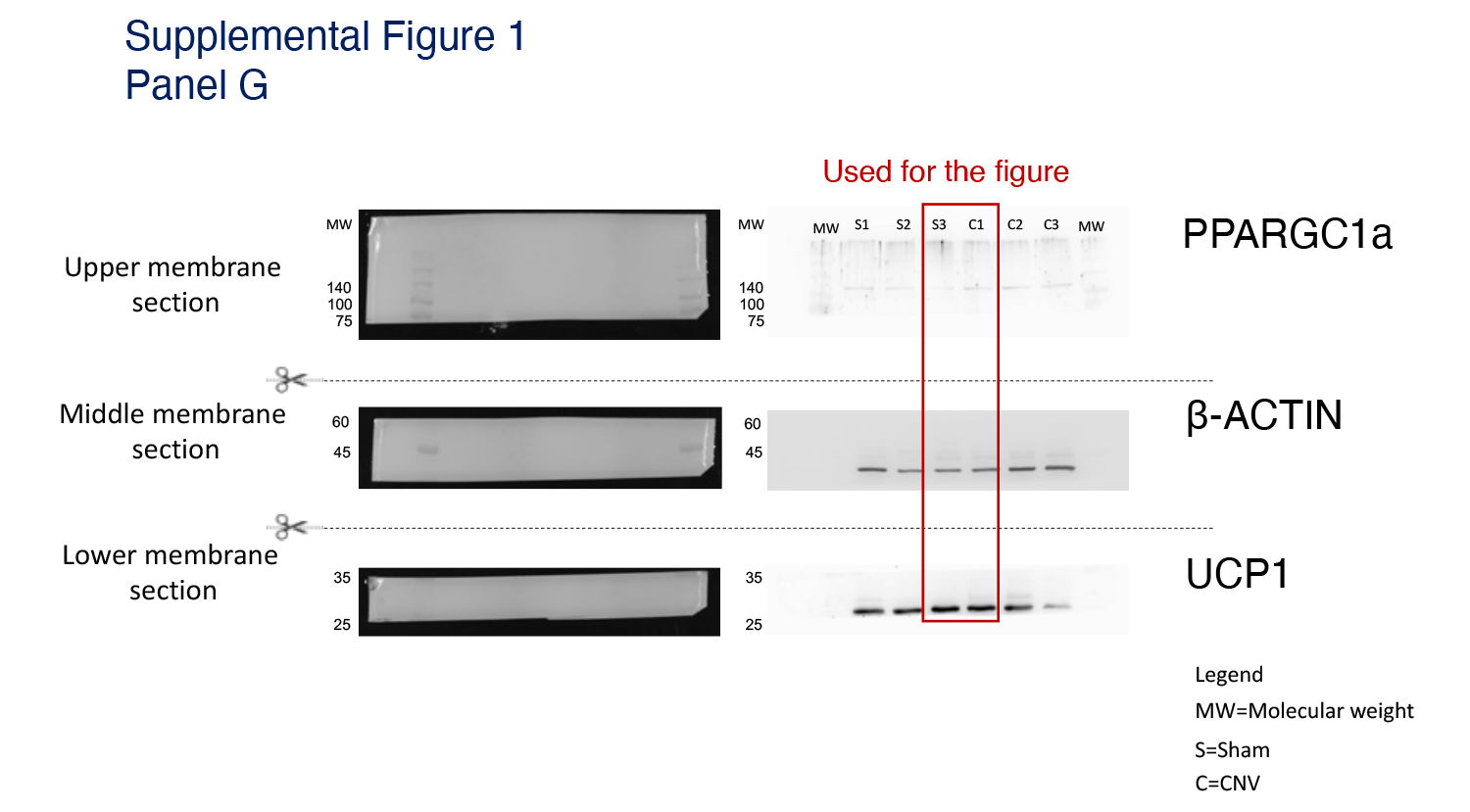

Supplement: Supplementary file 6 — Figure EV1 Source Data [file 44321_2026_441_MOESM6_ESM.zip › Figure EV1/FigEV1G.tif]

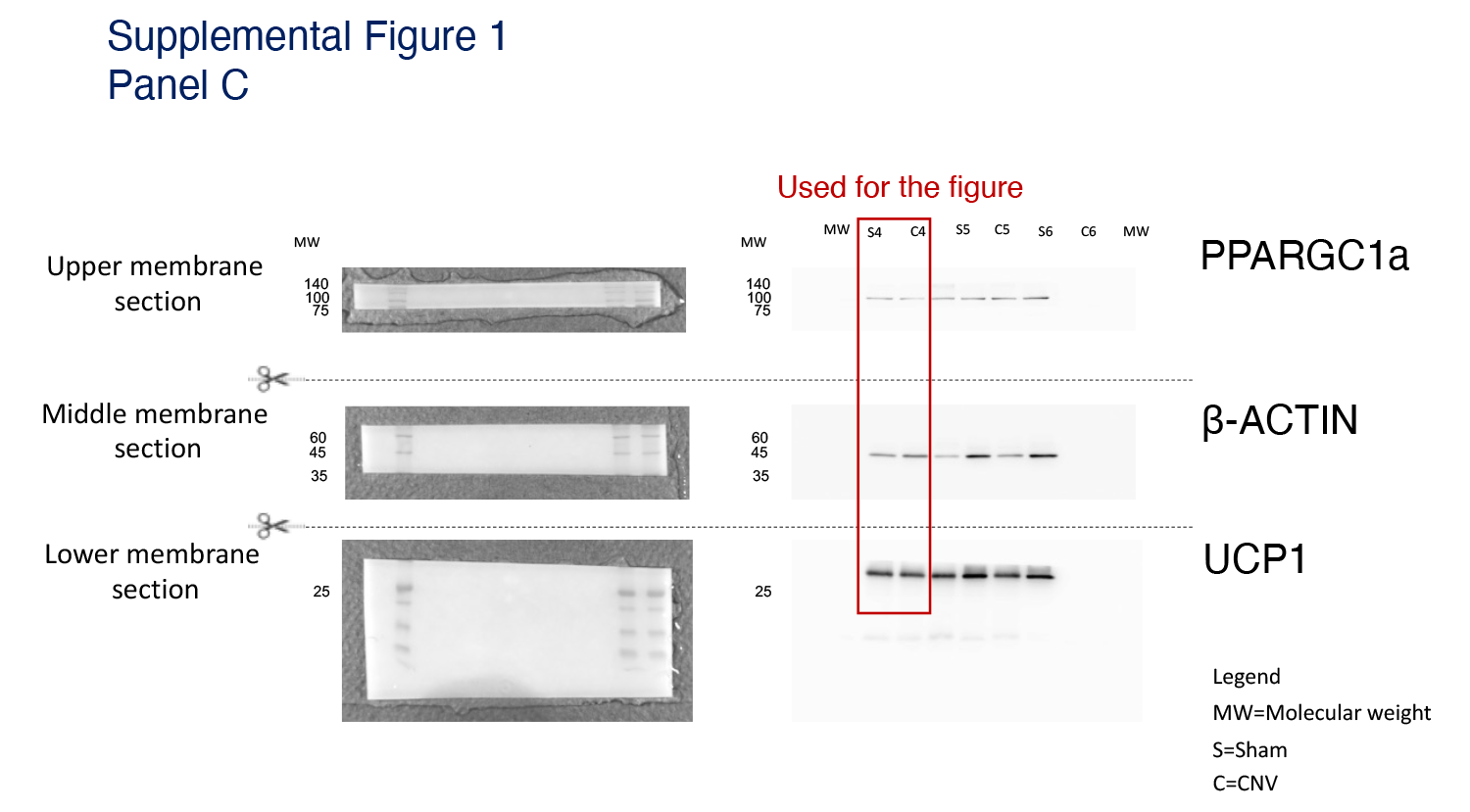

Supplement: Supplementary file 6 — Figure EV1 Source Data [file 44321_2026_441_MOESM6_ESM.zip › Figure EV1/FigEV1C.tif]

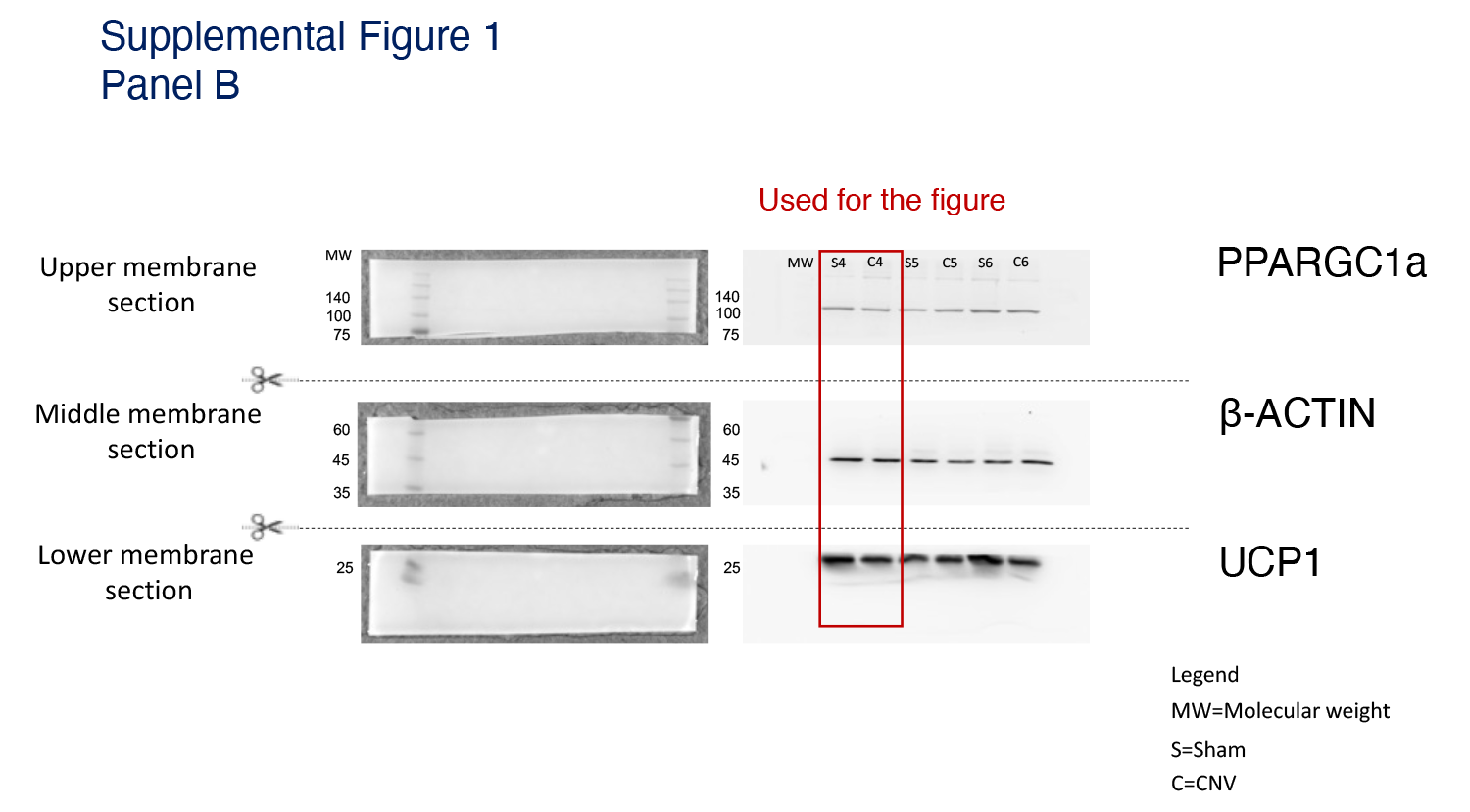

Supplement: Supplementary file 6 — Figure EV1 Source Data [file 44321_2026_441_MOESM6_ESM.zip › Figure EV1/FigEV1B.tif]

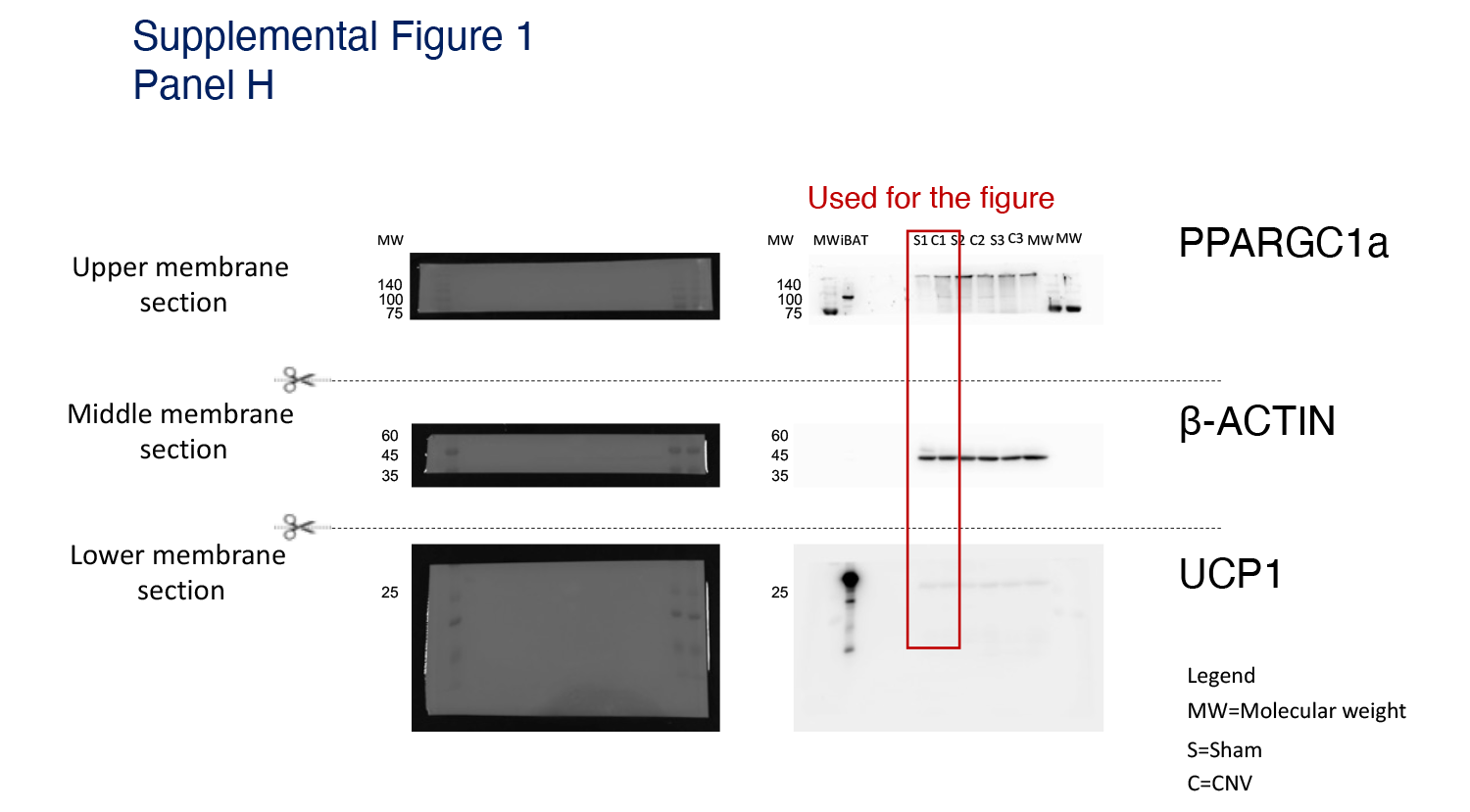

Supplement: Supplementary file 6 — Figure EV1 Source Data [file 44321_2026_441_MOESM6_ESM.zip › Figure EV1/FigEV1H.tif]

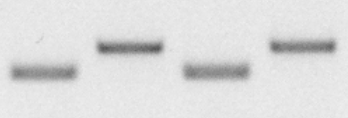

Supplement: Supplementary file 8 — Figure EV5 Source Data [file 44321_2026_441_MOESM8_ESM.zip › Figure EV5/FigEV5B/2020-07-20 Genotyping prdm16_crop for figure.tif]

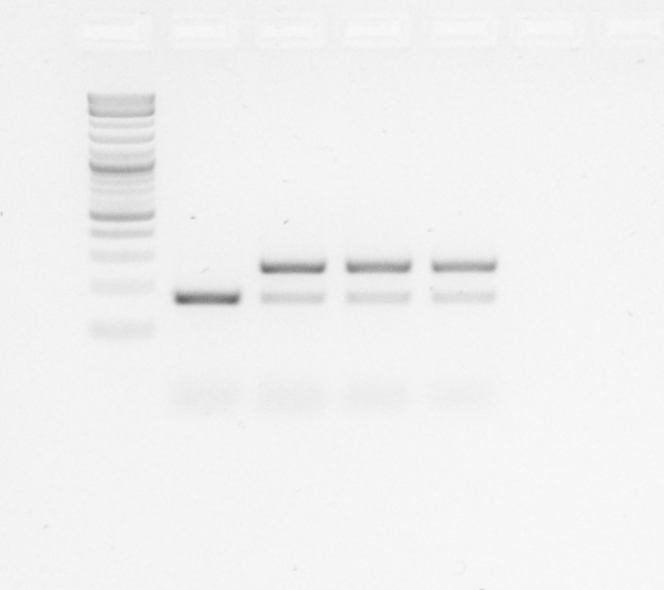

Supplement: Supplementary file 8 — Figure EV5 Source Data [file 44321_2026_441_MOESM8_ESM.zip › Figure EV5/FigEV5B/2020-07-20 Genotyping Adipoq_full gel.tif]

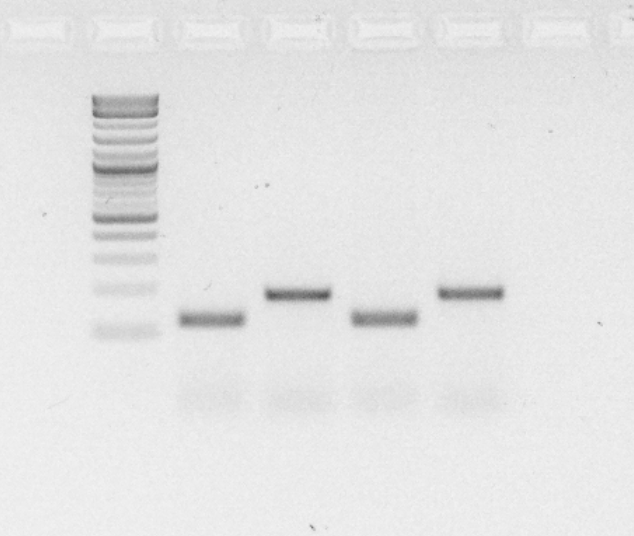

Supplement: Supplementary file 8 — Figure EV5 Source Data [file 44321_2026_441_MOESM8_ESM.zip › Figure EV5/FigEV5B/2020-07-20 Genotyping prdm16_full geltif]

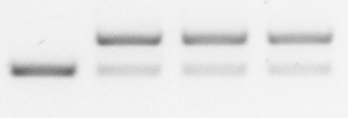

Supplement: Supplementary file 8 — Figure EV5 Source Data [file 44321_2026_441_MOESM8_ESM.zip › Figure EV5/FigEV5B/2020-07-20 Genotyping Adipoq_crop for figure.tif]
