# Supplementary material for: Adipocytes influence choroidal neovascularization via PRDM16
Source: EMBO Mol Med. 2026 May 19;18(6):2379–403. doi: 10.1038/s44321-026-00441-5 (PMC13269495; doi:10.1038/s44321-026-00441-5)
Supplement: Supplementary file 7 — Figure EV4 Source Data [file 44321_2026_441_MOESM7_ESM.zip › Figure EV4/FigEV4D-F/001.pdf]

CIUSSS de l'Est-de-l'Ile-de-Montréal
Hôpital Maisonneuve-Rosemont
5415 boul. de l'Assomption, Montréal (Québec), H1T 2M4
Biochimie, Diagnostic moléculaire, Hématologie, Microbiologie, Thérapie cellulaire

FINAL
#Accès: 80122223

Projet de IRE1a dans l'obésité
Chambre:
LABO, LABORATOIRE
#Permis:

RAMQ :
Nom : **5951**
Prénom : **RECHERCHE**
Nais : 1900/01/01 Sexe: N
Adresse:

Dossier: **T000653688**

Tél: ( ) - Fax: ( ) - Tél :

BIO-ENDOCRINOLOGIE

ANALYSE(S)	RESULTAT(S)	ALARME	VALEURS REF	UNITES	SIGN.
**spécimen prélevé 18/06/14 10:03 reçu 18/12/12 10:08**					
**CATÉCHOLAMINES PLASMATIQUES**					
Tension artérielle	?				V/AUT
Adrénaline	334		couché: <450<br>debout: <600	pmol/L	MIRVA
Noradrénaline	1409		couché: <2400<br>debout: <3600	pmol/L	MIRVA
Dopamine	<61		< 300	pmol/L	MIRVA

Une augmentation inférieure à 2 fois la limite supérieure des valeurs de référence peut être causée par des processus physiologiques, la prise de médicaments ou un mauvais prélèvement.

(*) _ V/AUT

Analyse(s) développée(s) et validée(s) par le département de biochimie de l'HMR (LC-MS/MS). Les résultats ne doivent pas être utilisés comme les seuls outils pour le diagnostic ou le suivi des traitements.

ROBRO

Validé par: ROBITAILLE, ROBERT

Légende: AN=Anormal H=Haut B=Bas C=Critique

RAPPORT INSTANTANÉ Imprimé le: 2019/03/11 11:39

Biochimistes cliniques: K.Benkirane, V.De Guire, M.-E.Gingras, A.Lagana-Teyssier, M.Provençal, R.Robitaille
Hématologues: Drs I.Ahmad, N.Bambace, J.Beaudet, D.Bélanger, R.Bélanger, J.Bergeron, L.Bernard, L.Busque, S.Cohen, J.S.Delisle, I.Fleury, J.Hébert, J.Kassis, T.Kiss, S.Lachance, R.LeBlanc, C.Letendre, F.Letendre, L.Mollica, J.Noujaim, C.Perreault, D-C.Roy, J-L Dionne, J.Roy, G.Sauvageau, J.St-Louis
Microbiologistes: Drs C.Béliveau, A.Couture-Cossette, S.Dufresne, Y.Émond, A.-C.Labbé, C.Lavallée, X.Marchand-Sénécal, L.Poirier
Document confidentiel. Si vous avez reçu cette copie par erreur, SVP nous aviser.

CIUSSS de l'Est-de-l'Ile-de-Montréal
Hôpital Maisonneuve-Rosemont
5415 boul. de l'Assomption, Montréal (Québec), H1T 2M4
Biochimie, Diagnostic moléculaire, Hématologie, Microbiologie, Thérapie cellulaire

FINAL
#Accès: 80122200

Projet de IRE1a dans l'obésité	RAMQ :	Dossier: T000653684
Chambre:	Nom : 5942	
LABO, LABORATOIRE	Prénom : RECHERCHE	
#Permis:	Nais : 1900/01/01	Sexe: N
	Adresse:	
Tél: ( ) - Fax: ( ) -	Tél :	

BIO-ENDOCRINOLOGIE

ANALYSE(S)	RESULTAT(S)	ALARME	VALEURS REF	UNITES	SIGN.
**spécimen prélevé 18/06/14 10:00 reçu 18/12/12 10:08**					
**CATÉCHOLAMINES PLASMATIQUES**					
Tension artérielle	?				V/AUT
Adrénaline	394		couché: <450<br>debout: <600	pmol/L	MIRVA
Noradrénaline	6480		couché: <2400<br>debout: <3600	pmol/L	MIRVA
Dopamine	453		< 300	pmol/L	MIRVA

Une augmentation inférieure à 2 fois la limite supérieure des valeurs de référence peut être causée par des processus physiologiques, la prise de médicaments ou un mauvais prélèvement.

(*) _ V/AUT

Analyse(s) développée(s) et validée(s) par le département de biochimie de l'HMR (LC-MS/MS). Les résultats ne doivent pas être utilisés comme les seuls outils pour le diagnostic ou le suivi des traitements.

ROBRO

Validé par: ROBITAILLE, ROBERT

Légende: AN=Anormal H=Haut B=Bas C=Critique

RAPPORT INSTANTANÉ Imprimé le: 2019/03/11 11:35

Biochimistes cliniques: K.Benkirane, V.De Guire, M.-E.Gingras, A.Lagana-Teyssier, M.Provençal, R.Robitaille
Hématologues: Drs I.Ahmad, N.Bambace, J.Beaudet, D.Bélanger, R.Bélanger, J.Bergeron, L.Bernard, L.Busque, S.Cohen, J.S.Delisle, I.Fleury, J.Hébert, J.Kassis, T.Kiss, S.Lachance, R.LeBlanc, C.Letendre, F.Letendre, L.Mollica, J.Noujaim, C.Perreault, D-C.Roy, J-L Dionne, J.Roy, G.Sauvageau, J.St-Louis
Microbiologistes: Drs C.Béliveau, A.Couture-Cossette, S.Dufresne, Y.Émond, A.-C.Labbé, C.Lavallée, X.Marchand-Sénécal, L.Poirier
Document confidentiel. Si vous avez reçu cette copie par erreur, SVP nous aviser.

CIUSSS de l'Est-de-l'Ile-de-Montréal
Hôpital Maisonneuve-Rosemont
5415 boul. de l'Assomption, Montréal (Québec), H1T 2M4
Biochimie, Diagnostic moléculaire, Hématologie, Microbiologie, Thérapie cellulaire

FINAL
#Accès: 80122247

Projet de IRE1a dans l'obésité	RAMQ :	Dossier: **T000653691**
Chambre:	Nom : **5952**	
LABO, LABORATOIRE	Prénom : **RECHERCHE**	
#Permis:	Nais : 1900/01/01	Sexe: N
	Adresse:	
Tél:( ) - Fax: ( ) -	Tél :	

B I O - E N D O C R I N O L O G I E

ANALYSE(S)	RESULTAT(S)	ALARME	VALEURS REF	UNITES	SIGN.
**spécimen prélevé 18/06/14 10:05 reçu 18/12/12 10:08**					
**CATÉCHOLAMINES PLASMATIQUES**					
Tension artérielle	?				V/AUT
Adrénaline	2707		couché: <450<br>debout: <600	pmol/L	MIRVA
Noradrénaline	12264		couché: <2400<br>debout: <3600	pmol/L	MIRVA
Dopamine	:		< 300	pmol/L	MIRVA

Une augmentation inférieure à 2 fois la limite supérieure des valeurs de référence peut être causée par des processus physiologiques, la prise de médicaments ou un mauvais prélèvement.
Une dilution du spécimen a été nécessaire pour effectuer notre technique puisque la quantité de spécimen était insuffisante. Le résultat de cette dilution n'est pas concluant. Analyse impossible à réaliser. (Dopamine)

(*) V/AUT

Analyse(s) développée(s) et validée(s) par le département de biochimie de l'HMR (LC-MS/MS). Les résultats ne doivent pas être utilisés comme les seuls outils pour le diagnostic ou le suivi des traitements.

ROBRO

Validé par: ROBITAILLE, ROBERT

Légende: AN=Anormal H=Haut B=Bas C=Critique

RAPPORT INSTANTANÉ   Imprimé le: 2019/03/13 09:46

Biochimistes cliniques: K.Benkirane, V.De Guire, M.-E.Gingras, A.Lagana-Teyssier, M.Provençal, R.Robitaille
Hématologues: Drs I.Ahmad, N.Bambace, J.Beaudet, D.Bélanger, R.Bélanger, J.Bergeron, L.Bernard, L.Busque, S.Cohen, J.S.Delisle, I.Fleury, J.Hébert, J.Kassis, T.Kiss, S.Lachance, R.LeBlanc, C.Letendre, F.Letendre, L.Mollica, J.Noujaim, C.Perreault, D-C.Roy, J-L Dionne, J.Roy, G.Sauvageau, J.St-Louis
Microbiologistes: Drs C.Béliveau, A.Couture-Cossette, S.Dufresne, Y.Émond, A.-C.Labbé, C.Lavallée, X.Marchand-Sénécal, L.Poirier
Document confidentiel. Si vous avez reçu cette copie par erreur, SVP nous aviser.

CIUSSS de l'Est-de-l'Ile-de-Montréal
Hôpital Maisonneuve-Rosemont
5415 boul. de l'Assomption, Montréal (Québec), H1T 2M4
Biochimie, Diagnostic moléculaire, Hématologie, Microbiologie, Thérapie cellulaire

FINAL
#Accès: 80122204

Projet de IRE1a dans l'obésité	RAMQ :		Dossier: **T000653686**
Chambre:	Nom : **5943**		
LABO, LABORATOIRE	Prénom : **RECHERCHE**		
#Permis:	Nais : 1900/01/01	Sexe: N	
	Adresse:		
Tél:( ) - Fax: ( ) -	Tél :		

B I O - E N D O C R I N O L O G I E

ANALYSE(S)	RESULTAT(S)	ALARME	VALEURS REF	UNITES	SIGN.
	**spécimen prélevé 18/06/14 10:01 reçu 18/12/12 10:08**				
**CATÉCHOLAMINES PLASMATIQUES**					
Tension artérielle	?				V/AUT
Adrénaline	85		couché: <450<br>debout: <600	pmol/L	MIRVA
Noradrénaline	2154		couché: <2400<br>debout: <3600	pmol/L	MIRVA
Dopamine	104		< 300	pmol/L	MIRVA

Une augmentation inférieure à 2 fois la limite supérieure des valeurs de référence peut être causée par des processus physiologiques, la prise de médicaments ou un mauvais prélèvement.

(*) _ V/AUT

Analyse(s) développée(s) et validée(s) par le département de biochimie de l'HMR (LC-MS/MS). Les résultats ne doivent pas être utilisés comme les seuls outils pour le diagnostic ou le suivi des traitements.

ROBRO

Validé par: ROBITAILLE, ROBERT

Légende: AN=Anormal H=Haut B=Bas C=Critique

RAPPORT INSTANTANÉ

Imprimé le: 2019/03/13 09:47

Biochimistes cliniques: K.Benkirane, V.De Guire, M.-E.Gingras, A.Lagana-Teyssier, M.Provençal, R.Robitaille
Hématologues: Drs I.Ahmad, N.Bambace, J.Beaudet, D.Bélanger, R.Bélanger, J.Bergeron, L.Bernard, L.Busque, S.Cohen, J.S.Delisle, I.Fleury, J.Hébert, J.Kassis, T.Kiss, S.Lachance, R.LeBlanc, C.Letendre, F.Letendre, L.Mollica, J.Noujaim, C.Perreault, D-C.Roy, J-L Dionne, J.Roy, G.Sauvageau, J.St-Louis
Microbiologistes: Drs C.Béliveau, A.Couture-Cossette, S.Dufresne, Y.Émond, A.-C.Labbé, C.Lavallée, X.Marchand-Sénécal, L.Poirier
Document confidentiel. Si vous avez reçu cette copie par erreur, SVP nous aviser.

CIUSSS de l'Est-de-l'Ile-de-Montréal
Hôpital Maisonneuve-Rosemont
5415 boul. de l'Assomption, Montréal (Québec), H1T 2M4
Biochimie, Diagnostic moléculaire, Hématologie, Microbiologie, Thérapie cellulaire

FINAL
#Accès: 80122233

Projet de IRE1a dans l'obésité
Chambre:
LABO, LABORATOIRE
#Permis:

Tél:(   )   -   Fax: (   )   -

RAMQ :
Nom : **6169**
Prénom : **RECHERCHE**
Nais : 1900/01/01   Sexe: N
Adresse:
Tél :

Dossier: **T000653689**

B I O - E N D O C R I N O L O G I E

ANALYSE(S)	RESULTAT(S)	ALARME	VALEURS REF	UNITES	SIGN.

spécimen prélevé 18/09/06 10:04 reçu 18/12/12 10:08

**<u>CATÉCHOLAMINES PLASMATIQUES</u>**					
Tension artérielle	?				V/AUT
Adrénaline	202		couché: <450<br>debout: <600	pmol/L	MIRVA
Noradrénaline	3715		couché: <2400<br>debout: <3600	pmol/L	MIRVA
Dopamine	191		< 300	pmol/L	MIRVA

Une augmentation inférieure à 2 fois la limite supérieure des valeurs de référence peut être causée par des processus physiologiques, la prise de médicaments ou un mauvais prélèvement.

(*) _   V/AUT

Analyse(s) développée(s) et validée(s) par le département de biochimie de l'HMR (LC-MS/MS). Les résultats ne doivent pas être utilisés comme les seuls outils pour le diagnostic ou le suivi des traitements.

ROBRO

Validé par: ROBITAILLE, ROBERT

Légende: AN=Anormal H=Haut B=Bas C=Critique

RAPPORT INSTANTANÉ   Imprimé le: 2019/03/13 09:47

Biochimistes cliniques: K.Benkirane, V.De Guire, M.-E.Gingras, A.Lagana-Teyssier, M.Provençal, R.Robitaille
Hématologues: Drs I.Ahmad, N.Bambace, J.Beaudet, D.Bélanger, R.Bélanger, J.Bergeron, L.Bernard, L.Busque, S.Cohen, J.S.Delisle, I.Fleury, J.Hébert, J.Kassis, T.Kiss, S.Lachance, R.LeBlanc, C.Letendre, F.Letendre, L.Mollica, J.Noujaim, C.Perreault, D-C.Roy, J-L Dionne, J.Roy, G.Sauvageau, J.St-Louis
Microbiologistes: Drs C.Béliveau, A.Couture-Cossette, S.Dufresne, Y.Émond, A.-C.Labbé, C.Lavallée, X.Marchand-Sénécal, L.Poirier
Document confidentiel. Si vous avez reçu cette copie par erreur, SVP nous aviser.

CIUSSS de l'Est-de-l'Ile-de-Montréal
Hôpital Maisonneuve-Rosemont
5415 boul. de l'Assomption, Montréal (Québec), H1T 2M4
Biochimie, Diagnostic moléculaire, Hématologie, Microbiologie, Thérapie cellulaire

FINAL
#Accès: **80122254**

Projet de IRE1a dans l'obésité
Chambre:
LABO, LABORATOIRE
#Permis:

Tél:( ) - Fax: ( ) -

RAMQ :
Nom : **5953**
Prénom : **RECHERCHE**
Nais : 1900/01/01 Sexe: N
Adresse:

Tél :

Dossier: **T000653692**

B I O - E N D O C R I N O L O G I E

spécimen prélevé 18/06/14 10:06 reçu 18/12/12 10:08

CATÉCHOLAMINES PLASMATIQUES

ANALYSE(S)	RESULTAT(S)	ALARME	VALEURS REF	UNITES	SIGN.
Tension artérielle	?				V/AUT
Adrénaline	945		couché: <450<br>debout: <600	pmol/L	MIRVA
Noradrénaline	3140		couché: <2400<br>debout: <3600	pmol/L	MIRVA
Dopamine	:		< 300	pmol/L	MIRVA

Une augmentation inférieure à 2 fois la limite supérieure des valeurs de référence peut être causée par des processus physiologiques, la prise de médicaments ou un mauvais prélèvement. Une dilution du spécimen a été nécessaire pour effectuer notre technique puisque la quantité de spécimen était insuffisante. Le résultat de cette dilution n'est pas concluant. Analyse impossible à réaliser. (Dopamine)

(*) _ V/AUT

Analyse(s) développée(s) et validée(s) par le département de biochimie de l'HMR (LC-MS/MS). Les résultats ne doivent pas être utilisés comme les seuls outils pour le diagnostic ou le suivi des traitements.

ROBRO

Validé par: ROBITAILLE, ROBERT

Légende: AN=Anormal H=Haut B=Bas C=Critique

RAPPORT INSTANTANÉ Imprimé le: 2019/03/13 09:47

Biochimistes cliniques: K.Benkirane, V.De Guire, M.-E.Gingras, A.Lagana-Teyssier, M.Provençal, R.Robitaille
Hématologues: Drs I.Ahmad, N.Bambace, J.Beaudet, D.Bélanger, R.Bélanger, J.Bergeron, L.Bernard, L.Busque, S.Cohen, J.S.Delisle, I.Fleury, J.Hébert, J.Kassis, T.Kiss, S.Lachance, R.LeBlanc, C.Letendre, F.Letendre, L.Mollica, J.Noujaim, C.Perreault, D-C.Roy, J-L Dionne, J.Roy, G.Sauvageau, J.St-Louis
Microbiologistes: Drs C.Béliveau, A.Couture-Cossette, S.Dufresne, Y.Émond, A.-C.Labbé, C.Lavallée, X.Marchand-Sénécal, L.Poirier
Document confidentiel. Si vous avez reçu cette copie par erreur, SVP nous aviser.

CIUSSS de l'Est-de-l'Ile-de-Montréal **FINAL**
Hôpital Maisonneuve-Rosemont #Accès: **80122216**

5415 boul. de l'Assomption, Montréal (Québec), H1T 2M4
Biochimie, Diagnostic moléculaire, Hématologie, Microbiologie, Thérapie cellulaire

Projet de IRE1a dans l'obésité	RAMQ :	Dossier: **T000653687**
Chambre:	Nom : **5946**	
LABO, LABORATOIRE	Prénom : **RECHERCHE**	
#Permis:	Nais : 1900/01/01	Sexe: N
	Adresse:	
Tél:( ) - Fax: ( ) -	Tél :	

B I O - E N D O C R I N O L O G I E

ANALYSE(S)	RESULTAT(S)	ALARME	VALEURS REF	UNITES	SIGN.
**spécimen prelevé 18/06/14 10:02 reçu 18/12/12 10:08**					
**CATÉCHOLAMINES PLASMATIQUES**					
Tension artérielle	?				V/AUT
Adrénaline	278		couché: <450<br>debout: <600	pmol/L	MIRVA
Noradrénaline	11789		couché: <2400<br>debout: <3600	pmol/L	MIRVA
Dopamine	156		< 300	pmol/L	MIRVA

Une augmentation inférieure à 2 fois la limite supérieure des valeurs de référence peut être causée par des processus physi-ologiques, la prise de médicaments ou un mauvais prélèvement.

(*) _ V/AUT

Analyse(s) développée(s) et validée(s) par le département de biochimie de l'HMR (LC-MS/MS). Les résultats ne doivent pas être utilisés comme les seuls outils pour le diagnostic ou le suivi des traitements.

ROBRO

Validé par: ROBITAILLE, ROBERT

Légende: AN=Anormal H=Haut B=Bas C=Critique

RAPPORT INSTANTANÉ Imprimé le: 2019/03/13 09:48

Biochimistes cliniques: K.Benkirane, V.De Guire, M.-E.Gingras, A.Lagana-Teyssier, M.Provençal, R.Robitaille
Hématologues: Drs I.Ahmad, N.Bambace, J.Beaudet, D.Bélanger, R.Bélanger, J.Bergeron, L.Bernard, L.Busque, S.Cohen, J.S.Delisle, I.Fleury, J.Hébert, J.Kassis, T.Kiss, S.Lachance, R.LeBlanc, C.Letendre, F.Letendre, L.Mollica, J.Noujaim, C.Perreault, D-C.Roy, J-L Dionne, J.Roy, G.Sauvageau, J.St-Louis
Microbiologistes: Drs C.Béliveau, A.Couture-Cossette, S.Dufresne, Y.Émond, A.-C.Labbé, C.Lavallée, X.Marchand-Sénécal, L.Poirier
Document confidentiel. Si vous avez reçu cette copie par erreur, SVP nous aviser.

CIUSSS de l'Est-de-l'Ile-de-Montréal FINAL
Hôpital Maisonneuve-Rosemont #Accès: 80122243

5415 boul. de l'Assomption, Montréal (Québec), H1T 2M4

Biochimie, Diagnostic moléculaire, Hématologie, Microbiologie, Thérapie cellulaire

---

Projet de IRE1a dans l'obésité
Chambre:
LABO, LABORATOIRE
#Permis:

Tél:(   )   -   Fax: (   )   -

RAMQ :
Nom : **6173**
Prénom : **RECHERCHE**
Nais : 1900/01/01 Sexe: N
Adresse:

Tél :

Dossier: **T000653690**

B I O - E N D O C R I N O L O G I E

ANALYSE(S)	RESULTAT(S)	ALARME	VALEURS REF	UNITES	SIGN.

spécimen prélevé 18/09/06 10:04 reçu 18/12/12 10:08

CATÉCHOLAMINES PLASMATIQUES

Tension artérielle	?				V/AUT
Adrénaline	553		couché: <450<br>debout: <600	pmol/L	MIRVA
Noradrénaline	3921		couché: <2400<br>debout: <3600	pmol/L	MIRVA
Dopamine	117		< 300	pmol/L	MIRVA

Une augmentation inférieure à 2 fois la limite supérieure des valeurs de référence peut être causée par des processus physiologiques, la prise de médicaments ou un mauvais prélèvement.

(*) _ V/AUT

Analyse(s) développée(s) et validée(s) par le département de biochimie de l'HMR (LC-MS/MS). Les résultats ne doivent pas être utilisés comme les seuls outils pour le diagnostic ou le suivi des traitements.

ROBRO

Validé par: ROBITAILLE, ROBERT

Légende: AN=Anormal H=Haut B=Bas C=Critique

RAPPORT INSTANTANÉ Imprimé le: 2019/03/13 09:48

Biochimistes cliniques: K.Benkirane, V.De Guire, M.-E.Gingras, A.Lagana-Teyssier, M.Provençal, R.Robitaille

Hématologues: Drs I.Ahmad, N.Bambace, J.Beaudet, D.Bélanger, R.Bélanger, J.Bergeron, L.Bernard, L.Busque, S.Cohen, J.S.Delisle, I.Fleury, J.Hébert, J.Kassis, T.Kiss, S.Lachance, R.LeBlanc, C.Letendre, F.Letendre, L.Mollica, J.Noujaim, C.Perreault, D-C.Roy, J-L Dionne, J.Roy, G.Sauvageau, J.St-Louis

Microbiologistes: Drs C.Béliveau, A.Couture-Cossette, S.Dufresne, Y.Émond, A.-C.Labbé, C.Lavallée, X.Marchand-Sénécal, L.Poirier

Document confidentiel. Si vous avez reçu cette copie par erreur, SVP nous aviser.
