# Supplementary material for: Adipocytes influence choroidal neovascularization via PRDM16
Source: EMBO Mol Med. 2026 May 19;18(6):2379–403. doi: 10.1038/s44321-026-00441-5 (PMC13269495; doi:10.1038/s44321-026-00441-5)
Supplement: Supplementary file 7 — Figure EV4 Source Data [file 44321_2026_441_MOESM7_ESM.zip › Figure EV4/FigEV4D-F/SoftPrint_20191216_2.pdf]

CIUSSS de l'Est-de-l'Ile-de-Montréal
Hôpital Maisonneuve-Rosemont
5415 boul. de l'Assomption, Montréal (Québec), H1T 2M4
Biochimie, Diagnostic moléculaire, Hématologie, Microbiologie, Thérapie cellulaire

FINAL
#Accès: **92103841**

Projet de IRE1a dans l'obésité
Chambre:
LABO, LABORATOIRE
#Permis:

Tél:( ) - Fax: ( ) -

RAMQ :
Nom : **6870**
Prénom : **RECHERCHE**
Nais : 1900/01/01 Sexe: N
Adresse:
Tél :

Dossier: **T000740852**

BIO-ENDOCRINOLOGIE

spécimen prélevé 19/12/10 14:04 reçu 19/12/10 14:07

ANALYSE(S)	RESULTAT(S)	ALARME	VALEURS REF	UNITES	SIGN.
**CATÉCHOLAMINES PLASMATIQUES**					
Tension artérielle	?				V/AUT
Adrénaline	2746		couché: <450<br>debout: <600	pmol/L	COSCH
Noradrénaline	44489		couché: <2400<br>debout: <3600	pmol/L	COSCH
Dopamine	4972		< 300	pmol/L	COSCH

Une augmentation inférieure à 2 fois la limite supérieure des valeurs de référence peut être causée par des processus physiologiques, la prise de médicaments ou un mauvais prélèvement.

(*) _ V/AUT

Analyse(s) développée(s) et validée(s) par le département de biochimie de l'HMR (LC-MS/MS). Les résultats ne doivent pas être utilisés comme les seuls outils pour le diagnostic ou le suivi des traitements.

MEG

Validé par: GINGRAS, MARIE-EVE

Légende: AN=Anormal H=Haut B=Bas C=Critique

RAPPORT INSTANTANÉ

Imprimé le: 2019/12/16 13:36

Biochimistes cliniques: K.Benkirane, V.De Guire, M.-E.Gingras, A.Lagana-Teyssier, M.Provençal, R.Robitaille
Hématologues: Drs I.Ahmad, N.Bambace, J.Beaudet, D.Bélanger, R.Bélanger, J.Bergeron, L.Bernard, L.Busque, S.Cohen, J.S.Delisle, I.Fleury, J.Hébert, J.Kassis, T.Kiss, S.Lachance, R.LeBlanc, C.Letendre, F.Letendre, L.Mollica, J.Noujaim, C.Perreault, D-C.Roy, J-L Dionne, J.Roy, G.Sauvageau, J.St-Louis
Microbiologistes: Drs C.Béliveau, A.Couture-Cossette, S.Dufresne, Y.Émond, A.-C.Labbé, C.Lavallée, X.Marchand-Sénécal, L.Poirier
Document confidentiel. Si vous avez reçu cette copie par erreur, SVP nous aviser.
