# Supplementary material for: Adipocytes influence choroidal neovascularization via PRDM16
Source: EMBO Mol Med. 2026 May 19;18(6):2379–403. doi: 10.1038/s44321-026-00441-5 (PMC13269495; doi:10.1038/s44321-026-00441-5)
Supplement: Supplementary file 8 — Figure EV5 Source Data [file 44321_2026_441_MOESM8_ESM.zip › Figure EV5/FigEV5B/Genotyping.docx]

**Genotyping 20-07-2020**

The same samples were used for both PCRs (one sample Adipoq :Cre and one sample Adipoq:Prdm16^+/+^)

**PCR AdipoQ-cre :**

**B6 + AdipoQ AdipoQ-prdm16**


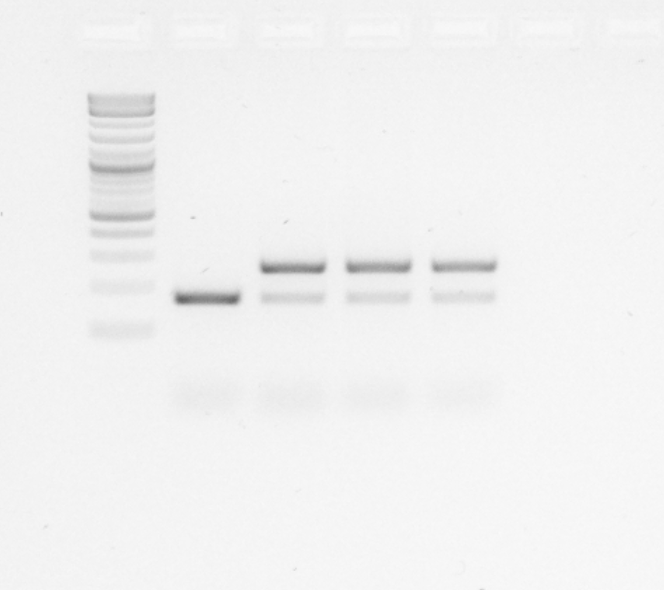


Transgene Band: 272 bp

Control Band: 175 bp

**PCR Prdm16 :**

**B6 + AdipoQ AdipoQ-prdm16**


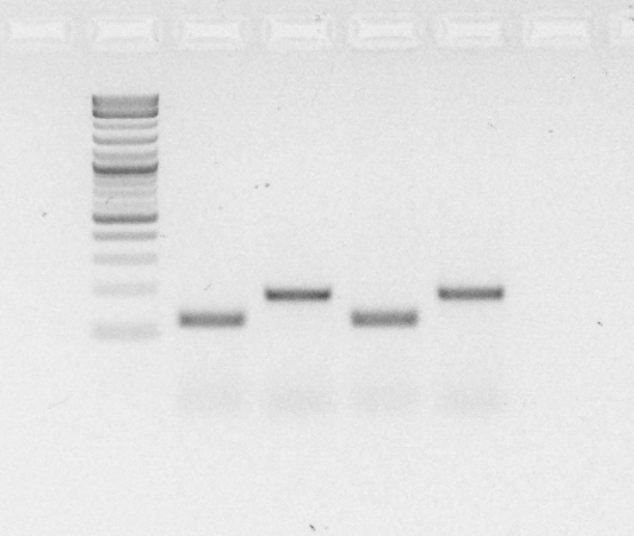


Mutant Band : 187 bp

WT Band : 124 bp

Legend
B6 (WT)
+ : Adipoq :Cre (Jackson)

AdipoQ: Adipoq:Cre

AdipoQ-prdm16: Adipoq:Prdm16^+/+^
