# Supplementary material for: Adipocytes influence choroidal neovascularization via PRDM16
Source: EMBO Mol Med. 2026 May 19;18(6):2379–403. doi: 10.1038/s44321-026-00441-5 (PMC13269495; doi:10.1038/s44321-026-00441-5)
Supplement: Supplementary file 10 — Expanded View Figures [file 44321_2026_441_MOESM10_ESM.pdf]

# Expanded View Figures

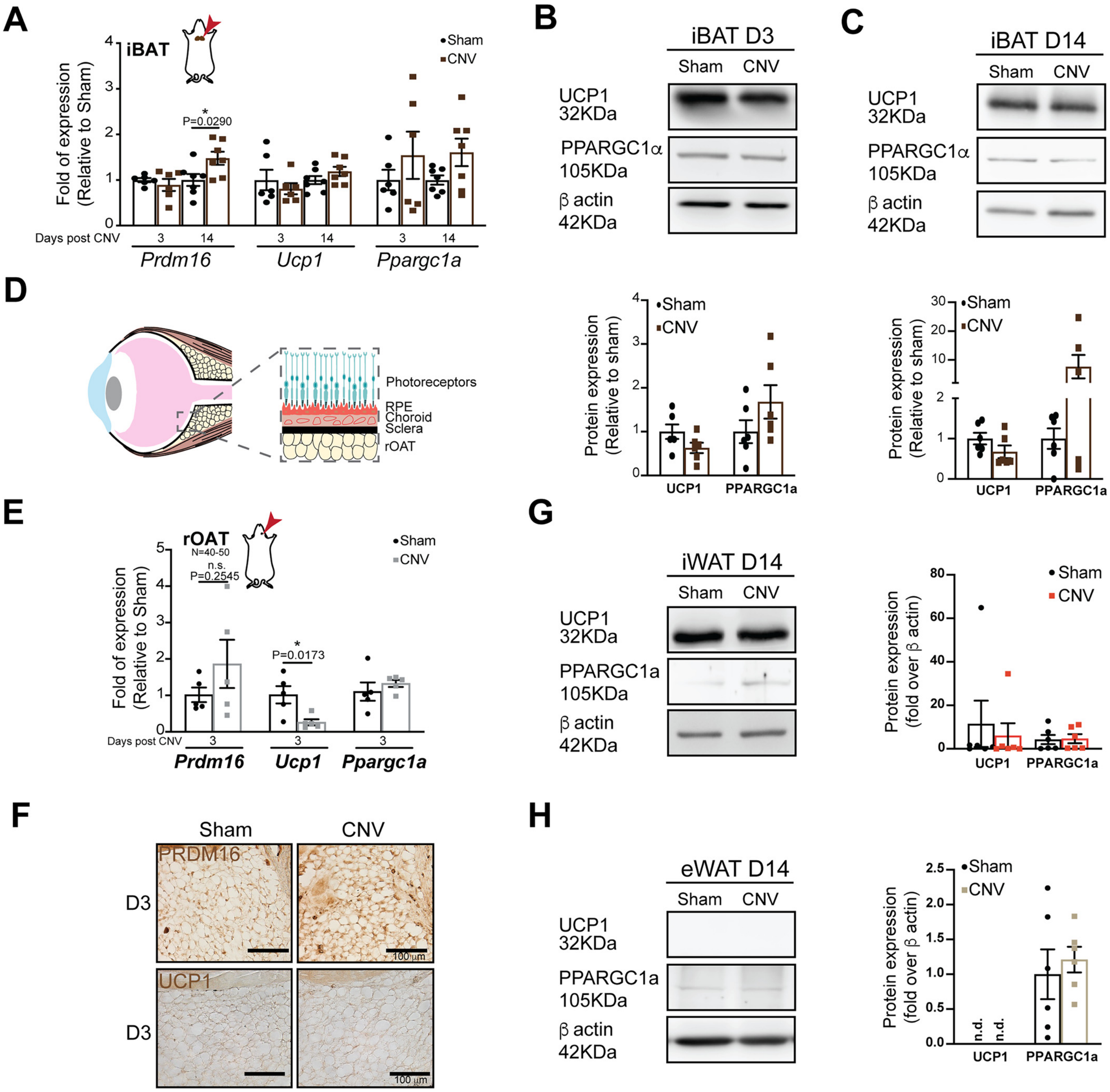


**Figure EV1. Thermogenic factor expression in iBAT is not affected by CNV.**

(**A**) iBAT relative mRNA expression of *Prdm16*, *Ucp1* and *Ppargc1a* from laser CNV and sham C57BL/6J mice 3- and 14-days post CNV, $n = 7$ mice per group. Gene expression was normalized to sham mice 3- and 14-days post CNV. (**B**) Representative immunoblots and their corresponding quantification for UCP1 and PPARGC1a protein levels of iBAT at 3-days in laser CNV and sham group ($n = 6$ per group). (**C**) Representative immunoblots and their corresponding quantification for UCP1 and PPARGC1a protein levels in iBAT 14-days in laser CNV and sham group ($n = 6$ per group). β-actin was used as a loading control. (**D**) Schematic representation of the anatomical location of rOAT. The rOAT is located around the optic nerve behind the posterior pole of the eyeball in contact with the sclera. (**E**) rOAT relative mRNA expression of *Prdm16*, *Ucp1* and *Ppargc1a* from laser CNV and sham C57BL/6J mice at 3 days post CNV. Gene expression was normalized to sham mice, $n = 40$–50 mice per group. (**F**) Representative immunohistochemistry staining images for PRDM16 and UCP1 in rOAT from laser CNV and sham C57BL/6J mice at 3 days post CNV, $n = 5$ mice per group. (**G**, **H**) Representative immunoblots and their corresponding quantification for UCP1 and PPARGC1a protein levels in (**G**) iWAT and (**H**) eWAT 14-days in laser CNV and sham group ($n = 6$ per group). β-actin was used as a loading control. Data is presented as mean ± SEM. Statistical significance was assessed using unpaired two-tailed Student's *t* test. Exact *P* values are indicated in the figure, with $^*P < 0.05$. Source data are available online for this figure.

 © The Author(s)

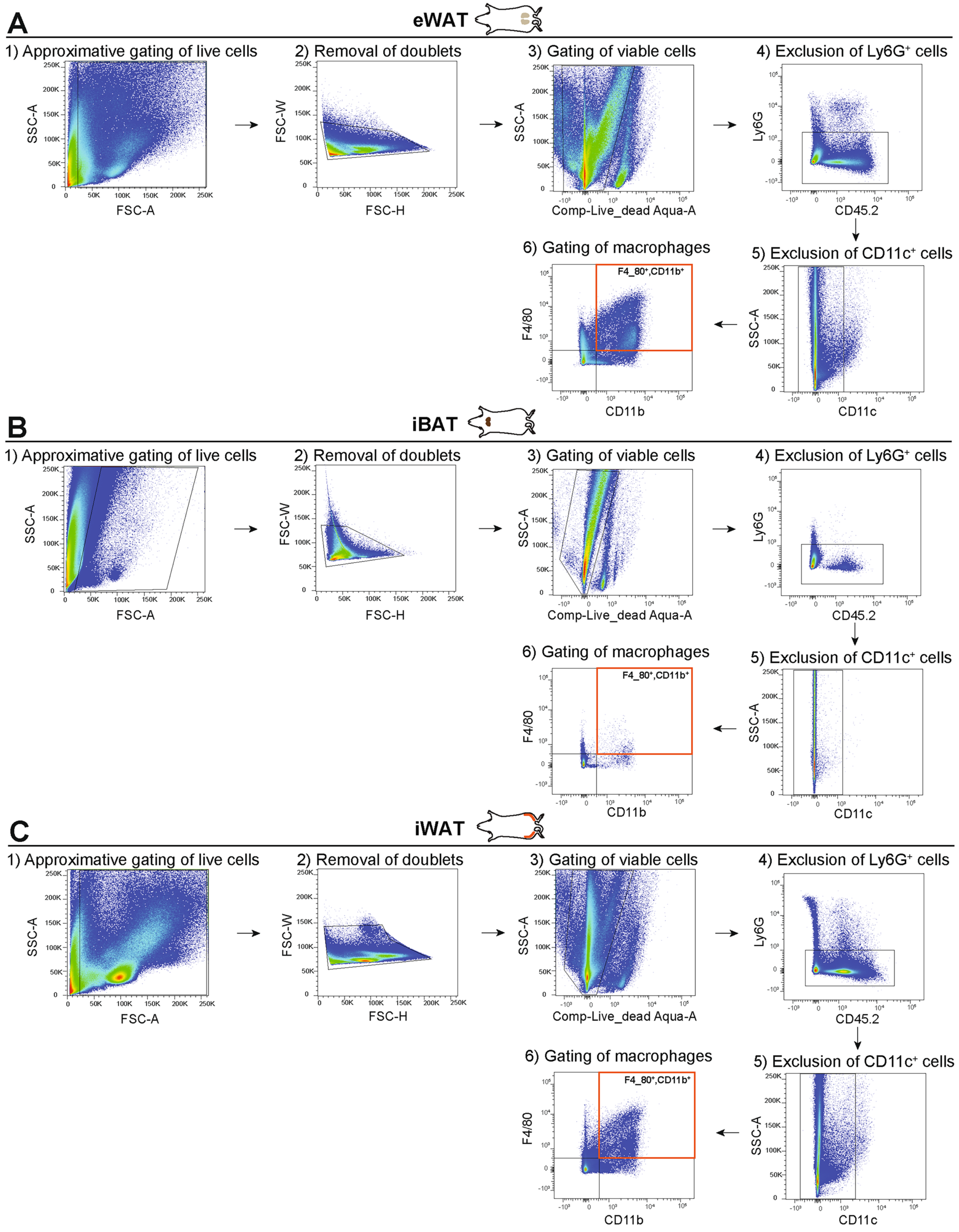
A
eWAT
1) Approximative gating of live cells
2) Removal of doublets
3) Gating of viable cells
4) Exclusion of Ly6G+ cells
5) Exclusion of CD11c+ cells
6) Gating of macrophages
F4_80+,CD11b+
SSC-A
FSC-A
FSC-W
FSC-H
Comp-Live_dead Aqua-A
Ly6G
CD45.2
F4/80
CD11b
CD11c
B
iBAT
1) Approximative gating of live cells
2) Removal of doublets
3) Gating of viable cells
4) Exclusion of Ly6G+ cells
5) Exclusion of CD11c+ cells
6) Gating of macrophages
F4_80+,CD11b+
C
iWAT
1) Approximative gating of live cells
2) Removal of doublets
3) Gating of viable cells
4) Exclusion of Ly6G+ cells
5) Exclusion of CD11c+ cells
6) Gating of macrophages
F4_80+,CD11b+

**Figure EV2. Flow cytometry gating strategies of ATMs from ATs.**

(**A–C**) Gating strategy for ATMs in the SVF of (**A**) eWAT, (**B**) iBAT, and (**C**) iWAT: (1) approximative gating of live cells, (2) gating to remove doublets, (3) selection of viable cells, (4) exclusion of Ly6G-positive cells, (5) exclusion of CD11c-positive cells, and (6) gating of ATMs.


© The Author(s)

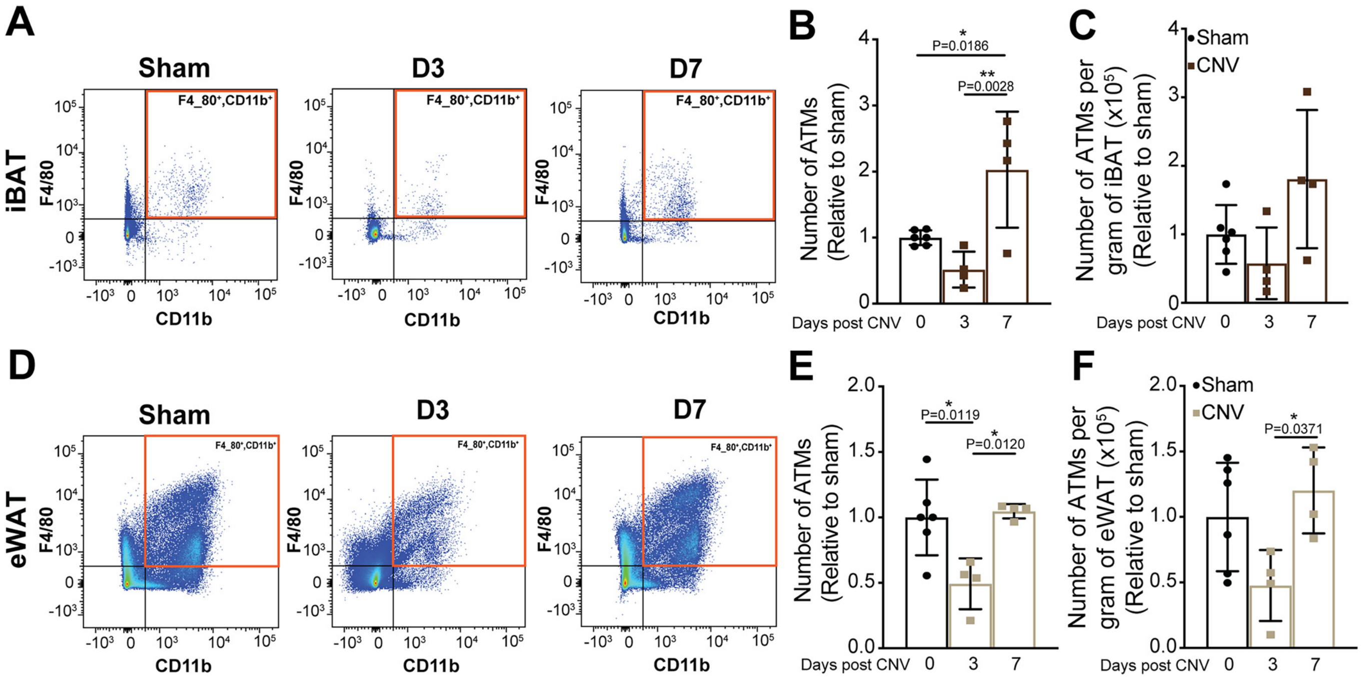


**Figure EV3. ATMs increase in iBAT during CNV formation.**

(**A**) Representative flow cytometry analysis time course of iBAT ATMs, (**B**) quantification of the number of ATMs, and (**C**) total number of ATMs per gram of iBAT normalized to sham mice from laser CNV mice at 0, 3, and 7 days after laser burn ($n = 4$–6 per group). (**D**) Representative flow cytometry analysis time course of eWAT ATMs, (**E**) quantification of the number of ATMs, and (**F**) total number of ATMs per gram of eWAT normalized to sham mice from laser CNV mice at 0, 3, and 7 days after laser burn ($n = 4$–6 per group). Data are presented as mean ± SEM. Statistical analysis was performed using ordinary one-way ANOVA followed by Tukey's multiple comparisons post hoc test. Exact $P$ values are indicated in the figure, with $^*P < 0.05$ and $^{**}P < 0.01$.

© The Author(s) 

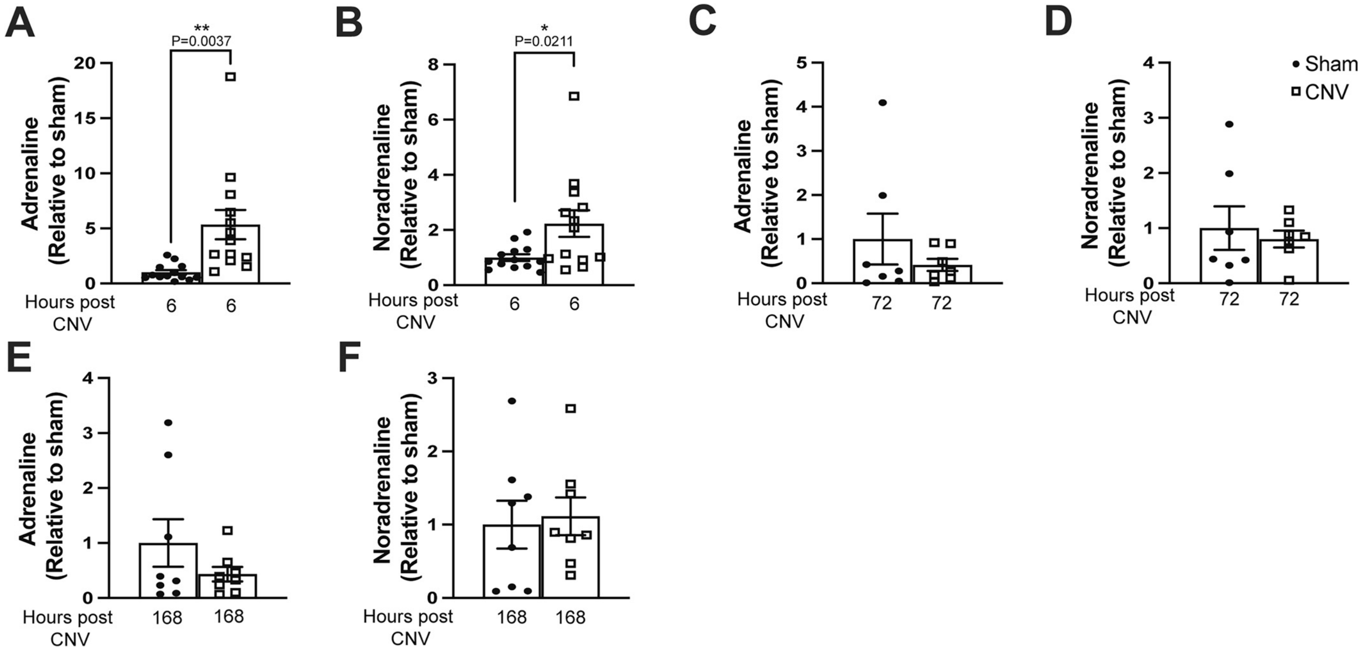


**Figure EV4. Plasma catecholamines are upregulated 6 h after laser treatment.**

(**A**) Adrenaline and (**B**) noradrenaline plasma levels from laser CNV and sham C57BL/6J mice 6 h post CNV, $n = 13$. (**C**) Adrenaline and (**D**) noradrenaline plasma levels from laser CNV and sham C57BL/6J mice 72 h post CNV, $n = 7$. (**E**) Adrenaline and (**F**) noradrenaline plasma levels from laser-burned and sham C57BL/6J mice 168 h post CNV, $n = 8$. Data are presented as mean ± SEM. Statistical significance was assessed using unpaired two-tailed Student's $t$ test. Exact $P$ values are indicated in the figure, with $^{*}P < 0.05$ and $^{**}P < 0.01$. Source data are available online for this figure.

 © The Author(s)

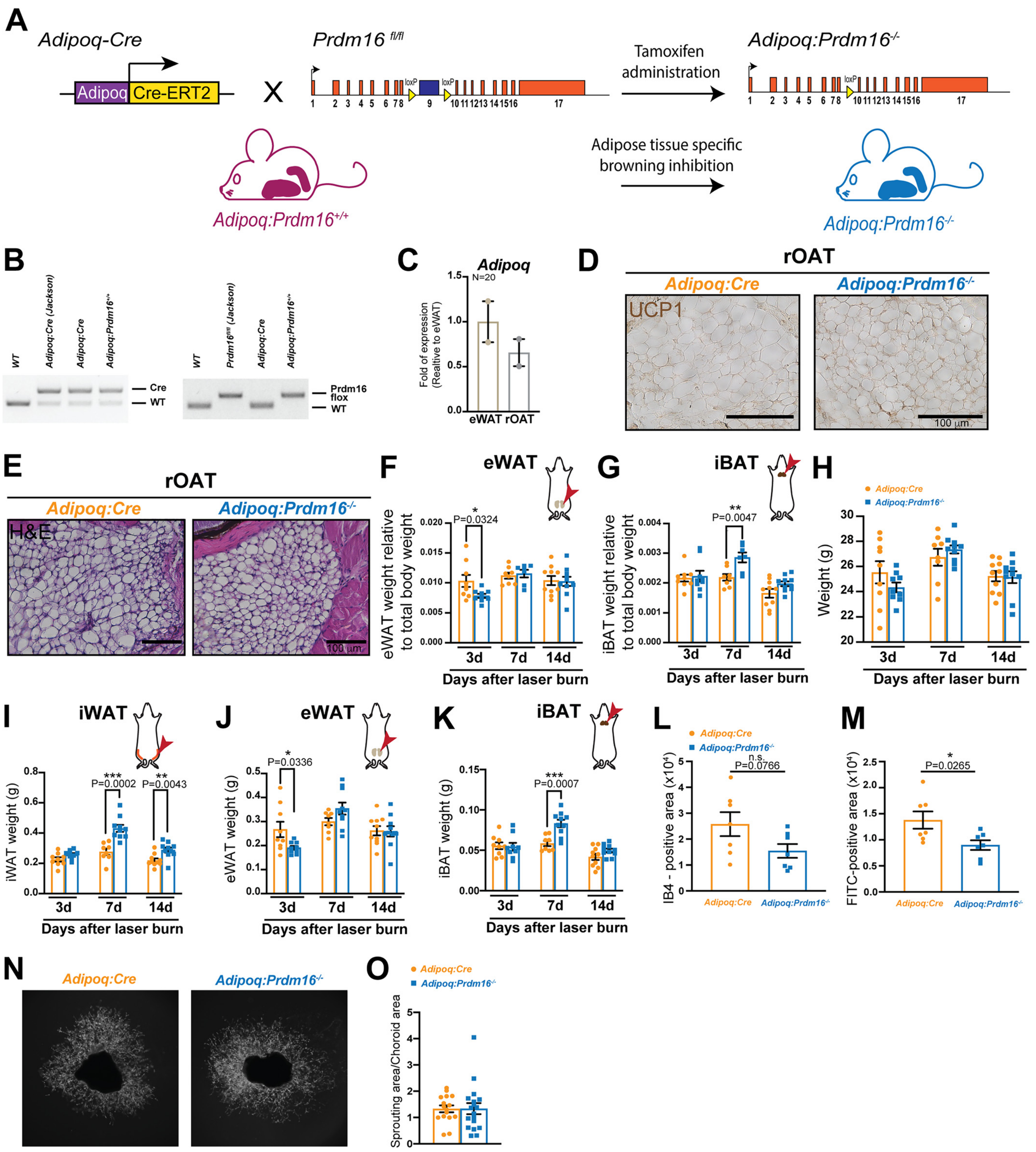
A
Adipoq-Cre
Adipoq
Cre-ERT2
X
Prdm16 fl/fl
Tamoxifen administration
Adipoq:Prdm16-/-
Adipose tissue specific browning inhibition
Adipoq:Prdm16+/+
Adipoq:Prdm16-/-
B
WT
Adipoq:Cre (Jackson)
Adipoq:Cre
Adipoq:Prdm16+/+
Cre
WT
Prdm16fl/fl (Jackson)
Prdm16 flox
C
Adipoq
N=20
Fold of expression (Realtive to eWAT)
eWAT rOAT
D
rOAT
Adipoq:Cre
Adipoq:Prdm16-/-
UCP1
100 µm
E
rOAT
Adipoq:Cre
Adipoq:Prdm16-/-
H&E
100 µm
F
eWAT
P=0.0324
eWAT weight relative to total body weight
Days after laser burn
G
iBAT
P=0.0047
iBAT weight relative to total body weight
Days after laser burn
H
Adipoq:Cre
Adipoq:Prdm16-/-
Weight (g)
Days after laser burn
I
iWAT
P=0.0002
P=0.0043
iWAT weight (g)
Days after laser burn
J
eWAT
P=0.0336
eWAT weight (g)
Days after laser burn
K
iBAT
P=0.0007
iBAT weight (g)
Days after laser burn
L
Adipoq:Cre
Adipoq:Prdm16-/-
n.s.
P=0.0766
IB4 - positive area (x10⁴)
M
P=0.0265
FITC-positive area (x10⁴)
N
Adipoq:Cre
Adipoq:Prdm16-/-
O
Adipoq:Cre
Adipoq:Prdm16-/-
Sprouting area/Choroid area

**Figure EV5. Knockout of *Prdm16* is specific to AT in *Adipoq:Prdm16*$^{+/+}$ mice.**

(**A**) Schematic representation of *Adipoq:Prdm16*$^{+/+}$ mice generation. *Adipoq:Cre* mice expressing Cre-ERT2 specifically in the ATs were crossed with *Prdm16*$^{lox/lox}$ mice that have *loxP*-flanked exon 9 of *Prdm16*, thus generating *Adipoq:Prdm16*$^{+/+}$ mice. Following tamoxifen administration, the exon 9 of *Prdm16* is removed generating *Adipoq:Prdm16*$^{-/-}$ mice that have a specific browning inhibition in the ATs from the *Adipoq:Prdm16*$^{-/-}$ mice. (**B**) Genotyping of *Adipoq:Cre* and *Adipoq:Prdm16*$^{+/+}$ mice for *Cre* and *Prdm16 loxP*-flanked regions. (**C**) eWAT and rOAT relative mRNA expression of *Adipoq* from C57BL/6J mice. Gene expression was normalized to eWAT expression, $n = 2$–20 mice per group (as per graph). (**D**, **E**) Representative immunohistochemistry staining images for (**D**) UCP1 and (**E**) H&E in rOAT from gavaged *Adipoq:Cre* and *Adipoq:Prdm16*$^{+/+}$ mice, $n = 4$ mice per group. (**F**) eWAT and (**G**) iBAT masses were calculated as a ratio from tissue-specific weight over total body weight from *Adipoq:Cre* and *Adipoq:Prdm16*$^{+/+}$ mice at 3, 7, and 14 days after laser burn ($n = 8$–10 mice per group). (**H**) Comparison of body weight from *Adipoq:Cre* and *Adipoq:Prdm16*$^{+/+}$ mice, at 3, 7, and 14 days after laser burn ($n = 8$–10 mice per group). (**I**) iWAT, (**J**) eWAT, and (**K**) iBAT masses of *Adipoq:Cre* and *Adipoq:Prdm16*$^{+/+}$ mice at 3, 7, and 14 days after laser burn ($n = 8$–10 mice per group). (**L**) Quantification of isolectin $B_4$-positive area and (**M**) quantification of FITC–dextran-labeled CNV area per laser burned *Adipoq:Cre* and *Adipoq:Prdm16*$^{+/+}$ mouse 14 days after laser burn; $n = 7$ mice per group. (**N**) Representative images of choroid explants and (**O**) quantification of sprouting area over choroid explant area from *Adipoq:Cre* ($n = 16$ explants) and *Adipoq:Prdm16*$^{+/+}$ ($n = 18$ explants) mice gavaged with tamoxifen. Data were normalized to *Adipoq:Cre* mice choroid explants. Data are presented as mean ± SEM. Statistical significance was assessed using unpaired two-tailed Student's *t* test. Exact *P* values are indicated in the figure, with $^*P < 0.05$, $^{**}P < 0.01$, and $^{***}P < 0.001$. Source data are available online for this figure.

 © The Author(s)

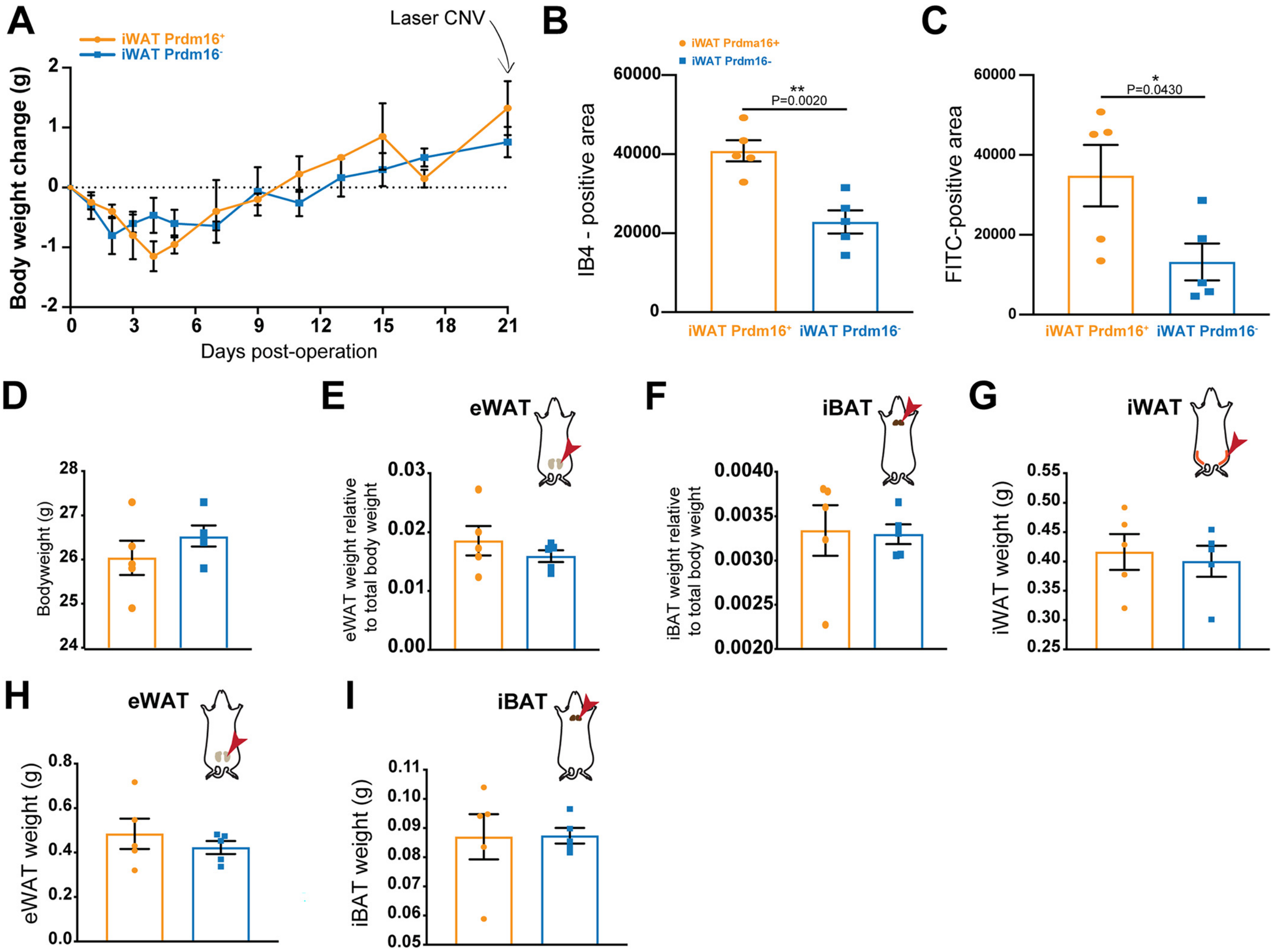


**Figure EV6. Changes in AT weight in *Adipoq:Prdm16*$^{-/-}$ mice following ATT.**

(**A**) Body weight change from *Adipoq:Prdm16*$^{-/-}$ recipient mice transplanted with either *Prdm16*-positive or *Prdm16*-negative iWAT for 21 days following the ATT ($n = 5$). (**B**) Quantification of isolectin B4-positive area and (**C**) quantification of FITC-dextran-labeled CNV area per *Adipoq:Prdm16*$^{+/+}$ mice transplanted with *Prdm16*-positive or *Prdm16*-negative iWAT 14 days after laser burn; $n = 5$ mice per group. (**D**) Comparison of the body weight of *Adipoq:Prdm16*$^{+/+}$ transplanted mice with either *Prdm16*-positive or *Prdm16*-negative iWAT. (**E**) eWAT and (**F**) iBAT masses were calculated as a ratio from tissue-specific weight over total body weight from *Adipoq:Prdm16*$^{+/+}$ mice transplanted with *Prdm16*-positive or *Prdm16*-negative iWAT at 14 days after laser burn ($n = 5$ mice per group). (**G**) iWAT, (**H**) eWAT, and (**I**) iBAT masses of *Adipoq:Prdm16*$^{+/+}$ transplanted mice with either *Prdm16*-positive or *Prdm16*-negative iWAT at 14 days after laser burn ($n = 5$ mice per group). Data are presented as mean ± SEM. Statistical significance was assessed using unpaired two-tailed Student's *t* test. Exact *P* values are indicated in the figure, with $*P < 0.05$ and $**P < 0.01$. Source data are available online for this figure.

© The Author(s)
