# Supplementary material for: Adipocytes influence choroidal neovascularization via PRDM16
Source: EMBO Mol Med. 2026 May 19;18(6):2379–403. doi: 10.1038/s44321-026-00441-5 (PMC13269495; doi:10.1038/s44321-026-00441-5)
Supplement: Supplementary file 1 — Peer Review File [file 44321_2026_441_MOESM1_ESM.pdf]

EMBO Molecular Medicine

Adipocytes influence choroidal neovascularization via PRDM16

Roberto Diaz Marin, Masayuki Hata, Vera Guber, Vincent De Guire, Ariel Wilson, Sergio Crespo-Garcia, and Przemyslaw Sapieha

Corresponding authors: Przemyslaw Sapieha (mike.sapieha@umontreal.ca) , Ariel Wilson (ariel.wilson@umontreal.ca), Sergio Crespo-Garcia (sergio.crespo.garcia@umontreal.ca)

Review Timeline:

Submission Date:	16th Mar 25
Editorial Decision:	23rd Apr 25
Revision Received:	9th Aug 25
Editorial Decision:	8th Sep 25
Revision Received:	19th Mar 26
Editorial Decision:	30th Mar 26
Revision Received:	20th Apr 26
Accepted:	23rd Apr 26

Editor: Lise Roth

Transaction Report:

(Note: With the exception of the correction of typographical or spelling errors that could be a source of ambiguity, letters and reports are not edited. Depending on transfer agreements, referee reports obtained elsewhere may or may not be included in this compilation. Referee reports are anonymous unless the Referee chooses to sign their reports.)

23rd Apr 2025

Dear Prof. Sapieha,

Thank you for submitting your manuscript to EMBO Molecular Medicine and I apologize for the delay in getting back to you as one of the referees needed more time to complete their report and I was out of the office when we received this last report. We have now heard back from the referees who have agreed to review your manuscript. As you will see below, the reviewers recognize the potential interest of the findings, but also raise a number of important concerns including - but not limited to - the use of a single model of wet AMD, the lack of mechanistic understanding of the adipocyte/neovascular connection, and the lack of demonstration of the human relevance of the Prdm16 pathway.

If you feel that you have satisfactorily addressed the points raised by the reviewers, you may wish to submit a revised version of your manuscript. Please include a covering letter detailing how you have addressed each of the points raised by the reviewers. A revised manuscript will be reviewed again and we cannot guarantee at this stage that the final outcome will be favorable.

Revising the manuscript according to the reviewers' recommendations appears to require a lot of additional work and experimentation, and given the potential interest of your findings, we are prepared to extend the deadline to 6 months.

If you would like to discuss further the points raised by the referees or your revision plan, I am available to do so via email or video. Let me know if you are interested in this option.

EMBO Molecular Medicine encourages a single round of revision only and therefore, acceptance or rejection of the manuscript will depend on the completeness of your responses included in the next, final version of the manuscript. For this reason, and to save you from any frustrations in the end, I would strongly advise against returning an incomplete revision. Should you find that the requested revisions are not feasible within the constraints outlined here and prefer, therefore, to submit your paper elsewhere, we would welcome a message to this effect.

When submitting your revised manuscript, please carefully review the instructions that follow below. We perform an initial quality control of all revised manuscripts before re-review; failure to include requested items will delay the evaluation of your revision.

1) A .docx formatted version of the manuscript text (including legends for main figures, EV figures and tables). Please make sure that the changes are highlighted to be clearly visible.

2) Individual production quality figure files as .eps, .tif, .jpg (one file per figure). For guidance, download the 'Figure Guide PDF' (https://www.embopress.org/page/journal/17574684/authorguide#figureformat).

3) At EMBO Press we ask authors to provide source data for the main figures. Our source data coordinator will contact you to discuss which figure panels we would need source data for and will also provide you with helpful tips on how to upload and organize the files.

4) A .docx formatted letter INCLUDING the reviewers' reports and your detailed point-by-point responses to their comments. As part of the EMBO Press transparent editorial process, the point-by-point response is part of the Review Process File (RPF), which will be published alongside your paper.

5) A complete author checklist, which you can download from our author guidelines (https://www.embopress.org/page/journal/17574684/authorguide#submissionofrevisions). Please insert information in the checklist that is also reflected in the manuscript. The completed author checklist will also be part of the RPF.

6) All Materials and Methods need to be described in the main text using our 'Structured Methods' format. According to this format, the Methods section includes a Reagents and Tools Table (listing key reagents, experimental models, software and relevant equipment and including their sources and relevant identifiers) followed by a Methods and Protocols section describing the methods, ideally using a step-by-step protocol format. The aim is to facilitate adoption of the methodologies across labs. Please download and fill our Reagents and Tools Table template (.docx), which you can find in our author guidelines: https://www.embopress.org/page/journal/14693178/authorguide#structuredmethods.
When submitting your revised manuscript, please do not include the Reagents and Tools Table in the Methods section of the manuscript but upload it as a separate file choosing the file type "Reagent Table".

7) Please note that all corresponding authors are required to supply an ORCID ID for their name upon submission of a revised manuscript.

8) It is mandatory to include a 'Data Availability' section after the Materials and Methods. Before submitting your revision, primary

datasets produced in this study need to be deposited in an appropriate public database, and the accession numbers and database listed under 'Data Availability'. Please remember to provide a reviewer password if the datasets are not yet public (see https://www.embopress.org/page/journal/17574684/authorguide#dataavailability).

In case you have no data that requires deposition in a public database, please state so in this section. Note that the Data Availability Section is restricted to new primary data that are part of this study.

9) For data quantification: please specify the name of the statistical test used to generate error bars and P values, the number (n) of independent experiments (specify technical or biological replicates) underlying each data point and the test used to calculate p-values in each figure legend. The figure legends should contain a basic description of n, P and the test applied. Graphs must include a description of the bars and the error bars (s.d., s.e.m.). Please provide exact p values.

10) Our journal encourages inclusion of *data citations in the reference list* to directly cite datasets that were re-used and obtained from public databases. Data citations in the article text are distinct from normal bibliographical citations and should directly link to the database records from which the data can be accessed. In the main text, data citations are formatted as follows: "Data ref: Smith et al, 2001" or "Data ref: NCBI Sequence Read Archive PRJNA342805, 2017". In the Reference list, data citations must be labeled with "[DATASET]". A data reference must provide the database name, accession number/identifiers and a resolvable link to the landing page from which the data can be accessed at the end of the reference. Further instructions are available at .

11) We replaced Supplementary Information with Expanded View (EV) Figures and Tables that are collapsible/expandable online. EV Figures should be cited as 'Figure EV1, Figure EV2" etc... in the text and their respective legends should be included in the main text after the legends of regular figures.
- For the figures that you do NOT wish to display as Expanded View figures, they should be bundled together with their legends in a single PDF file called *Appendix*, which should start with a short Table of Content. Appendix figures should be referred to in the main text as: "Appendix Figure S1, Appendix Figure S2" etc.
- Additional Tables/Datasets should be labeled and referred to as Table EV1, Dataset EV1, etc. Legends have to be provided in a separate tab in case of .xls files. Alternatively, the legend can be supplied as a separate text file (README) and zipped together with the Table/Dataset file.
See detailed instructions here:

12) The paper explained: EMBO Molecular Medicine articles are accompanied by a summary of the articles to emphasize the major findings in the paper and their medical implications for the non-specialist reader. Please provide a draft summary of your article highlighting
- the medical issue you are addressing,
- the results obtained and
- their clinical impact.
This may be edited to ensure that readers understand the significance and context of the research. Please refer to any of our published articles for an example.

13) Author contributions: CRediT has replaced the traditional author contributions section because it offers a systematic machine readable author contributions format that allows for more effective research assessment. Please remove the Authors Contributions from the manuscript and use the free text boxes beneath each contributing author's name in our system to add specific details on the author's contribution. More information is available in our guide to authors.

14) Disclosure statement and competing interests: We updated our journal's competing interests policy in January 2022 and request authors to consider both actual and perceived competing interests. Please review the policy https://www.embopress.org/competing-interests and update your competing interests if necessary.

15) Every published paper now includes a 'Synopsis' to further enhance discoverability. Synopses are displayed on the journal webpage and are freely accessible to all readers. They include a short stand first (maximum of 300 characters, including space) as well as 2-5 one-sentences bullet points that summarizes the paper. Please write the bullet points to summarize the key NEW findings. They should be designed to be complementary to the abstract - i.e. not repeat the same text. We encourage inclusion of key acronyms and quantitative information (maximum of 30 words / bullet point). Please use the passive voice. Please attach these in a separate file or send them by email, we will incorporate them accordingly.

Please also suggest a visual abstract to illustrate your article as a PNG file 550 px wide x 300-600 px high. A cropped portion of this image will serve as thumbnail for the table of content on our webpage.

16) As part of the EMBO Publications transparent editorial process initiative (see our Editorial at http://embomolmed.embopress.org/content/2/9/329), EMBO Molecular Medicine will publish online a Review Process File (RPF) to accompany accepted manuscripts.
In the event of acceptance, this file will be published in conjunction with your paper and will include the anonymous referee

reports, your point-by-point response and all pertinent correspondence relating to the manuscript. Let us know whether you agree with the publication of the RPF and as here, if you want to remove or not any figures from it prior to publication. Please note that the Authors checklist will be published at the end of the RPF.

EMBO Molecular Medicine has a "scooping protection" policy, whereby similar findings that are published by others during review or revision are not a criterion for rejection. Should you decide to submit a revised version, I do ask that you get in touch after three months if you have not completed it, to update us on the status.

I look forward to receiving your revised manuscript.

Yours sincerely,

Lise Roth

Lise Roth, PhD
Senior Editor
EMBO Molecular Medicine

***** Reviewer's comments *****

Referee #1 (Remarks for Author):

The present study from Sapieha's lab investigated the relationship between fat browning and choroidal neovascularization. The authors show laser burns in the eyeballs provoke the release of systemic catecholamines, likely leading to inflammation in distant fat tissues. Using adipocyte-specific Prdm16 knockout mice, the authors show that adipose tissue browning increases CNV, likely through the action of a circulating factor. This paper is a valuable contribution to the authors' series of studies on inter-organ communication involved in ocular neovascularization. This reviewer finds this paper interesting and valuable to this field but considers it somewhat descriptive. Before publication in EMM, mechanistic insight appears to be necessary as described below:

One major question still remains; What molecule(s) link adipocytes and ocular neovessels after all? Is it an adipocyte-derived metabolite or an adipokine? In this reviewer's opinion, the authors should attempt to identify it using the co-culture system in Figure 4G.

(Running title and Abstract)
The statements "Prdm16-expressing adipocytes contribute to CNV" and "PRDM16+-adipocytes influence choroidal neovascularization" are not formerly supported, as the data does not include cell depletion experiments. It should be correctly stated that "Prdm16 expression in adipocytes influences choroidal neovascularization".

(Figure 3H)
An increase in inflammatory cytokines may lead to the recruitment of macrophages, which could enhance pathological angiogenesis as demonstrated in lots of past studies. How do the authors reconcile this paradox?

(Figure 4D)
Although these images are angiograms, most of FITC-dextran does not appear to merge with IB4.

Referee #2 (Remarks for Author):

In this manuscript, Diaz-Marin and associates investigated the role of adipose PRDM16 in choroidal neovascularization (CNV) development associated with age-related macular degeneration (AMD). Using a laser-induced CNV mouse model, they demonstrated that selective deletion of browning-associated transcription factor Prdm16 in adipocytes inhibited adipose tissue browning and reduced CNV and conversely, reintroducing Prdm16-expressing adipose tissue aggravated CNV in Prdm16-deficient mice. These interesting findings suggest an obesity-related pathway in the pathogenesis of CNV, a hallmark of wet AMD. I have several questions regarding the possible correlation between the browning factor and the animal model employed in this study.

1. The study used a laser-induced CNV model to investigate the role and pathway related to adipose tissue browning on ocular angiogenesis. This model relies on the laser injury for the induction of abnormal angiogenesis, and is thus not reflecting the natural process and signaling pathways occurring in the RPE and choroidal tissues during AMD development. Furthermore, previous studies have shown that AT browning is induced in other conditions such as severe burns (refs 39 and 40). Thus, it is unclear whether the effect of AT browning pathway on CNV is related to laser injury. Additional models of wet AMD should be used to confirm the implication of this pathway in CNV formation.
2. How is the Prdm16 pathway and AT browning implicated in human AMD patients? Are there any genetic variants related to Prdm16 or UCP1 or other AT browning genes identified in genetic or genomic studies of AMD? Are the levels of catecholamines changed in patients with wet AMD or during the transition from intermediate to wet AMD?
3. It is unclear why iWAT is specifically sensitive to AT browning during CNV development? Is this finding supported by previous studies? There are also questions regarding the differential changes in AT browning in iWAT, iBAT, and rOAT after laser injury. The global KO of Prdm16 may therefore be inappropriate for studying this situation. Is there a specific model that can target iWAT browning?
4. It is unclear whether ATT of browning iWAT can induce choroidal or retinal angiogenesis without laser-induced CNV in vivo.
5. The in vitro study demonstrating that secreted factors from Prdm16-expressing adipocytes increase choroidal explant sprouting is interesting. However, no further study has been done to delineate what factors mediate the pro-angiogeneic effect of the adipocytes.
6. Supplemental Figure 5: the choroidal explants were isolated from tamoxifen-treated Adipoq:cre and Adipoq:Prdm16-/- animals. Since both groups of animals were treated with tamoxifen, the results would not differentiate whether tamoxifen itself had an inhibitory effect on angiogenesis. Rather, it can be studied by comparison of vessel growth from choroidal explants from tamoxifen-treated and vehicle-treated animals.

Referee #3 (Remarks for Author):

Diaz-Marin et al show that laser-induced CNV causes a selective remodeling of distal iWAT by potentially increasing content of beige-like adipocytes. They provide evidence that the deletion of PRDM16 specifically in AT decreases CNV, whereas reintroduction of PRDM16-positive adipose tissue increases CNV in a nAMD experimental model. PRDM16 expression in adipocytes is sufficient to trigger neovascularization in choroidal explants in vitro via soluble factor secretion. Their findings suggest that increased AT browning aggravates CNV. They convincingly describe the existence of a bidirectional AT-retina axis that implicates AT browning and AT PRDM16 BgAT activation in CNV formation and implications for CNV.
I would like to commend the authors on a very elegant study that emphasizes the importance of inter-organ communication in the pathogenesis of eye conditions such as CNV. An area that they have been leaders in for quite some time.
Points for their consideration:

1. The authors state that the levels of adrenaline and noradrenaline returned to baseline 3- or 7-days post CNV (Supplemental Figure S4C-F- impossible to see as something wrong on the conversion). This seems very protracted. Serum catecholamines tend to spike and resolve, how do the authors explain this protracted elevation? Is it pain that is also causing the increase in browning markers?
2. Figure 1: Measurements, or correction for comparison between samples obtained from adipose tissue, were made based on the weight of the tissue, but correction for the number of adipocytes or the volume of the sample removed, could have give different results. Please comment on this selection of analysis vs the others.
3. Figure 2: The time course for the changes in inflammatory mediators (IL-1b, IL-6, and TNF) in iWAT shows the greatest response at 14 days post CNV, whereas the eWAT shows it at 3 days post CNV even though it does not reach significance. Can the author provide an explanation? As expected, the iBAT does not show inflammation at any point examined. The retro-orbital adipose shows a trend to increase in TNF on day 3 what happens on day 14?
4. Figure 2: The data is a bit mismatched. Some of the time courses post CNV are 0, 3, &7 and others are only on day 3 while others are day 3 and day 14. Can the authors please provide the rationale for the selection of the points for that aspect of the study? It is not clear.
5. Figure 3: In the Adipog:Prdm 16-/- mice, how do the authors reconcile the increase in inflammatory cytokines in the posterior pole at day 3 and a reduction in VEGF at day 7?

Response to reviewers: Diaz-Marin *et al* Adipocytes influences choroidal neovascularization via PRDM16.

Referee #1

The present study from Sapieha's lab investigated the relationship between fat browning and choroidal neovascularization. The authors show laser burns in the eyeballs provoke the release of systemic catecholamines, likely leading to inflammation in distant fat tissues. Using adipocyte-specific Prdm16 knockout mice, the authors show that adipose tissue browning increases CNV, likely through the action of a circulating factor. This paper is a valuable contribution to the authors' series of studies on inter-organ communication involved in ocular neovascularization. This reviewer finds this paper interesting and valuable to this field but considers it somewhat descriptive. Before publication in EMM, mechanistic insight appears to be necessary as described below:

R1, Question 1: One major question still remains; What molecule(s) link adipocytes and ocular neovessels after all? Is it an adipocyte-derived metabolite or an adipokine? In this reviewer's opinion, the authors should attempt to identify it using the co-culture system in Figure 4G.

R1, answer 1: We would like to thank the reviewer for their constructive and positive assessment of our study. We agree with the reviewer that identifying the effectors of the observed adipose-retinal axis would be the next logical step. While we provide evidence for heat-labile secreted factor(s) produced by adipocytes (Figure 4G to J), they are not identified. Although of interest for our future research programs, this investigation would require fractionation followed by proteomics and lipidomic mass-spectrometry, and in vivo validation via abrogation, hence a new project altogether. We would also likely find a significant contribution of both VEGF and adiponectin, known to be involved in these processes and linked to adipose tissue secretomes. We have therefore added the following sentence to the discussion: "Potential adipose-derived factors secreted during AT browning known to influence angiogenesis (Leung et al., 1989; Ouchi et al., 2004) include VEGF and adiponectin (Wang et al., 2015).

R1, Question 2: The statements "Prdm16-expressing adipocytes contribute to CNV" and "PRDM16+-adipocytes influence choroidal neovascularization" are not formerly supported, as the data does not include cell depletion experiments. It should be correctly stated that "Prdm16 expression in adipocytes influences choroidal neovascularization".

R1, answer 2: We thank the reviewer for this suggestion. We have now revised the title to: "**Adipocytes influence choroidal neovascularization via PRDM16**"

(Figure 3H) **R1, Question 3:** An increase in inflammatory cytokines may lead to the recruitment of macrophages, which could enhance pathological angiogenesis as demonstrated in lots of past studies. How do the authors reconcile this paradox?

R1, answer 3: We thank the reviewer for this question and have now added this statement to the manuscript: "While inflammatory cytokines recruit mononuclear phagocytes (MNPs), data from other groups and our own have shown that inflammatory MNPs (simplistically M1) can reduce, limit or prune CNV ((Andriessen et al., 2021), Zandi et al., 2015)."

Literature suggests that macrophages are involved in mediating CNV (Espinosa-Heidmann et al., 2003; Sakurai et al., 2003). Macrophages detected in human and experimental CNV samples are heterogeneous cells of distinct origins, with subset-specific cell surface receptors and transcriptomic signatures (Droho et al., 2020). Within these subsets are they can be simplistically binned into classically activated pro-inflammatory M1 macrophages and alternatively activated pro-angiogenic M2 macrophages (Kauppinen et al., 2016). The laser-induced CNV mouse model mimics many aspects of pathological CNV in humans including its characteristic early upregulation of M1-related markers, followed by a secondary upregulation of M2-related markers (Xu et al., 2023; Yang et al., 2016). The M1/M2 ratio changes during CNV progression, as macrophage polarization shifts towards the pro-angiogenic M2 phenotype.

Several studies (including our own) have shown that pro-angiogenic macrophages promote CNV (Andriessen et al., 2021; Droho et al., 2023; Xu et al., 2019; Zandi et al., 2015; Zhang et al., 2018). In a previous study, where pro-angiogenic macrophages were depleted, **we also detected significant increases in pro-inflammatory cytokines** (Il1b and Il6) early on (D3 post laser-CNV), and yet observed subsequent reduced CNV size at D14 (Andriessen et al., 2021). This finding is similar to that presented in our current study, where the Adipoq:Prmdm16$^{-/-}$ mice exhibit increased Il1b and Il6 cytokines at 3 days post laser, and decreased CNV at D14 (Figure 3 F-H). These early increases in pro-inflammatory cytokines such as Il-1b and IL-6 may be contributing to delaying the M1 shift towards M2 and thus transiently maintaining an M1/M2 balance that is less conducive to CNV formation (Zandi et al., 2015).

(Figure 4D) **R1, Question 4:** Although these images are angiograms, most of FITC-dextran does not appear to merge with IB4.

R1, answer 4: As noted by the reviewer, both the FITC-dextran and IB_4 were used to label blood vessels. FITC-dextran is a high-molecular-weight molecule that we injected intracardially. Consequently, it remains confined to perfused blood vessels (Lambert et al., 2013; Shah et al., 2015). In contrast, IB_4 was applied post-mortem once the tissue was fixed in PFA 4%. IB_4 binds specifically to glycoproteins present in endothelial cells, as well as fibrotic and damaged cells within the burn area. IB_4 is therefore used to establish burn area,

and as a denominator over which neovessel perfusion is normalized. We explain in the results section '**Inhibition of pathways involved in adipose tissue browning mitigates laser-induced CNV**'

Combined, both markers provide a more comprehensive assessment of the CNV (Jawad et al., 2013; Shah et al., 2015). These differences explain why the signals from both markers do not fully overlap. We have used them to highlight different features of blood vessels in CNV and following previously stablished protocols in our lab (Andriessen et al., 2021; Andriessen et al., 2016; Hata et al., 2023; Menard et al., 2020).

Referee #2

In this manuscript, Diaz-Marin and associates investigated the role of adipose PRDM16 in choroidal neovascularization (CNV) development associated with age-related macular degeneration (AMD). Using a laser-induced CNV mouse model, they demonstrated that selective deletion of browning-associated transcription factor Prdm16 in adipocytes inhibited adipose tissue browning and reduced CNV and conversely, reintroducing Prdm16-expressing adipose tissue aggravated CNV in Prdm16-deficient mice. These interesting findings suggest an obesity-related pathway in the pathogenesis of CNV, a hallmark of wet AMD. I have several questions regarding the possible correlation between the browning factor and the animal model employed in this study.

R2, Question 1: The study used a laser-induced CNV model to investigate the role and pathway related to adipose tissue browning on ocular angiogenesis. This model relies on the laser injury for the induction of abnormal angiogenesis, and is thus not reflecting the natural process and signaling pathways occurring in the RPE and choroidal tissues during AMD development. Furthermore, previous studies have shown that AT browning is induced in other conditions such as severe burns (refs 39 and 40). Thus, it is unclear whether the effect of AT browning pathway on CNV is related to laser injury. Additional models of wet AMD should be used to confirm the implication of this pathway in CNV formation.

R2, answer 1: We thank the reviewer for this important comment. We agree that laser-induced CNV has limitations, yet recapitulates aspects of human disease with regards to inflammation-driven neoangiogeneiss of the choroid. The mode is however driven by an acute injury and does not fully recapitulate the progressive age-related pathophysiology of neovascular AMD in humans. This model, however, remains the gold standard for *in vivo* studies addressing choroidal neovascularization due to: 1) its high reproducibility, 2) the well-defined CNV timeline, and 3) the suitability of the model for genetic and pharmaceutical manipulations (Lambert et al., 2013; Shah et al., 2015). In contrast, alternative nAMD models, such as transgenic mice overexpressing VEGFA, or aged mice with spontaneous CNV, suffer from poor reproducibility, low lesion incidence and significant variability (Grossniklaus et al., 2010). Another transgenic mouse strain, JR5558, which exhibits spontaneous CNV with a frequency of 1:10 at best, is unreliable and is therefore no longer in use for screening by

pharma. For these reasons, these models can be suboptimal for testing systemic interventions, as often other organs might be also affected (i.e. overexpression of VEGF is not eye-specific). Thus, when studying the inter-organ communication of the adipose tissue with the eye, we considered that the laser-induced CNV model remained the best choice.

Thus, while we acknowledge the limitations of the laser-induced CNV model, its technical advantages combined with the non-injury-based assay support the robustness and relevance of our conclusions. We added the following sentence to the results section, following the description of the laser-induced CNV model: While the laser CNV model does not recapitulate the protracted age-dependant nature of human neovascular AMD, it models aspects of human disease with regards to inflammation-driven neoangiogeneiss of the choroid. It was selected for its superior reproducibility and lesion incidence compared to transgenic or aging mouse strains that exhibit spontaneous CNVs (Grossniklaus et al., 2010).

Importantly, to complement and validate our findings, we also employed an *ex vivo* choroidal sprouting assay, which does not rely on laser injury (Figure 4). In this ex vivo system, conditioned media from *Prdm16*-positive adipocytes significantly enhanced choroidal sprouting, supporting the idea that a circulating factor secreted by adipocytes can elicit neovascularization in the choroid. We added this clarification to the results section “Mice were not subjected to laser CNV to decouple angiogenesis that may result from distal burns, which have been shown to induce AT browning (Patsouris et al., 2015; Sidossis et al., 2015).” to support the sentence “We investigated whether secreted factors from AT undergoing browning could influence angiogenesis” .

2) How is the Prdm16 pathway and AT browning implicated in human AMD patients? Are there any genetic variants related to Prdm16 or UCP1 or other AT browning genes identified in genetic or genomic studies of AMD? Are the levels of catecholamines changed in patients with wet AMD or during the transition from intermediate to wet AMD?

R2, answer 2: We thank the reviewer for raising insightful questions. To date, direct evidence linking the PRDM16 pathway, adipose tissue browning, genetic variants in Prdm16 or UCP1, as well as alterations in catecholamine levels to AMD pathogenesis in humans remains limited and not well defined.

While no studies have directly implicated the PRDM16 pathway in human AMD, the possibility of a link exists due to the physiological role of PRDM16 in promoting adipose tissue browning and maintaining mitochondrial function. Indeed, PRDM16 drives the thermogenic gene program in brown and beige adipocytes, which are known to exert anti-inflammatory and metabolically protective effects (Seale et al., 2007). The loss of this thermogenic capacity occurs in obesity and aging, and has been associated with increased systemic inflammation, a known contributor to AMD pathogenesis (Ambati & Fowler, 2012). Recently, a study using

18F-fluorodeoxyglucose PET/CT imaging showed that patients with late AMD exhibited a significantly higher visceral adipose tissue (VAT) metabolic activity compared to non-AMD controls (Choi et al., 2023). This finding supports the hypothesis that adipose tissue inflammation and altered metabolic status are linked to AMD, suggesting that pathways regulating AT activation and browning could play a role in disease progression.

At the genetic level, large-scale genome-wide association studies (GWAS) of AMD, such as those from the International AMD Genomics Consortium (Fritsche et al., 2016), have not identified PRDM16, UCP1, or other canonical AT browning regulators as significantly associated loci.

Regarding catecholamines, there is limited direct evidence assessing catecholamine levels in AMD patients. However, it has been shown that beta-adrenergic receptor modulation can influence neovascularization in animal models of AMD (Tan et al., 2016).

3) It is unclear why iWAT is specifically sensitive to AT browning during CNV development? Is this finding supported by previous studies? There are also questions regarding the differential changes in AT browning in iWAT, iBAT, and rOAT after laser injury. The global KO of Prdm16 may therefore be inappropriate for studying this situation. Is there a specific model that can target iWAT browning?

R2, answer 3: We thank the reviewer for this comment. We do not use a global KO of PRDM16 but a Adipo-cre under the control of tamoxifen meaning only adipocytes will be devoid of PRDM16.
Beyond use transgenic mouse line, we reinforced our conclusions on the role of PRDM16 using adipose tissue transplants, therefore fortifying the observation that PRDM16 influences CNV. To our knowledge, this is the first study to systematically analyze and compare the responses of different adipose tissue depots in the context of choroidal neovascularization, highlighting the importance of regional adipose biology in inter-organ communication during ocular disease. We agree that depot-specific variability in browning (iWAT, iBAT, rOAT) is biologically significant. Our data reflects these differences and underscore the particularly strong browning response observed in iWAT. This is consistent with previous literature showing that iWAT is among the most responsive fat depots to sympathetic stimulation and β-adrenergic signaling (Bowers et al., 2004; Contreras et al., 2014; Munzberg et al., 2021; Natarajan et al., 2024). Our findings, suggest that systemic catecholamine release following laser injury preferentially activates iWAT, likely due to depot-specific differences in innervation.

It is important to note that the origin and phenotypic heterogeneity of beige adipocytes remain under investigation, and browning likely exists along a functional continuum. Currently, no Cre drivers allow for exclusive and complete targeting of beige adipocytes within specific depots. Therefore, we used an adiponectin-CreERT2 model, a tamoxifen-inducible, adipocyte-specific

knockout system to delete that Prdm16 specifically in mature adipocytes. This renders possible the selective impairment of browning in adipocytes without affecting Prdm16 function in other tissues, including the eye or immune cells (Cohen et al., 2014).

4) It is unclear whether ATT of browning iWAT can induce choroidal or retinal angiogenesis without laser-induced CNV in vivo.

R2, answer 4: Mice typically do not develop spontaneous CNV unless provoked by injury, genetic mutations or perhaps extreme age. We did not observe any additional CNVs in the mice having received ATTs beyond the four per eye that resulted from the argon laser burns. This suggests that the 5 week timespan between ATT and euthanasia (3 weeks after ATT+ 2 week following laser-burn) was not sufficient to induce spontaneous CNVs due to ATT.

5) The in vitro study demonstrating that secreted factors from Prdm16-expressing adipocytes increase choroidal explant sprouting is interesting. However, no further study has been done to delineate what factors mediate the pro-angiogeneic effect of the adipocytes.

R2, answer 5: We thank the reviewer for this question and agree with the reviewer that identifying the effectors of the observed adipose-retinal axis would be the next logical step. While we provide evidence for secreted factor(s) and heat sensitive factors produced by adipocytes (Figure 4G to J), they are not identified. Although of interest for our future research programs, this investigation would require fractionation followed by proteomics and lipidomic mass-spectrometry, and in vivo validation via abrogation, hence a new project altogether. We would also likely find a significant contribution of both VEGF and adiponectin, known to be involved in these processes and linked to adipose tissue secretomes. We have therefore added the following sentence to the discussion: Potential adipose-derived factors secreted during AT browning known to influence angiogenesis (Leung et al., 1989; Ouchi et al., 2004) include VEGF and adiponectin (Wang et al., 2015).

6) Supplemental Figure 5: the choroidal explants were isolated from tamoxifen-treated Adipoq:cre and Adipoq:Prdm16-/- animals. Since both groups of animals were treated with tamoxifen, the results would not differentiate whether tamoxifen itself had an inhibitory effect on angiogenesis. Rather, it can be studied by comparison of vessel growth from choroidal explants from tamoxifen-treated and vehicle-treated animals.

R2, answer 6: We thank the reviewer for raising this thoughtful point. We agree that tamoxifen could have a potential off-target effect on angiogenesis and this was elegantly demonstrated by the Christina Ruhrberg Lab. However, in our experimental design, both groups Adipoq:Cre (control) and Adipoq:Prdm16$^{-/-}$ were systemically treated with tamoxifen. This treatment is required to induce Cre recombinase activity in mature adipocytes and trigger Prdm16 deletion specifically in the knockout animals. This experimental set up ensures that

any observed differences in choroidal sprouting are specifically attributable to the loss of Prdm16 in adipocytes, rather than to tamoxifen exposure itself.

Referee #3

Diaz-Marin et al show that laser-induced CNV causes a selective remodeling of distal iWAT by potentially increasing content of beige-like adipocytes. They provide evidence that the deletion of PRDM16 specifically in AT decreases CNV, whereas reintroduction of PRDM16-positive adipose tissue increases CNV in a nAMD experimental model. PRDM16 expression in adipocytes is sufficient to trigger neovascularization in choroidal explants in vitro via soluble factor secretion. Their findings suggest that increased AT browning aggravates CNV. They convincingly describe the existence of a bidirectional AT-retina axis that implicates AT browning and AT PRDM16 BgAT activation in CNV formation and implications for CNV.

I would like to commend the authors on a very elegant study that emphasizes the importance of inter-organ communication in the pathogenesis of eye conditions such as CNV. An area that they have been leaders in for quite some time.

1) The authors state that the levels of adrenaline and noradrenaline returned to baseline 3- or 7-days post CNV (Supplemental Figure S4C-F- impossible to see as something wrong on the conversion). This seems very protracted. Serum catecholamines tend to spike and resolve, how do the authors explain this protracted elevation? Is it pain that is also causing the increase in browning markers?

R3, answer 1: We thank the reviewer for their kind words, thoughtful and constructive feedback. We thank the reviewer for raising this point. We apologize for the display issue in Supplemental Figure S4C-F and for the lack of clarity in the original text, which may have led to a misinterpretation regarding the duration of catecholamine elevation. We have reformatted the supplemental figure to allow proper viewing of the data. To clarify, plasma levels of adrenaline and noradrenaline were measured at specific time points: 6 hours, 3 days, and 7 days post-laser-induced CNV. We observed a significant elevation at the 6-hour time point (Supplemental Figure S4A-B), followed by a return to baseline levels at both 3 and 7 days (Supplemental Figure S4C-F). Hence, it is unlikely that a sustained pain response was at play. We did not perform continuous or serial sampling during the intervening period, and we have revised the text in the results section to clearly reflect this methodology. We have rephrased our interpretation of these results to avoid any possible confusion as follows: "We found laser-induced CNV provokes a rapid release of systemic catecholamines, significantly elevated plasma levels of adrenaline and noradrenaline 6 hours after injury (**Figure EV4A-B**). However, levels of adrenaline and noradrenaline returned to baseline 3- or 7-days post CNV (**Figure EV4C-F**). These data suggest that a rise in systemic catecholamines post-laser burn are transient and precede increase in browning markers in iWAT.'

Regarding the potential contribution of pain to the observed effects: This is a good point. Because the elevation of catecholamines is transient (normalized before 72 hours), we think it is unlikely that pain contributes to the outcome of CNV measured 14 days post burn. However, it would be interesting to study the impact of pain on immune cell recruitment and how it shapes the neovascular response.

2) Figure 1: Measurements, or correction for comparison between samples obtained from adipose tissue, were made based on the weight of the tissue, but correction for the number of adipocytes or the volume of the sample removed, could have give different results. Please comment on this selection of analysis vs the others.

R3, answer 2: We thank the reviewer for raising this point. <u>We would like to clarify that no data in Figure 1 were normalized to adipose tissue weight.</u> Specifically, gene expression levels were normalized to housekeeping gene *Tbp*, and protein levels were normalized to loading control β-actin.

We did however normalize adipose tissue macrophages (ATMs) numbers to adipose tissue weight in our FACS analyses (Figure 2G), presented alongside non-normalized counts (Figure 2F). Normalization to adipose tissue weight is a standard in metabolic studies, as this method is particularly relevant for studies in obesity or metabolic disease where adipose tissue expansion is a central feature (Carey & Camell, 2024; Cho et al., 2014; L'Homme et al., 2024; Wilson et al., 2018). In this case, we acknowledge that normalization to adipocyte number or tissue volume could provide different interpretations. However, adjusting for the number of adipocytes may introduce variability, particularly in tissues with heterogeneous adipocyte populations of varying sizes. Likewise, volume-based normalization could be more difficult to standardize across all samples due to variations in tissue digestion efficiency, extracellular matrix content, and fibrotic remodelling. To ensure transparency, we also include the raw ATM values unnormalized to adipose tissue weight allowing readers to independently evaluate the data without normalization (Figure 2F, Expanded View Figure 3B and 3E).

4) Figure 2: The time course for the changes in inflammatory mediators (IL-1b, IL-6, and TNF) in iWAT shows the greatest response at 14 days post CNV, whereas the eWAT shows it at 3 days post CNV even though it does not reach significance. Can the author provide an explanation? As expected, the iBAT does not show inflammation at any point examined. The retro-orbital adipose shows a trend to increase in TNF on day 3 what happens on day 14?

R3, answer 4: This is a good question. Our study provides evidence of different inflammatory kinetics in adipose tissue depots throughout the body. Each of these depots have their unique cell composition, innervation, and localization, which looks like it affects the dynamics of stress response. Although our study does not address the reason for the varying kinetics of inflammatory responses in each AT depot, we believe presenting these unique signatures is

indicative of an inter-organ crosstalk that would benefit from further study. (We also believe that this complexity is missed in studies solely focused on one adipose tissue depot.)

5) Figure 2: The data is a bit mismatched. Some of the time courses post CNV are 0, 3, &7 and others are only on day 3 while others are day 3 and day 14. Can the authors please provide the rationale for the selection of the points for that aspect of the study? It is not clear.

R3, answer 5: We thank the reviewer for this comment. We appreciate the opportunity to clarify our rationale, as all timepoints were selected for specific reasons.

For eWAT, iWAT and iBAT, we had preliminary evidence or literature support suggesting early (Day 3) and late (Day 14) time points would capture the most biologically relevant changes related to browning activation and inflammation. Due to the increase in inflammation that was observed in iWAT at day 14 post-CNV, we sought to determine whether macrophage recruitment to the adipose tissues was affected earlier. For this reason, we added 3 and 7-day time points.

For the RPE-choroid-sclera complex qPCR data (**Figure 3H**), we selected time points that correspond to the key phases of inflammation and neovascularization in the CNV model. Day 3 represents the peak of inflammatory cell infiltration, while 7 days is considered the peak of neovascular lesion formation (Ambati et al., 2003; Grossniklaus et al., 2010; Liu et al., 2017; Miller et al., 1990).

In the case of retro-orbital adipose tissue (rOAT), our primary interest was in studying adipose tissue remodeling related to browning. Due to the small amount of mRNA that can be extracted from two rOAT (~2,5 ng), we needed to pool samples from at least 10 different mice to obtain enough material for cDNA synthesis (500ng of cDNA required for 3 genes). Consequently, for our analysis with an n=5, we used more than 80 mice across both the browning and inflammation panels (**Figure S1E and Figure 2D**). Given the practical limitations and the fact that rOAT immunohistochemistry staining for PRDM16 and UCP1 did not reveal significant differences in rOAT browning, we opted to focus on day 3 post-CNV for our time course. This decision allowed us to maximize the biological relevance of the data while minimizing the number of animals used.

6) Figure 3: In the Adipog:Prdm 16-/- mice, how do the authors reconcile the increase in inflammatory cytokines in the posterior pole at day 3 and a reduction in VEGF at day 7?

R3, answer 6: We thank the reviewer for this question and it's an important point. We and others have noted that while subsets of mononuclear phagocytes (MNPs) that promote angiogenesis can drive CNV, inflammatory MNPs can actually suppress and prune it. We believe that this concept is at play here and added the following sentence to the results:

“While inflammatory cytokines recruit mononuclear phagocytes (MNPs), data from other groups and our own have shown that inflammatory MNPs (simplistically M1) can reduce, limit or prune CNV ((Andriessen et al., 2021) Zandi et al., 2015).”

More specifically, studies suggests that macrophages are involved in mediating CNV (Espinosa-Heidmann et al., 2003; Sakurai et al., 2003). Macrophages detected in human and experimental CNV samples are heterogeneous cells of distinct origins, with subset-specific cell surface receptors and transcriptomic signatures (Droho et al., 2020). Within these subsets are they can be simplistically binned into classically activated pro-inflammatory M1 macrophages and alternatively activated pro-angiogenic M2 macrophages (Kauppinen et al., 2016). The laser-induced CNV mouse model mimics many aspects of pathological CNV in humans including its characteristic early upregulation of M1-related markers, followed by a secondary upregulation of M2-related markers (Xu et al., 2023; Yang et al., 2016). The M1/M2 ratio changes during CNV progression, as macrophage polarization shifts towards the pro-angiogenic M2 phenotype.

Several studies (including our own) have shown that pro-angiogenic macrophages promote CNV (Andriessen et al., 2021; Droho et al., 2023; Xu et al., 2019; Zandi et al., 2015; Zhang et al., 2018). In a previous study, where pro-angiogenic macrophages were depleted, **we also detected significant increases in pro-inflammatory cytokines** (Il1b and Il6) early on (D3 post laser-CNV), and yet observed subsequent reduced CNV size at D14 (Andriessen et al., 2021). This finding is similar to that presented in our current study, where the Adipoq:Prmdm16$^{-/-}$ mice exhibit increased Il1b and Il6 cytokines at 3 days post laser, and decreased CNV at D14 (Figure 3 F-H). These early increases in pro-inflammatory cytokines such as Il-1b and IL-6 may be contributing to delaying the M1 shift towards M2 and thus transiently maintaining an M1/M2 balance that is less conducive to CNV formation (Zandi et al., 2015).

References:

Ambati, J., Anand, A., Fernandez, S., Sakurai, E., Lynn, B. C., Kuziel, W. A., Rollins, B. J., & Ambati, B. K. (2003). An animal model of age-related macular degeneration in senescent Ccl-2- or Ccr-2-deficient mice. *Nat Med*, *9*(11), 1390-1397. https://doi.org/10.1038/nm950

Ambati, J., & Fowler, B. J. (2012). Mechanisms of age-related macular degeneration. *Neuron*, *75*(1), 26-39. https://doi.org/10.1016/j.neuron.2012.06.018

Andriessen, E., Binet, F., Fournier, F., Hata, M., Dejda, A., Mawambo, G., Crespo-Garcia, S., Pilon, F., Buscarlet, M., Beauchemin, K., Bougie, V., Cumberlidge, G., Wilson, A. M., Bourgault, S., Rezende, F. A., Beaulieu, N., Delisle, J. S., & Sapieha, P. (2021). Myeloid-resident neuropilin-1 promotes choroidal neovascularization while mitigating inflammation. *EMBO Mol Med*, *13*(5), e11754. https://doi.org/10.15252/emmm.201911754

Andriessen, E. M., Wilson, A. M., Mawambo, G., Dejda, A., Miloudi, K., Sennlaub, F., & Sapieha, P. (2016). Gut microbiota influences pathological angiogenesis in obesity-driven choroidal neovascularization. *EMBO Mol Med*, *8*(12), 1366-1379. https://doi.org/10.15252/emmm.201606531

Bowers, R. R., Festuccia, W. T., Song, C. K., Shi, H., Migliorini, R. H., & Bartness, T. J. (2004). Sympathetic innervation of white adipose tissue and its regulation of fat cell number. *Am J Physiol Regul Integr Comp Physiol*, *286*(6), R1167-1175. https://doi.org/10.1152/ajpregu.00558.2003

Carey, A., & Camell, C. D. (2024). Protocol to examine murine visceral adipose tissue immune cells using fluorescence-based flow cytometry. *STAR Protoc*, *5*(3), 103227. https://doi.org/10.1016/j.xpro.2024.103227

Cho, K. W., Morris, D. L., & Lumeng, C. N. (2014). Flow cytometry analyses of adipose tissue macrophages. *Methods Enzymol*, *537*, 297-314. https://doi.org/10.1016/B978-0-12-411619-1.00016-1

Choi, K. E., Joung, C., Pahk, K. J., Kim, H., & Pahk, K. (2023). Metabolic activity of visceral adipose tissue is associated with age-related macular degeneration: a pilot (18)F-FDG PET/CT study. *Front Endocrinol (Lausanne)*, *14*, 1322326. https://doi.org/10.3389/fendo.2023.1322326

Cohen, P., Levy, J. D., Zhang, Y., Frontini, A., Kolodin, D. P., Svensson, K. J., Lo, J. C., Zeng, X., Ye, L., Khandekar, M. J., Wu, J., Gunawardana, S. C., Banks, A. S., Camporez, J. P., Jurczak, M. J., Kajimura, S., Piston, D. W., Mathis, D., Cinti, S.,…Spiegelman, B. M. (2014). Ablation of PRDM16 and beige adipose causes metabolic dysfunction and a subcutaneous to visceral fat switch. *Cell*, *156*(1-2), 304-316. https://doi.org/10.1016/j.cell.2013.12.021

Contreras, G. A., Lee, Y. H., Mottillo, E. P., & Granneman, J. G. (2014). Inducible brown adipocytes in subcutaneous inguinal white fat: the role of continuous sympathetic stimulation. *Am J Physiol Endocrinol Metab*, *307*(9), E793-799. https://doi.org/10.1152/ajpendo.00033.2014

Droho, S., Rajesh, A., Cuda, C. M., Perlman, H., & Lavine, J. A. (2023). CD11c+ macrophages are proangiogenic and necessary for experimental choroidal neovascularization. *JCI insight*, *8*(7). https://doi.org/10.1172/jci.insight.168142

Droho, S., Thomson, B. R., Makinde, H. M., Cuda, C. M., Perlman, H., & Lavine, J. A. (2020). Ocular macrophage origin and heterogeneity during steady state and experimental choroidal neovascularization. *J Neuroinflammation*, *17*(1), 341. https://doi.org/10.1186/s12974-020-02010-0

Espinosa-Heidmann, D. G., Suner, I. J., Hernandez, E. P., Monroy, D., Csaky, K. G., & Cousins, S. W. (2003). Macrophage depletion diminishes lesion size and severity in experimental choroidal neovascularization. *Invest Ophthalmol Vis Sci*, *44*(8), 3586-3592. https://doi.org/10.1167/iovs.03-0038

Fritsche, L. G., Igl, W., Bailey, J. N., Grassmann, F., Sengupta, S., Bragg-Gresham, J. L., Burdon, K. P., Hebbring, S. J., Wen, C., Gorski, M., Kim, I. K., Cho, D., Zack, D., Souied, E., Scholl, H. P., Bala, E., Lee, K. E., Hunter, D. J., Sardell, R. J.,…Heid, I. M. (2016). A large genome-wide association study of age-related macular degeneration highlights contributions of rare and common variants. *Nat Genet*, *48*(2), 134-143. https://doi.org/10.1038/ng.3448

Grossniklaus, H. E., Kang, S. J., & Berglin, L. (2010). Animal models of choroidal and retinal neovascularization. *Prog Retin Eye Res*, *29*(6), 500-519. https://doi.org/10.1016/j.preteyeres.2010.05.003

Hata, M., Andriessen, E., Hata, M., Diaz-Marin, R., Fournier, F., Crespo-Garcia, S., Blot, G., Juneau, R., Pilon, F., Dejda, A., Guber, V., Heckel, E., Daneault, C., Calderon, V., Des Rosiers, C., Melichar, H. J., Langmann, T., Joyal, J. S., Wilson, A. M., & Sapieha, P. (2023). Past history of obesity triggers persistent epigenetic changes in innate immunity and exacerbates neuroinflammation. *Science*, *379*(6627), 45-62. https://doi.org/10.1126/science.abj8894

Jawad, S., Liu, B., Li, Z., Katamay, R., Campos, M., Wei, L., Sen, H. N., Ling, D., Martinez Estrada, F., Amaral, J., Chan, C. C., Fariss, R., Gordon, S., & Nussenblatt, R. B. (2013). The role of macrophage class a scavenger receptors in a laser-induced murine choroidal neovascularization model. *Invest Ophthalmol Vis Sci*, *54*(9), 5959-5970. https://doi.org/10.1167/iovs.12-11380

Kauppinen, A., Paterno, J. J., Blasiak, J., Salminen, A., & Kaarniranta, K. (2016). Inflammation and its role in age-related macular degeneration. *Cellular and molecular life sciences*, *73*(9), 1765-1786. https://doi.org/10.1007/s00018-016-2147-8

L'Homme, L., Sermikli, B. P., Haas, J. T., Fleury, S., Quemener, S., Guinot, V., Barreby, E., Esser, N., Caiazzo, R., Verkindt, H., Legendre, B., Raverdy, V., Cheval, L., Paquot, N., Piette, J., Legrand-Poels, S., Aouadi, M., Pattou, F., Staels, B., & Dombrowicz, D. (2024). Adipose tissue macrophage infiltration and hepatocyte stress increase GDF-15 throughout development of obesity to MASH. *Nat Commun*, *15*(1), 7173. https://doi.org/10.1038/s41467-024-51078-2

Lambert, V., Lecomte, J., Hansen, S., Blacher, S., Gonzalez, M. L., Struman, I., Sounni, N. E., Rozet, E., de Tullio, P., Foidart, J. M., Rakic, J. M., & Noel, A. (2013). Laser-induced choroidal neovascularization model to study age-related macular degeneration in mice. *Nat Protoc*, *8*(11), 2197-2211. https://doi.org/10.1038/nprot.2013.135

Leung, D. W., Cachianes, G., Kuang, W. J., Goeddel, D. V., & Ferrara, N. (1989). Vascular endothelial growth factor is a secreted angiogenic mitogen. *Science*, *246*(4935), 1306-1309. https://doi.org/10.1126/science.2479986

Liu, C. H., Wang, Z., Sun, Y., & Chen, J. (2017). Animal models of ocular angiogenesis: from development to pathologies. *Faseb J*, *31*(11), 4665-4681. https://doi.org/10.1096/fj.201700336R

Menard, C., Wilson, A. M., Dejda, A., Miloudi, K., Binet, F., Crespo-Garcia, S., Parinot, C., Pilon, F., Juneau, R., Andriessen, E. M., Mawambo, G., SanGiovanni, J. P., De Guire, V., & Sapieha, P. (2020). miR-106b suppresses pathological retinal angiogenesis. *Aging (Albany NY)*, *12*(24), 24836-24852. https://doi.org/10.18632/aging.202404

Miller, H., Miller, B., Ishibashi, T., & Ryan, S. J. (1990). Pathogenesis of laser-induced choroidal subretinal neovascularization. *Invest Ophthalmol Vis Sci*, *31*(5), 899-908. https://www.ncbi.nlm.nih.gov/pubmed/1692312

Munzberg, H., Floyd, E., & Chang, J. S. (2021). Sympathetic Innervation of White Adipose Tissue: to Beige or Not to Beige? *Physiology (Bethesda)*, *36*(4), 246-255. https://doi.org/10.1152/physiol.00038.2020

Natarajan, D., Plakkot, B., Tiwari, K., Ekambaram, S., Wang, W., Rudolph, M., Mohammad, M. A., Chacko, S. K., Subramanian, M., Tarantini, S., Yabluchanskiy, A., Ungvari, Z., Csiszar, A., & Balasubramanian, P. (2024). Chronic beta3-AR stimulation activates distinct thermogenic mechanisms in brown and white adipose tissue and improves systemic metabolism in aged mice. *Aging Cell*, *23*(12), e14321. https://doi.org/10.1111/acel.14321

Ouchi, N., Kobayashi, H., Kihara, S., Kumada, M., Sato, K., Inoue, T., Funahashi, T., & Walsh, K. (2004). Adiponectin stimulates angiogenesis by promoting cross-talk between AMP-activated protein kinase and Akt signaling in endothelial cells. *J Biol Chem*, *279*(2), 1304-1309. https://doi.org/10.1074/jbc.M310389200

Patsouris, D., Qi, P., Abdullahi, A., Stanojcic, M., Chen, P., Parousis, A., Amini-Nik, S., & Jeschke, M. G. (2015). Burn Induces Browning of the Subcutaneous White Adipose Tissue in Mice and Humans. *Cell Rep*, *13*(8), 1538-1544. https://doi.org/10.1016/j.celrep.2015.10.028

Sakurai, E., Anand, A., Ambati, B., van Rooijen, N., & Ambati, J. (2003). Macrophage depletion inhibits experimental choroidal neovascularization. *Investigative Ophthalmology & Visual Science*, *44*(8), 3578-3585. HTTP://opurl.bib.umontreal.ca:9003/sfx_local?sid=Entrez%3APubMed&id=pmid%3A12882810

Seale, P., Kajimura, S., Yang, W., Chin, S., Rohas, L. M., Uldry, M., Tavernier, G., Langin, D., & Spiegelman, B. M. (2007). Transcriptional control of brown fat determination by PRDM16. *Cell Metab*, *6*(1), 38-54. https://doi.org/10.1016/j.cmet.2007.06.001

Shah, R. S., Soetikno, B. T., Lajko, M., & Fawzi, A. A. (2015). A Mouse Model for Laser-induced Choroidal Neovascularization. *J Vis Exp*(106), e53502. https://doi.org/10.3791/53502

Sidossis, Labros S., Porter, C., Saraf, Manish K., Børsheim, E., Radhakrishnan, Ravi S., Chao, T., Ali, A., Chondronikola, M., Mlcak, R., Finnerty, Celeste C., Hawkins, Hal K., Toliver-Kinsky, T., & Herndon, David N. (2015). Browning of Subcutaneous White Adipose Tissue in Humans after Severe Adrenergic Stress. *Cell metabolism*, *22*(2), 219-227. https://doi.org/10.1016/j.cmet.2015.06.022

Tan, X., Fujiu, K., Manabe, I., Nishida, J., Yamagishi, R., Terashima, Y., Matsushima, K., Kaburaki, T., Nagai, R., & Yanagi, Y. (2016). Choroidal Neovascularization Is Inhibited in Splenic-Denervated or Splenectomized Mice with a Concomitant Decrease in Intraocular Macrophage. *PLoS ONE*, *11*(8), e0160985. https://doi.org/10.1371/journal.pone.0160985

Wang, G. X., Zhao, X. Y., & Lin, J. D. (2015). The brown fat secretome: metabolic functions beyond thermogenesis. *Trends Endocrinol Metab*, *26*(5), 231-237. https://doi.org/10.1016/j.tem.2015.03.002

Wilson, A. M., Shao, Z., Grenier, V., Mawambo, G., Daudelin, J. F., Dejda, A., Pilon, F., Popovic, N., Boulet, S., Parinot, C., Oubaha, M., Labrecque, N., de Guire, V., Laplante, M., Lettre, G., Sennlaub, F., Joyal, J. S., Meunier, M., & Sapieha, P. (2018). Neuropilin-1 expression in adipose tissue macrophages protects against obesity and metabolic syndrome. *Sci Immunol*, *3*(21). https://doi.org/10.1126/sciimmunol.aan4626

Xu, N., Bo, Q., Shao, R., Liang, J., Zhai, Y., Yang, S., Wang, F., & Sun, X. (2019). Chitinase-3-Like-1 Promotes M2 Macrophage Differentiation and Induces Choroidal Neovascularization in Neovascular Age-Related Macular Degeneration. *Invest Ophthalmol Vis Sci*, *60*(14), 4596-4605. https://doi.org/10.1167/iovs.19-27493

Xu, N., Sun, T., Wang, Y., Tong, X., Lu, S., Yang, F., Wang, J., Bo, Q., Sun, J., & Sun, X. (2023). Dynamic changes in macrophage morphology during the progression of choroidal neovascularization in a laser-induced choroidal neovascularization mouse model. *BMC Ophthalmology*, *23*(1), 401. https://doi.org/10.1186/s12886-023-03141-7

Yang, Y., Liu, F., Tang, M., Yuan, M., Hu, A., Zhan, Z., Li, Z., Li, J., Ding, X., & Lu, L. (2016). Macrophage polarization in experimental and clinical choroidal neovascularization. *Scientific Reports*, *6*(1), 30933. https://doi.org/10.1038/srep30933

Zandi, S., Nakao, S., Chun, K.-H., Fiorina, P., Sun, D., Arita, R., Zhao, M., Kim, E., Schueller, O., Campbell, S., Taher, M., Melhorn, Mark I., Schering, A., Gatti, F., Tezza, S., Xie, F., Vergani, A., Yoshida, S., Ishikawa, K.,…Hafezi-Moghadam, A. (2015). ROCK-Isoform-Specific Polarization of Macrophages Associated with Age-Related Macular Degeneration. *Cell Rep*, *10*(7), 1173-1186. https://doi.org/https://doi.org/10.1016/j.celrep.2015.01.050

Zhang, P., Wang, H., Luo, X., Liu, H., Lu, B., Li, T., Yang, S., Gu, Q., Li, B., Wang, F., & Sun, X. (2018). MicroRNA-155 Inhibits Polarization of Macrophages to M2-Type and Suppresses Choroidal Neovascularization. *Inflammation*, *41*(1), 143-153. https://doi.org/10.1007/s10753-017-0672-8

8th Sep 2025

Dear Prof. Sapieha,

Thank you for submitting your revised manuscript to EMBO Molecular Medicine. We have now received the reports from the three referees. As you will see below, referees #2 and #3 are satisfied with the revisions overall, but referee #1 regrets that no experimental steps were taken to address the mechanism at play.

We have therefore consulted the referees further on this issue. Referee #2 stated: "I would agree with Referee#1 on the importance of this experiment. I raised the same concern in my review. The novelty of this study lies in the discovery of PRDM16-expressing iWAT in promoting CNV. However, the molecular mechanisms are lacking. The authors proposed potential factors in the discussion; they may perform additional experiments to confirm their involvement. In addition, although laser-induced CNV is a commonly used model for neoangiogenesis, it does not involve other factors in AMD pathogenesis. Without confirming the findings in other AMD models or demonstrating the involvement of this pathway in human AMD samples, it is not appropriate to conclude that this pathway contributes to AMD pathogenesis. (running title and abstract)"

After discussion within the team, we would thus like to invite you to revise the manuscript further to provide some level of mechanistic insight. While we understand that the identification and validation of the exact molecule(s) involved may be beyond the scope of this study, we encourage you to conduct experiments that could shed further light on the mechanism.

If adding other AMD models (animal or human) is not possible, please tone down the corresponding text in the manuscript.

If you would like to discuss further these or other points, I am available to do so via email or video. Let me know if you are interested in this option.

As EMBO Press usually only allows one round of revisions, please be aware that this will be your last opportunity to address these issues. The revised manuscript will be reviewed again, and we cannot guarantee a positive outcome at this stage.

Moreover, please address the following editorial requests:

1/ Please provide the figures in individual high-resolution files.
2/ Acknowledgements: the information provided in the manuscript text and in the submission system should match. Please check whether The Courtois Foundation should be added to the list of funders in our system.
3/ Please remove the Authors Contributions from the manuscript and use the free text boxes beneath each contributing author's name in our system to add specific details on the author's contribution.
4/ Please provide The Paper Explained: EMBO Molecular Medicine articles are accompanied by a summary of the articles to emphasize the major findings in the paper and their medical implications for the non-specialist reader. Please provide a draft summary of your article highlighting
- the medical issue you are addressing,
- the results obtained and
- their clinical impact.
This may be edited to ensure that readers understand the significance and context of the research. Please refer to any of our published articles for an example.
5/ The references need reformatting to alphabetical order, and ten author names should be listed before et al.
6/ The two supplementary tables should be renamed Table EV1 and EV2, and both should get a short legend/description added to the top of each page.
7/ Source Data: the western blot data should be added to the ZIP folder with the remaining numerical data for the corresponding figure. We do not allow powerpoint format, please use PDF, jpg of TIF for the blots.
8/ Please download and fill our Reagents and Tools Table template (.docx), which you can find in our author guidelines: https://www.embopress.org/page/journal/14693178/authorguide#structuredmethods. When submitting your revised manuscript, please do not include the Reagents and Tools Table in the Methods section of the manuscript but upload it as a separate file choosing the file type "Reagent Table".
9/ Please upload the synopsis image as an individual jpeg/tiff/png file without the synopsis text. Please upload the synopsis text as an individual word file.
10/ Figure callouts: There is a callout for a Supplemental Table 1, please correct; a callout is missing for the second table (as Table EV2).
11/ Please address the queries from the data editors in the figure legends:
- Please define the annotated p values ****/***/**/* as well as provide the exact p-values for the same in the legend of figure EV1 A, EV4 A, B as appropriate.
- Please note that the exact p values are not provided in the legends of figures 1D F, G; 2A, F; 3B, E, G, H, I; 4E, F, H, I; EV3 B, E, F; EV5 F, G, H, I, J, K, L, M; EV5 F, G, H, I, J, K, L, M ; EV6 B, C.

- Please indicate the statistical test used for data analysis in the legends of figures 1D, F, G; 2A, 3B, E G, H, I; 4E, H, I; EV1 A, E; EV3 B, E, F; EV4 A, B; EV5 F, G, H, I, J, K, L, M ; EV6 B, C.
- Please note that information related to n is missing in the legend of figure EV5 C
- Please note that the error bars are not defined in the legends of figures 2A, B, C, D, F, G; EV1 A, B, C, E, G, H; EV5 C

As part of the EMBO Publications transparent editorial process initiative (see our Editorial at http://embomolmed.embopress.org/content/2/9/329), EMBO Molecular Medicine will publish online a Review Process File (RPF) to accompany accepted manuscripts.
This file will be published in conjunction with your paper and will include the anonymous referee reports, your point-by-point response and all pertinent correspondence relating to the manuscript. Let us know whether you agree with the publication of the RPF and as here, if you want to remove or not any figures from it prior to publication.
Please note that the Authors checklist will be published at the end of the RPF.

I look forward to receiving your revised manuscript.

With kind regards,

Lise

Lise Roth, PhD
Senior Editor
EMBO Molecular Medicine

***** Reviewer's comments *****

Referee #1 (Remarks for Author):

Unfortunately, the revised manuscript does not include any new experimental data, although some additional discussion has been incorporated in response to the comments from the three reviewers. In particular, it is regrettable that the only major point I raised has been positioned merely as a "next logical step." Therefore, my overall assessment remains unchanged: the key mechanism is still not adequately addressed.

In addition, the explanation regarding the FITC+IB4- substances is not accurate. IB4 labels all blood vessels; if it does not, this suggests the presence of a technical issue in the authors' experiments.

Referee #2 (Remarks for Author):

The authors have addressed all my questions. Although the authors provide good rationale for using the laser-CNV model, it would be nice to see that the results can be reproduced in other models of neovascular AMD and CNV. It is also important to investigate the potential implication of this pathway in human AMD and other neovascular retinal disease. I look forward to their future studies on this topic.

Referee #3 (Remarks for Author):

I would like to thank the authors for their detailed response that addressed all my questions/concerns.

Response to editors and reviewers: Diaz-Marin *et al* Adipocytes influences choroidal neovascularization via PRDM16.

Question that remained from reviewer 1: One major question still remains; What molecule(s) link adipocytes and ocular neovessels after all? Is it an adipocyte-derived metabolite or an adipokine? In this reviewer's opinion, the authors should attempt to identify it using the co-culture system in Figure 4G.

R1, answer 1: We would like to thank the editors and reviewers for their request to investigate potential angiogenic effectors from PRDM16-expressing adipocytes that could contribute to choroidal neovascularization. We have now performed the experiments on MS-MS on adipocyte secretomes that you and your reviewers requested. Our data points to a series of angiogenic and AMD-relevant proteins produced by PRDM16. We have now incorporated this data into **Figure 4** of the manuscript.

Specifically, a total of 340 proteins were differentially regulated in the PRDM16$^{+/+}$ adipocyte culture media compared to that of PRDM16$^{-/-}$ adipocytes, the majority of which were significantly reduced (**Note to reviewers Figure 1**). All top 10 upregulated and top 10 downregulated proteins were validated in ExoCarta, a compendium of exosomal cargo (Keerthikumar et al., 2016), with the exception of Niban1 which was validated in Vesiclepedia, an extracellular vesicle and extracellular particles repository (Chitti et al., 2024).

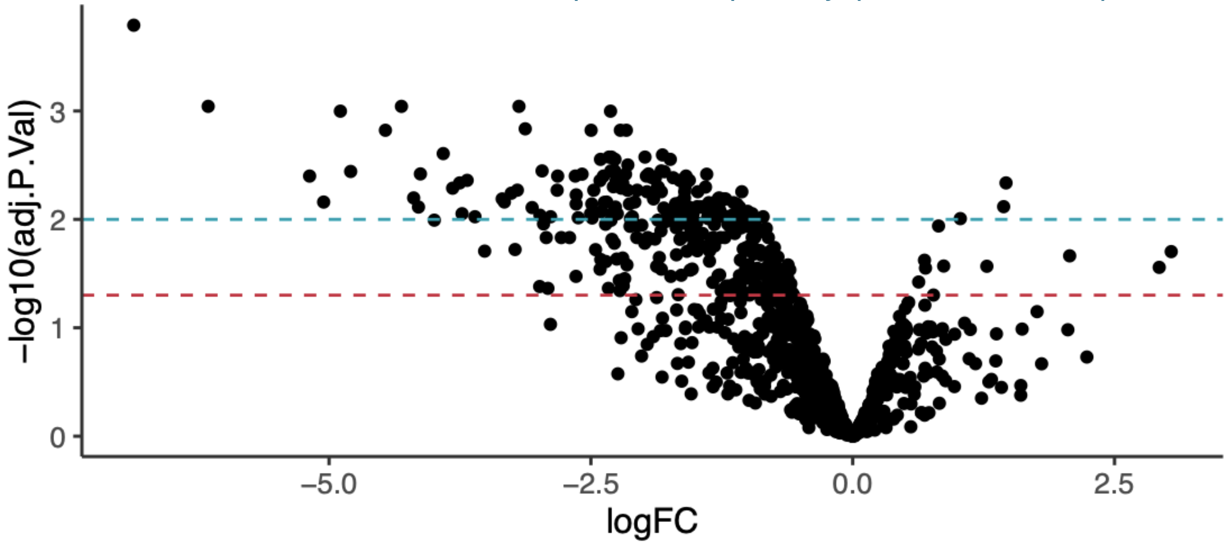

Note to reviewers Figure 1: Volcano plot displaying the protein expression fold change and P-value (-log10 transformation) for PRDM16$^{+/+}$ adipocyte secretome versus PRDM16$^{-/-}$ adipocyte secretome. The red line represents p=0.05, and the blue line p=0.01.

Description of Secreted Protein Enriched in PRDM16$^{+/+}$ vs PRDM16$^{-/-}$ adipocytes

Of the most regulated proteins by PRDM16 (70%) have been implicated in retinal neovascularization, including neovascular AMD (**Note to reviewers Table 1**).

Identifier	logFC	AveExpr	t	P.Value	adj.P.Val	B
Igfbp5	1.4639683	0.7192522	7.579991	0.0002266	0.0046101	1.0620316
Mmp14	1.4411157	0.6947084	6.177267	0.0007115	0.0076312	-0.1808943
Ogn	1.0274961	0.5062909	5.417723	0.0014403	0.0098354	-0.9519005
Adgre1	0.8196358	0.4084402	5.124444	0.0019269	0.0115038	-1.2705135
Niban1	3.0412803	1.1467879	4.326014	0.0045150	0.0198303	-2.2014991
Adam10	2.0714547	0.7864075	4.227295	0.0050483	0.0216946	-2.3232589
Olfml2b	0.6853603	0.3425902	4.116171	0.0057343	0.0238213	-2.4620849
Adam9	0.8687415	0.4117955	3.989612	0.0066449	0.0269309	-2.6224609
Gpx3	1.2809003	0.6192760	3.978303	0.0067338	0.0270711	-2.6369078
Itgb2	2.9268392	0.9735907	3.951650	0.0069486	0.0277110	-2.6710326

Note to reviewers Table 1: Top 10 enriched proteins in PRDM16$^{+/+}$ versus PRDM16$^{-/-}$ adipocyte secretomes (as per Reactome Camera Analysis relative to PRDM16$^{-/-}$).

Insulin-like growth factor-binding protein 5 (IGFBP-5) regulates insulin-like growth factors (IGFs). IGFs have been implicated in pathological neovascularization in multiple diseases including diabetic retinopathy, wet AMD and retinopathy of prematurity. Patients with neovascular AMD who received intravitreal injections of anti-VEGF ranibizumab had their circulating levels of IGF family members profiled before and after treatment, and IGF and IGFBPS members including IGFBP-5 were shown to be significantly downregulated in patient PBMCs following ranibizumab treatment (Strzalka-Mrozik et al., 2017). Furthermore, human retinal endothelial cell cultures of diabetic origin had higher levels of IGFBP-5 mRNA and protein levels compared to non-diabetic controls (Spoerri et al., 1998). IGFBP5 levels were also increased in neovascular tufts in the oxygen-induced retinopathy (OIR) mouse model (Lofqvist et al., 2009).

Matrix Metalloproteinase 14 (MMP14), an enzyme that drives extracellular matrix (ECM) remodelling, is a biomarker of angiogenic activity in proliferative diabetic retinopathy (PDR). MMP14 levels were significantly higher in the vitreous samples from patients with PDR compared to nondiabetic controls, with the highest levels found in patients with active neovascularization compared to inactive PDR (Abu El-Asrar et al., 2018).

Osteoglycin (OGN) or mimecan, a small leucine-rich proteoglycan important to ECM organization, has been shown to be significantly upregulated in rat retinas in an streptozotocin-induced diabetic retinopathy model (Na et al., 2023).

A Disintegrin and Metalloproteinase 10 (ADAM10) enables Notch signaling. ADAM10 was significantly upregulated in ischemic retinas of the OIR mouse model, and its downregulation inhibited the proliferation, sprouting, migration and tube formation of human microvascular endothelial cells (Bisen et al., 2025). An additional upregulated ADAM family member, ADAM9, was also shown to be involved in pathological retinal neovascularization (Guaiquil et al., 2009). ADAM9 expression is increased in endothelial cells in pathological vascular tufts in the OIR model, and pathological neovascularization was reduced in the OIR and CNV models in Adam9$^{-/-}$ mice compared to wild-type controls.

Glutathione peroxidase-3 (GPX3), an antioxidant enzyme, was identified as a novel biomarker in neovascular AMD, as its expression increased in the aqueous humor of patients with anti-VEGF-resistant nAMD who demonstrated choroidal neovascularization activity during follow-up (Coronado et al., 2021).

Integrin Subunit Beta 2 (ITGB2, or CD18) is an inflammatory and immune-related gene whose targeted disruption in transgenic mice resulted in reduced choroidal neovascularization following laser-burn (Sakurai et al., 2003).

Collectively, increased levels of IGFBP-5, MMP14, OGN, ADAM10, ADAM9, GPX3 and ITGB2 were associated to increased pathological neovascularization, and their targeted disruption in some of these aforementioned studies rescued this phenotype. The loss of these proteins in the PRDM16$^{-/-}$ secretome compared to control PRDM16$^{+/+}$ could provide a mechanistic link to the significantly higher levels of choroidal neovascularization and sprouting observed in Adipo-Cre-PRDM16$^{+/+}$ mice compared to Adipo-Cre-PRDM16$^{-/-}$ mice.
The remaining three proteins in the top 10 proteins that are reduced in PRDM16$^{-/-}$ adipocytes are ADGRE1, NIBAN1 and OLFML2B. Adhesion G protein-coupled receptor E1 (ADGRE1), also known as F4/80 in mice, is a resident microglia marker of the retina. Microglia, when adequately polarized can contribute to neovascularization. Niban Apoptosis Regulator 1 (NIBAN1) is involved in the endoplasmic reticulum (ER) stress response. Olfactomedin Like 2B (OLFML2B) is a secreted glycoprotein overexpressed in various cancers (Hu et al., 2022). Neither NIBAN1 nor OLFML2B have been implicated in pathological neovascularization.

Identifier	logFC	AveExpr	t	P.Value	adj.P.Val	B
Idh3a	-6.864915	-3.483098	-25.19462	2.00e-07	0.0001618	7.897952
Ppp1r7	-3.186751	-1.611273	-15.44389	3.30e-06	0.0009068	5.385823
Aco2	-6.154128	-3.122420	-15.21272	3.60e-06	0.0009068	5.299474
Letm1	-4.308805	-2.170667	-15.19598	3.60e-06	0.0009068	5.293153
Bphl	-4.891873	-2.466830	-14.11763	5.70e-06	0.0010041	4.866000
Ndufab1	-2.312339	-1.161921	-13.98036	6.00e-06	0.0010041	4.808645
Ech1	-3.126846	-1.575664	-12.81147	1.03e-05	0.0014606	4.290218
Atp5f1b	-2.497989	-1.253429	-12.39498	1.25e-05	0.0015062	4.091497
Lactb2	-4.461894	-2.235323	-12.20665	1.37e-05	0.0015062	3.999029
Rhoa	-2.161395	-1.085067	-11.85575	1.64e-05	0.0015062	3.822222

Note to reviewers Table 2: Top 10 enriched proteins in PRDM16$^{-/-}$ versus PRDM16$^{+/+}$ adipocyte secretomes (as per Reactome Camera Analysis relative to PRDM16$^{-/-}$).
Seven of the top ten proteins with lower expression in PRDM16$^{+/+}$ adipocyte secretome compared to PRDM16$^{-/-}$ were mitochondrial, with roles implicated in the tricarboxylic acid cycle (IDH3A), citric acid cycle (ACO2), ion homeostasis (LETM1), fatty acid beta-oxidation (NDUFAB1, ECH1), oxidative phosphorylation (ATP5F1b), and turnover and processing of mitochondrial RNA (LACTB2) (**Table 2**). These proteins likely originate from small extracellular vesicles released in the culture media, as all were validated exosomal cargo proteins. As these signaling molecules reflect the metabolic state of their parental cell (Rohm et al., 2025), these results suggest that mitochondrial metabolism is differentially regulated in PRDM16$^{+/+}$ adipocytes compared to PRDM16$^{-/-}$ adipocytes.
Of the remaining top 10 lower expressed proteins in PRDM16$^{+/+}$ adipocyte secretomes, Protein Phosphatase 1 Regulatory Subunit 7 (PPP1R7), negatively regulates the AKT kinase signaling pathway and abrogates cell migration (Paul et al., 2019), and Ras Homolog family member A (RHOA) inhibits endothelial cell proliferation, migration, tube formation and angiogenic sprouting in vitro (Hauke et al., 2022). Although investigations on RhoA signaling with regards to microvesicles have been focused on its role in microvesicle biogenesis (Li et al., 2012; Sedgwick et al., 2015), its presence in exosomal cargo has been detected in multiple species collated in the ExoCarta database. The reduced expression of these two proteins in the PRDM16$^{+/+}$ secretome could help explain the significantly higher levels of choroidal neovascularization and choroidal sprouting detected in Adipo-Cre-PRDM16$^{+/+}$ mice compared to Adipo-Cre-PRDM16$^{-/-}$ mice. Biphenyl Hydrolase Like protein (BPHL), a serine

hydrolase that plays a role in drug metabolism and detoxification, has no known role in angiogenesis or inflammation.

CONCLUSIONS:

Collectively, these data suggest that PRDM16 expression in adipocytes is associated with expression of proteins with roles in neovascular AMD and further solidify the link between PRDM16 and disease progression.

We believe this data sufficiently satisfies your requests and that of your reviewers. We have attached an outline of the data and experimental procedures. If you agree, we can incorporate the new data in the manuscript.

REFERENCES:

Abu El-Asrar, A. M., Mohammad, G., Allegaert, E., Ahmad, A., Siddiquei, M. M., Alam, K., Gikandi, P. W., De Hertogh, G., & Opdenakker, G. (2018). Matrix metalloproteinase-14 is a biomarker of angiogenic activity in proliferative diabetic retinopathy. *Mol Vis*, *24*, 394-406.

Bisen, S., Gogoi, P., Sharma, A., Mukhopadhyay, C. S., & Singh, N. K. (2025). A Disintegrin and Metalloproteinase 10 Regulates Ephrin B2-Mediated Endothelial Cell Sprouting and Ischemic Retinopathy. *Am J Pathol*, *195*(7), 1311-1327. https://doi.org/10.1016/j.ajpath.2025.03.007

Chitti, S. V., Gummadi, S., Kang, T., Shahi, S., Marzan, A. L., Nedeva, C., Sanwlani, R., Bramich, K., Stewart, S., Petrovska, M., Sen, B., Ozkan, A., Akinfenwa, M., Fonseka, P., & Mathivanan, S. (2024). Vesiclepedia 2024: an extracellular vesicles and extracellular particles repository. *Nucleic Acids Res*, *52*(D1), D1694-d1698. https://doi.org/10.1093/nar/gkad1007

Coronado, B. N. L., da Cunha, F. B. S., de Oliveira, R. M., Nóbrega, O. T., Ricart, C. A. O., Fontes, W., de Sousa, M. V., de Ávila, M. P., & Martins, A. M. A. (2021). Novel Possible Protein Targets in Neovascular Age-Related Macular Degeneration: A Pilot Study Experiment. *Front Med (Lausanne)*, *8*, 692272. https://doi.org/10.3389/fmed.2021.692272

Guaiquil, V., Swendeman, S., Yoshida, T., Chavala, S., Campochiaro, P. A., & Blobel, C. P. (2009). ADAM9 is involved in pathological retinal neovascularization. *Mol Cell Biol*, *29*(10), 2694-2703. https://doi.org/10.1128/mcb.01460-08

Hauke, M., Eckenstaler, R., Ripperger, A., Ender, A., Braun, H., & Benndorf, R. A. (2022). Active RhoA Exerts an Inhibitory Effect on the Homeostasis and Angiogenic Capacity of Human Endothelial Cells. *Journal of the American Heart Association*, *11*(12), e025119. https://doi.org/10.1161/JAHA.121.025119

Hu, P., Zhang, X., Li, Y., Xu, L., & Qiu, H. (2022). Pan-Cancer Analysis of OLFML2B Expression and Its Association With Prognosis and Immune Infiltration. *Front Genet*, *13*, 882794. https://doi.org/10.3389/fgene.2022.882794

Keerthikumar, S., Chisanga, D., Ariyaratne, D., Al Saffar, H., Anand, S., Zhao, K., Samuel, M., Pathan, M., Jois, M., Chilamkurti, N., Gangoda, L., & Mathivanan, S. (2016). ExoCarta: A Web-Based Compendium of Exosomal Cargo. *J Mol Biol*, *428*(4), 688-692. https://doi.org/10.1016/j.jmb.2015.09.019

Li, B., Antonyak, M. A., Zhang, J., & Cerione, R. A. (2012). RhoA triggers a specific signaling pathway that generates transforming microvesicles in cancer cells. *Oncogene*, *31*(45), 4740-4749. https://doi.org/10.1038/onc.2011.636

Lofqvist, C., Willett, K. L., Aspegren, O., Smith, A. C. H., Aderman, C. M., Connor, K. M., Chen, J., Hellstrom, A., & Smith, L. E. H. (2009). Quantification and Localization of the IGF/Insulin System Expression in Retinal Blood Vessels and Neurons during Oxygen-Induced Retinopathy

in Mice. *Investigative Ophthalmology & Visual Science*, *50*(4), 1831-1837. https://doi.org/10.1167/iovs.08-2903

Na, L., Xu, M., Chen, J.-L., Chen, G.-J., Sun, J., Zhang, Q., Li, J.-Q., Guo, X.-L., Zuo, Z.-F., Liu, X.-Z., & Wang, T.-H. (2023). 4D-DIA quantitative proteomics revealed the core mechanism of diabetic retinopathy after berberine treatment. *European Journal of Pharmacology*, *958*, 175947. https://doi.org/https://doi.org/10.1016/j.ejphar.2023.175947

Paul, D., Bargale, A. B., Rapole, S., Shetty, P. K., & Santra, M. K. (2019). Protein Phosphatase 1 Regulatory Subunit SDS22 Inhibits Breast Cancer Cell Tumorigenesis by Functioning as a Negative Regulator of the AKT Signaling Pathway. *Neoplasia*, *21*(1), 30-40. https://doi.org/https://doi.org/10.1016/j.neo.2018.10.009

Rohm, T. V., Cunha, E. R. K., & Olefsky, J. M. (2025). Metabolic Messengers: small extracellular vesicles. *Nat Metab*, *7*(2), 253-262. https://doi.org/10.1038/s42255-024-01214-5

Sakurai, E., Taguchi, H., Anand, A., Ambati, B. K., Gragoudas, E. S., Miller, J. W., Adamis, A. P., & Ambati, J. (2003). Targeted disruption of the CD18 or ICAM-1 gene inhibits choroidal neovascularization. *Invest Ophthalmol Vis Sci*, *44*(6), 2743-2749. https://doi.org/10.1167/iovs.02-1246

Sedgwick, A. E., Clancy, J. W., Olivia Balmert, M., & D'Souza-Schorey, C. (2015). Extracellular microvesicles and invadopodia mediate non-overlapping modes of tumor cell invasion. *Scientific Reports*, *5*(1), 14748. https://doi.org/10.1038/srep14748

Spoerri, P. E., Ellis, E. A., Tarnuzzer, R. W., & Grant, M. B. (1998). Insulin-like growth factor: receptor and binding proteins in human retinal endothelial cell cultures of diabetic and non-diabetic origin. *Growth Hormone & IGF Research*, *8*(2), 125-132. https://doi.org/https://doi.org/10.1016/S1096-6374(98)80102-0

Strzalka-Mrozik, B., Kimsa-Furdzik, M., Kabiesz, A., Michalska-Malecka, K., Nita, M., & Mazurek, U. (2017). Gene expression levels of the insulin-like growth factor family in patients with AMD before and after ranibizumab intravitreal injections. *Clin Interv Aging*, *12*, 1401-1408. https://doi.org/10.2147/cia.S135030

30th Mar 2026

Dear Prof. Sapieha, Dear Mike,

Thank you for submitting your revised study. We have now received the referee's report, and as you will see below, they are satisfied with the revisions. I will therefore be able to accept your manuscript once the following editorial matters are addressed:

1/ Manuscript text:
- Please indicate any new modification in track changes mode.
- Methods:
o Studies involving experimental animals: if applicable, provide reference number for approval. Include a statement of compliance with ethical regulations.
o Statistics: please include a statement about blinding even if no blinding was done.
- Data availability section: please deposit the proteomics data in an appropriate database.
- Acknowledgements: Please check whether The Courtois Foundation should be added to the list of funders in our submission system.

2/ Figures:
- Please address the queries from our data editors in the figure legends:
1. Please provide the exact p values (in the figures or their legends) for figures 1D F, G; 2A, F; 3B, E, G, H, I; 4E, F, H, I; EV3 B, E, F; EV5 F, G, H, I, J, K, L, M; EV5 F, G, H, I, J, K, L, M ; EV6 B, C.
2. Please indicate the statistical test used for data analysis in the legends of figures 1D, F, G; 2A, 3B, E G, H, I; 4E, H, I; EV1 A, E; EV3 B, E, F; EV4 A, B; EV5 F, G, H, I, J, K, L, M ; EV6 B, C.
3. Please note that information related to n is missing in the legend of figure EV5 C
4. Please note that the error bars are not defined in the legends of figures 2A, B, C, D, F, G; EV1 A, B, C, E, G, H; EV5 C

3/ Author checklist:
Please fill in the data availability section

4/ Source Data:
Thank you for providing the numerical Source Data. Please carefully check that the values presented in summary excel files are correct, as some duplications were found (see for instance file attached, colored boxes).
The source data for figure 4E and 4G are missing.
Please correct labeling from S1A to EV1A, etc.

5/ Thank you for providing a synopsis text. Please remove it from the manuscript text and reformat it as follows:
Synopses include a short stand first (maximum of 300 characters, including space) as well as 2-5 one-sentences bullet points that summarizes the paper. Please write the bullet points to summarize the key NEW findings. They should be designed to be complementary to the abstract - i.e. not repeat the same text. We encourage inclusion of key acronyms and quantitative information (maximum of 30 words / bullet point).

6/ The graphical abstract should be uploaded as an individual jpeg/tiff/png file 550 px wide x 300 px high.

7/ As part of the EMBO Publications transparent editorial process initiative (see our Editorial at http://embomolmed.embopress.org/content/2/9/329), EMBO Molecular Medicine will publish online a Review Process File (RPF) to accompany accepted manuscripts.
This file will be published in conjunction with your paper and will include the anonymous referee reports, your point-by-point response and all pertinent correspondence relating to the manuscript. Let us know whether you agree with the publication of the RPF and as here, if you want to remove or not any figures from it prior to publication.
Please note that the Authors checklist will be published at the end of the RPF.

I look forward to receiving your revised manuscript.

With kind regards,

Lise

Lise Roth, PhD
Senior Editor
EMBO Molecular Medicine

***** Reviewer's comments *****

Referee #1 (Remarks for Author):

I have carefully reviewed the point-by-point responses in the revised manuscript. The authors have adequately addressed my previous concerns and strengthened the data. The manuscript is now acceptable for publication.

The authors addressed the remaining editorial issues.

23rd Apr 2026

Dear Prof. Sapieha, Dear Mike,

Thank you for submitting your revised files. I am pleased to inform you that your manuscript is now accepted for publication in EMBO Molecular Medicine!

Before we can send the manuscript to production to be included in the next available issue of EMBO Molecular Medicine, the following should be addressed:
- The proteomics datasets are not publicly available.
- The visual abstract is not in the correct size. Unfortunately, when resized to 550x300 px, the text is not legible anymore. Could you please resize and adapt the font size? Please also remove the title 'graphical abstract'.
- Please let us know if you agree with the publication of the Review Process File in conjunction with the manuscript.

Your manuscript will be processed for publication by EMBO Press. It will be copy edited and you will receive page proofs prior to publication. Please note that you will be contacted by Springer Nature Author Services to complete licensing and payment information.

You may qualify for financial assistance for your publication charges - either via a Springer Nature fully open access agreement or an EMBO initiative. Check your eligibility: https://link.springer.com/journal/44321/how-to-publish-with-us

Should you be planning a Press Release on your article, please get in contact with embo_production@springernature.com as early as possible in order to coordinate publication and release dates.

If you have any questions, please do not hesitate to contact the Editorial Office.

Congratulations on your nice study!

With kind regards,

Lise

Lise Roth, Ph.D
Senior Editor
EMBO Molecular Medicine

>>> Please note that it is EMBO Molecular Medicine policy for the transcript of the editorial process (containing referee reports and your response letter) to be published as an online supplement to each paper. If you do NOT want this, you will need to inform the Editorial Office via email immediately. More information is available here: https://link.springer.com/partners/embo-press/editorial-policies#Peer%20review

EMBO Press Author Checklist

Corresponding Author Name: Przemyslaw SAPIEHA, Ariel WILSON, Sergio CRESPO-GARCIA
Journal Submitted to: EMBO Molecular Medicine
Manuscript Number: EMM-2025-21611

USEFUL LINKS FOR COMPLETING THIS FORM
The EMBO Journal - Author Guidelines
EMBO Reports - Author Guidelines
Molecular Systems Biology - Author Guidelines
EMBO Molecular Medicine - Author Guidelines

Reporting Checklist for Life Science Articles (updated January
This checklist is adapted from Materials Design Analysis Reporting (MDAR) Checklist for Authors. MDAR establishes a minimum set of requirements in transparent reporting in the life sciences (see Statement of Task: 10.31222/osf.io/9sm4x). Please follow the journal's guidelines in preparing your
Please note that a copy of this checklist will be published alongside your article.

Abridged guidelines for figures
1. Data
The data shown in figures should satisfy the following conditions:
- the data were obtained and processed according to the field's best practice and are presented to reflect the results of the experiments in an accurate and unbiased manner.
- ideally, figure panels should include only measurements that are directly comparable to each other and obtained with the same assay.
- plots include clearly labeled error bars for independent experiments and sample sizes. Unless justified, error bars should not be shown for technical
- if n<5, the individual data points from each experiment should be plotted. Any statistical test employed should be justified.
- Source Data should be included to report the data underlying figures according to the guidelines set out in the authorship guidelines on Data

2. Captions
Each figure caption should contain the following information, for each panel where they are relevant:
- a specification of the experimental system investigated (eg cell line, species name).
- the assay(s) and method(s) used to carry out the reported observations and measurements.
- an explicit mention of the biological and chemical entity(ies) that are being measured.
- an explicit mention of the biological and chemical entity(ies) that are altered/varied/perturbed in a controlled manner.
- the exact sample size (n) for each experimental group/condition, given as a number, not a range;
- a description of the sample collection allowing the reader to understand whether the samples represent technical or biological replicates (including how many animals, litters, cultures, etc.).
- a statement of how many times the experiment shown was independently replicated in the laboratory.
- definitions of statistical methods and measures:
  - common tests, such as t-test (please specify whether paired vs. unpaired), simple χ2 tests, Wilcoxon and Mann-Whitney tests, can be unambiguously identified by name only, but more complex techniques should be described in the methods section;
  - are tests one-sided or two-sided?
  - are there adjustments for multiple comparisons?
  - exact statistical test results, e.g., P values = x but not P values < x;
  - definition of 'center values' as median or average;
  - definition of error bars as s.d. or s.e.m.

Please complete ALL of the questions below.
Select "Not Applicable" only when the requested information is not relevant for your study.

Materials

Newly Created Materials	Information included in the manuscript?	In which section is the information available? (Reagents and Tools Table, Materials and Methods, Figures, Data Availability Section)
New materials and reagents need to be available; do any restrictions apply?	Not Applicable	

Antibodies	Information included in the manuscript?	In which section is the information available? (Reagents and Tools Table, Materials and Methods, Figures, Data Availability Section)
For **antibodies** provide the following information:<br>- Commercial antibodies: RRID (if possible) or supplier name, catalogue number and or/clone number<br>- Non-commercial: RRID or citation	Yes	Methods, Section: Histology Analysis; Supplemental Table 2

DNA and RNA sequences	Information included in the manuscript?	In which section is the information available? (Reagents and Tools Table, Materials and Methods, Figures, Data Availability Section)
**Short novel DNA or RNA including primers, probes**: provide the sequences.	Yes	Supplemental Table 1

Cell materials	Information included in the manuscript?	In which section is the information available? (Reagents and Tools Table, Materials and Methods, Figures, Data Availability Section)
**Cell lines:** Provide species information, strain. Provide accession number in repository **OR** supplier name, catalog number, clone number, and/**OR** RRID.	Not Applicable	
**Primary cultures:** Provide species, strain, sex of origin, genetic modification status.	Yes	Methods, section: Isolation of iWAT primary inguinal preadipocytes
Report if the cell lines were recently **authenticated** (e.g., by STR profiling) and tested for mycoplasma contamination.	Not Applicable	

Experimental animals	Information included in the manuscript?	In which section is the information available? (Reagents and Tools Table, Materials and Methods, Figures, Data Availability Section)
**Laboratory animals or Model organisms:** Provide species, strain, sex, age, genetic modification status. Provide accession number in repository **OR** supplier name, catalog number, clone number, **OR** RRID.	Yes	Methods, Section: Animal models
**Animal observed in or captured from the field:** Provide species, sex, and age where possible.	Not Applicable	
Please detail **housing and husbandry conditions**.	Yes	Methods, Section: Animal models

Plants and microbes	Information included in the manuscript?	In which section is the information available? (Reagents and Tools Table, Materials and Methods, Figures, Data Availability Section)
**Plants:** provide species and strain, ecotype and cultivar where relevant, unique accession number if available, and source (including location for collected wild specimens).	Not Applicable	
**Microbes:** provide species and strain, unique accession number if available, and source.	Not Applicable	

Human research participants	Information included in the manuscript?	In which section is the information available? (Reagents and Tools Table, Materials and Methods, Figures, Data Availability Section)
If collected and within the bounds of privacy constraints report on age, sex and gender or ethnicity for all study participants.	Not Applicable	

Core facilities	Information included in the manuscript?	In which section is the information available? (Reagents and Tools Table, Materials and Methods, Figures, Data Availability Section)
If your work benefited from core facilities, was their service mentioned in the acknowledgments section?	Yes	Acknowledgements

Design

Study protocol	Information included in the manuscript?	In which section is the information available? (Reagents and Tools Table, Materials and Methods, Figures, Data Availability Section)
If study protocol has been **pre-registered, provide DOI in the manuscript**. For clinical trials, provide the trial registration number **OR** cite DOI.	Not Applicable	
Report the **clinical trial registration number** (at ClinicalTrials.gov or equivalent), where applicable.	Not Applicable	

Laboratory protocol	Information included in the manuscript?	In which section is the information available? (Reagents and Tools Table, Materials and Methods, Figures, Data Availability Section)
Provide DOI OR other citation details if **external detailed step-by-step protocols** are available.	Not Applicable	

Experimental study design and statistics	Information included in the manuscript?	In which section is the information available? (Reagents and Tools Table, Materials and Methods, Figures, Data Availability Section)
Include a statement about **sample size** estimate even if no statistical methods were used.	Yes	Statistical analyses
Were any steps taken to minimize the effects of subjective bias when allocating animals/samples to treatment (e.g. **randomization procedure**)? If yes, have they been described?	Yes	Methods: Animal models.
Include a statement about **blinding** even if no blinding was done.	Yes	Statistical analyses
Describe **inclusion/exclusion criteria** if samples or animals were excluded from the analysis. Were the criteria pre-established?<br>If sample or data points were omitted from analysis, report if this was due to attrition or intentional exclusion and provide justification.	Yes	Methods: Animal models, Adipose Tissue Transplantation (ATT) model
For every figure, are **statistical tests** justified as appropriate? Do the data meet the assumptions of the tests (e.g., normal distribution)? Describe any methods used to assess it. Is there an estimate of variation within each group of data? Is the variance similar between the groups that are being statistically compared?	Yes	Statistical analyses

Sample definition and in-laboratory replication	Information included in the manuscript?	In which section is the information available? (Reagents and Tools Table, Materials and Methods, Figures, Data Availability Section)
In the figure legends: state number of times the experiment was **replicated** in laboratory.	Yes	Statistical analyses
In the figure legends: define whether data describe **technical or biological replicates**.	Yes	Statistical analyses, Figure legends

Ethics

Ethics	Information included in the manuscript?	In which section is the information available? (Reagents and Tools Table, Materials and Methods, Figures, Data Availability Section)
Studies involving **human participants**: State details of **authority granting ethics approval** (IRB or equivalent committee(s), provide reference number for approval.	Not Applicable	
Studies involving **human participants**: Include a statement confirming that **informed consent** was obtained from all subjects and that the experiments conformed to the principles set out in the WMA Declaration of Helsinki and the Department of Health and Human Services Belmont Report.	Not Applicable	
Studies involving **human participants: For** publication of **patient photos**, include a statement confirming that consent to publish was obtained.	Not Applicable	
Studies involving experimental **animals**: State details of **authority granting ethics approval** (IRB or equivalent committee(s), provide reference number for approval. Include a statement of compliance with ethical regulations.	Yes	Methods, Animal models
Studies involving **specimen and field samples:** State if relevant **permits** obtained, provide details of authority approving study; if none were required, explain why.	Not Applicable	

Dual Use Research of Concern (DURC)	Information included in the manuscript?	In which section is the information available? (Reagents and Tools Table, Materials and Methods, Figures, Data Availability Section)
Could your study fall under dual use research restrictions? Please check biosecurity documents and list of **select agents and toxins** (CDC): https://www.selectagents.gov/sat/list.htm	Not Applicable	
If you used a select agent, is the security level of the lab appropriate and reported in the manuscript?	Not Applicable	
If a study is subject to dual use research of concern regulations, is the name of the **authority granting approval and reference number** for the regulatory approval provided in the manuscript?	Not Applicable	

Reporting

The MDAR framework recommends adoption of discipline-specific guidelines, established and endorsed through community initiatives. Journals have their own policy about requiring specific guidelines and recommendations to complement MDAR.

Adherence to community standards	Information included in the manuscript?	In which section is the information available? (Reagents and Tools Table, Materials and Methods, Figures, Data Availability Section)
State if relevant guidelines or checklists (e.g., **ICMJE, MIBBI, ARRIVE, PRISMA**) have been followed or provided.	Not Applicable	
For **tumor marker prognostic studies,** we recommend that you follow the **REMARK** reporting guidelines (see link list at top right). See author guidelines, under 'Reporting Guidelines'. Please confirm you have followed these guidelines.	Not Applicable	
For **phase II and III randomized controlled trials**, please refer to the **CONSORT** flow diagram (see link list at top right) and submit the CONSORT checklist (see link list at top right) with your submission. See author guidelines, under 'Reporting Guidelines'. Please confirm you have submitted this list.	Not Applicable	

Data Availability

Data availability	Information included in the manuscript?	In which section is the information available? (Reagents and Tools Table, Materials and Methods, Figures, Data Availability Section)
Have **primary datasets** been deposited according to the journal's guidelines (see 'Data Deposition' section) and the respective accession numbers provided in the Data Availability Section?	Yes	Data availability section
Were **human clinical and genomic datasets** deposited in a public access-controlled repository in accordance to ethical obligations to the patients and to the applicable consent agreement?	Not Applicable	
Are **computational models** that are central and integral to a study available without restrictions in a machine-readable form? Were the relevant accession numbers or links provided?	Not Applicable	
If publicly available data were reused, provide the respective **data citations in the reference list**.	Not Applicable	
